# Epigenetic Plasticity Drives Carcinogenesis and Multi-Therapy Resistance in Multiple Myeloma

**DOI:** 10.21203/rs.3.rs-6306816/v1

**Published:** 2025-04-15

**Authors:** Rafael Renatino Canevarolo, Praneeth Reddy Sudalagunta, Mark B. Meads, Maria Silva, Xiaohong Zhao, Dario Magaletti, Raghunandan Reddy Alugubelli, Gabriel DeAvila, Erez Persi, Francesco Maura, Elissa T. Bell, Ryan T. Bishop, Christopher L. Cubitt, Samer S. Sansil, Wei Zhang, Jamie K. Teer, Mingxiang Teng, Sean J. Yoder, Erin M. Siegel, Bijal D. Shah, Taiga Nishihori, Lori A. Hazlehurst, Conor C. Lynch, Ola Landgren, Oliver Hampton, Robert A. Gatenby, Daniel M. Sullivan, Jason B. Brayer, William S. Dalton, John L. Cleveland, Melissa Alsina, Rachid Baz, Kenneth H. Shain, Ariosto Siqueira Silva

**Affiliations:** 1 Department of Metabolism and Physiology, H. Lee Moffitt Cancer Center & Research Institute, Tampa, FL, USA; 2 Department of Malignant Hematology, H. Lee Moffitt Cancer Center & Research Institute, Tampa, FL, USA; 3 Computational Biology Branch, Division of Intramural Research, National Library of Medicine, National Institutes of Health, Bethesda, MD, USA; 4 Division of Myeloma, Sylvester Comprehensive Cancer Center, University of Miami, Miami, FL, USA; 5 Department of Biological Science, Florida State University, Tallahassee, FL, USA; 6 Department of Tumor Microenvironment and Metastasis, H. Lee Moffitt Cancer Center & Research Institute, Tampa, FL, USA; 7 Immune Monitoring Core, H. Lee Moffitt Cancer Center & Research Institute, Tampa, Florida, USA; 8 Cancer Pharmacokinetics and Pharmacodynamics Core, H. Lee Moffitt Cancer Center & Research Institute, Tampa, FL, USA; 9 Department of Computer Science, University of Central Florida, Orlando, Florida, USA; 10 Department of Biostatistics & Bioinformatics, H. Lee Moffitt Cancer Center & Research Institute, Tampa, FL, USA; 11 Molecular Genomics Core, H. Lee Moffitt Cancer Center & Research Institute, Tampa, FL, USA; 12 Department of Cancer Epidemiology, H. Lee Moffitt Cancer Center & Research Institute, Tampa, FL, USA; 13 Department of Blood & Marrow Transplant and Cellular Therapies, H. Lee Moffitt Cancer Center & Research Institute, Tampa, FL, USA; 14 Department of Pharmaceutical Sciences, West Virginia University, Morgantown, WV, USA; 15 Aster Insights, Tampa, FL, USA; 16 Departments of Radiology and Integrated Mathematical Oncology, H. Lee Moffitt Cancer Center & Research Institute, Tampa, FL, USA

**Keywords:** multiple myeloma, epigenetic plasticity, carcinogenesis, malignant transformation, multi-therapy resistance, epigenetics, chromatin accessibility, H3K27me3, H3K27ac, transcription dysregulation, super enhancer, pioneer transcription factor, therapeutic target, MGUS, smoldering multiple myeloma, single cell multiomics

## Abstract

We demonstrate that carcinogenesis and multi-therapy resistance in multiple myeloma (MM)—a treatable yet incurable plasma cell malignancy—are driven by epigenetic dysregulation. In this new paradigm, genomic and cytogenetic events unlock epigenetic plasticity, reshaping MM cell biology to evade tumor microenvironment constraints and therapeutic pressure. These conclusions are derived from a newly assembled cohort of nearly 1,000 patients, spanning premalignant to late-stage refractory MM, comprehensively characterized at molecular and clinical levels. Our findings provide a unifying framework to explain inter-patient genomic heterogeneity and the emergence of therapy resistance in sequential samples without new genomic alterations. In conclusion, we propose targeting epigenetic plasticity-mediated plasma cell evasion as a promising therapeutic strategy in MM.

## Introduction

**Figure 1.**
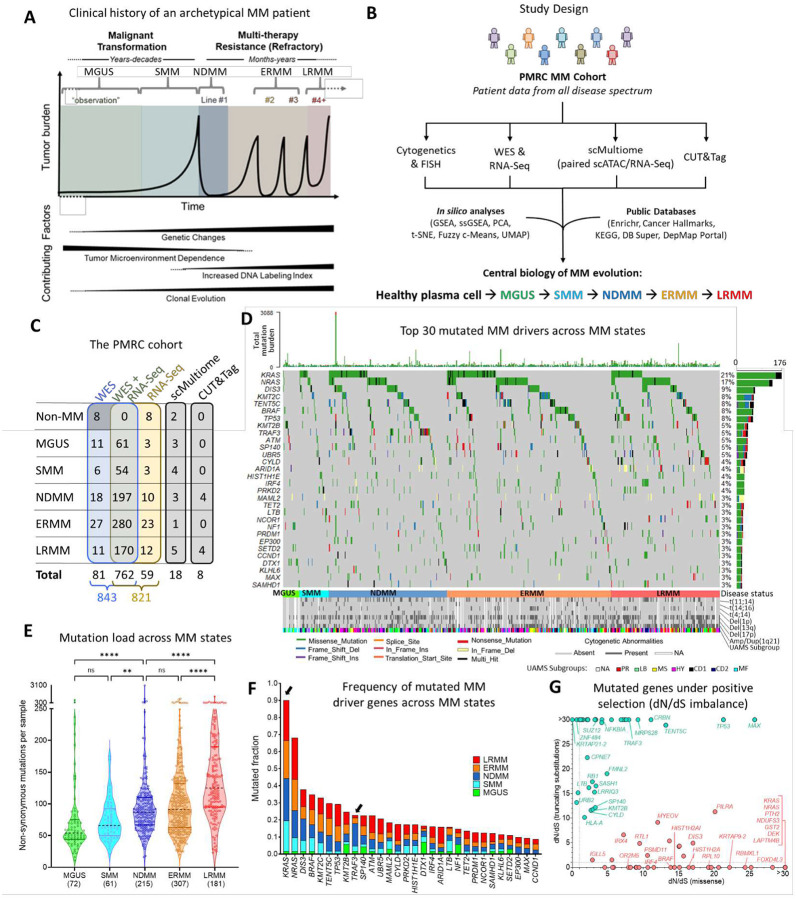
Study design and mutational landscape. **A**, Diagram of MM clinical evolution and associated changes. **B**, Integration of clinical and molecular data with public databases using analytical and visualization tools to uncover novel mechanisms in disease and drug resistance. **C**, Overview of molecular data available for the PMRC cohort. **D**, Oncoplot illustrating the top 30 mutated MM drivers in the cohort. **E**, Non-synonymous mutation load by disease states. Significance: (****) P<0.0001, (**) P<0.01 (Dunn’s test, Kruskal-Wallis). **F**, Frequency of top 30 mutated MM drivers across disease states. Highlighted, *KRAS* (increased from MGUS to SMM; adj. *p*=0.0627, Fisher’s Exact Test) and *TRAF3* (decreased from NDMM to ERMM; adj. *p*=0.0036). **G**, dN/dS imbalance for missense (gain-of-function, in red) and nonsense (loss-of-function, in green) mutations. Only genes with dN/dS>1 in at least one axis are shown.

Multiple myeloma (MM) is a treatable, yet incurable, malignancy of plasma cells (PCs) originating in the bone marrow. It is preceded by pre-malignant states—monoclonal gammopathy of undetermined significance (MGUS) and smoldering MM (SMM)^1,2^—with annual progression rates to MM of approximately 1% and 10%, respectively3. While newly diagnosed, therapy-naïve patients (NDMM) generally respond well to treatment, resistance to multiple therapies eventually develops^2,3^ (Figure 1A).

MM is marked by recurrent cytogenetic abnormalities and mutations that accumulate as the disease progresses. Certain genomic alterations (e.g., Amp1q21, Del(17p), t(4;14), *TP53* mutation), as well as transcriptomic-derived signatures^4–9^, are prognostically relevant. However, inter-patient heterogeneity^10–14^ complicates the correlation between genomic/transcriptomic traits and the biological phenotypes selected by the tumor microenvironment (TME)^15^ and therapy.

Although epigenetic dysregulation plays an increasing role in MM evolution^10,16,17^, its impact on disease progression and multi-therapy resistance remains poorly understood, partly due to the limited availability of public datasets encompassing premalignant and relapsed/refractory samples.

To address this challenge, we assembled the Pentecost Myeloma Research Center (PMRC) cohort—consisting of nearly 1,000 MM patients treated at the H. Lee Moffitt Cancer Center, including over 100 longitudinal paired biopsies, spanning all stages of the disease. Through an integrative analysis of clinical, mutational, transcriptional, and epigenetic data—independently validated by publicly available datasets—we uncovered key mechanisms driving MM evolution (Figure 1B).

Our unsupervised analysis determined that differential transcriptomes linked to carcinogenesis and late-stage refractory disease correlate with dynamic landscapes of the histone modifications H3K27me3 and H3K27ac, respectively. We propose that genomic and cytogenetic abnormalities, commonly associated with MM risk, unlock an epigenetic plasticity that allows MM cells to escape normal plasma cell biology, acquire TME-independence, and evade therapeutic window.

## Results

### Mutational landscape and new oncogene candidates of MM progression

The PMRC cohort comprises bone marrow aspirates (20 mL) and peripheral blood samples from patients across the MM spectrum, treated at the Moffitt Cancer Center between 2011 and 2023^4,14,18,19^, with consent obtained under the Total Cancer Care Protocol (TCC)^20^. PCs were isolated via positive selection (CD138+), achieving a mean post-selection enrichment of 95.3% (CI: 88.1%–100%), and subsequently molecularly characterized. Peripheral blood mononuclear cells served as the germline reference. Patient demographics, disease state at biopsy, FISH/cytogenetics, and treatment details were abstracted from clinical records (Table S1). High PC enrichment was validated through bulk and single-cell gene expression analyses (see Methods, “Sample Purity Assessment”, and Figure S1). Samples underwent paired WES and RNA-Seq, including sequential biopsies. A subset of samples was further analyzed using scMultiome and CUT&Tag to validate findings from the main cohort (Figure 1C). The median follow-up duration was 24.9 months.

Several studies have identified driver gene mutations associated with MM^12,14,21^, highlighting their role in myeloma development and therapy resistance. Analysis of the PMRC cohort’s mutational landscape confirmed the presence of these previously reported driver mutations, with mutational frequencies comparable to four independent cohorts^22–25^, including the MMRF-CoMMpass Study (Table S2). In the PMRC cohort, 678 samples (81.2%) harbored at least one non-synonymous mutation in the 81 established MM driver genes^26^. The most frequently mutated drivers were *KRAS* (21.1%), *NRAS* (16.8%), and *DIS3* (8.9%) (Figure 1D). Additionally, mutation rates observed in the PMRC SMM subcohort (n=60) were consistent with those reported in other SMM cohorts^5,16^ (Table S2).

MM is characterized by steady increase in mutational load and frequency with disease progression^10^ (Figure 1E), but only a limited set of driver genes demonstrated altered rates across disease state. *KRAS* mutations increased from 1.4% in MGUS to 18% in SMM (adj. p-value=0.062). In contrast, *TRAF3* mutations decreased from 13.1% in NDMM to 3.3% in early relapse MM (ERMM, 1–3 lines of therapy, adj. p-value=0.0036) and to 1.7% in late relapse MM (LRMM, ≥4 lines of therapy, adj. p-value=0.0012), suggesting negative selection by therapy (Figure 1F). We also identified significant associations between cytogenetic abnormalities and driver mutations, such as *TP53* with Del(17p) and *DIS3*, *PRKD2*, and *FGFR3* with t(4;14)^21^ (Table S3).

To complete the identification of potential drivers, we analyzed the imbalance between non-synonymous (missense or nonsense) and synonymous mutations (dN/dS)^27^ and identified 107 genes with a disproportionate number of missense or truncating mutations demonstrating positive selection in MM (dN/dS>1). In addition to confirming frequently mutated MM genes (e.g., *KRAS*, *BRAF*, *TENT5C*, and *TP53*), this approach identified low-frequency potential new driver mutations (e.g., *SUZ12*, *CRBN*, *SELENOT*, and *ZNF484*, Figure 1G and Table S4). While dN/dS imbalance in some genes was dominated by either truncation (tumor suppressor genes; e.g., *RB1*) or missense mutations (oncogenes; e.g., *IRF4*), a significant number of genes presented both (e.g., *TP53*), suggesting a more ambivalent role. In summary, we have identified a list of novel putative low-frequency driver mutations in MM samples across the disease spectrum, with their specific role in either progression to active or relapsed/refractory disease still to be determined. Next, we interrogated differential biology and driver mechanisms across these two transitions through transcriptomics.

### MM progression correlates with increased TME independence and cell cycle/metabolism transcriptional programs

**Figure 2.**
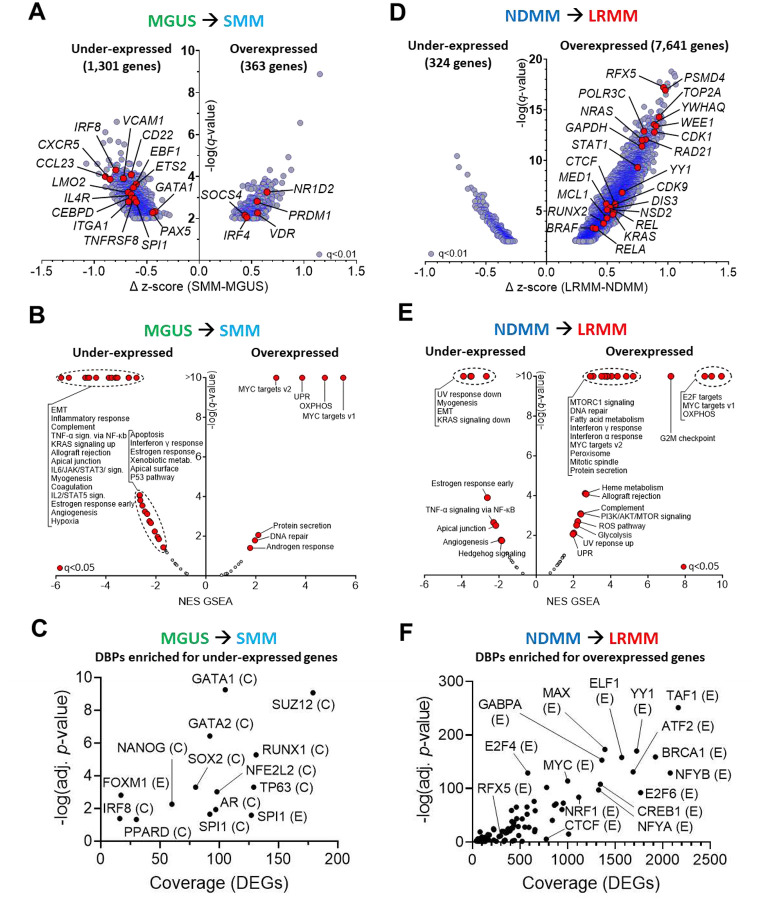
Transcriptomic changes across MM evolution. **A,** Differentially expressed genes (DEGs) in the MGUS-to-SMM transition, z-normalized (Log_2_(FPKM+10^−3^)) and analyzed with unpaired t-tests (q-value<1%). **B**, Gene set enrichment analysis (GSEA) for Cancer Hallmarks among these DEGs. **C**, DNA-binding proteins (DBPs) enriched (q<0.05) for genes under-expressed in the MGUS-to-SMM transition, based on ENCODE (E) and ChEA (C) data. **D**, DEGs in the NDMM-to-LRMM comparison. **E**, Idem (**B)**, for DEGs in LRMM vs. NDMM. **F**, DBPs enriched for genes overexpressed in the NDMM-to-LRMM comparison. NES=normalized enrichment score. Genes and pathways of interest are labeled and highlighted in red.

We examined the protein-coding transcriptome from patient samples ranging from MGUS to LRMM (Figure 1C) to identify biological mechanisms driving malignant transformation and therapy resistance. We identified 1,301 genes with decreased expression, and 363 genes with increased expression, in SMM relative to MGUS samples (unpaired t-tests, FDR=1%, Figure 2A and Table S5). Gene set enrichment analyses (GSEA^28^) of the aforementioned differentially expressed genes (DEG) revealed decreased enrichment of pathways related to inflammation (e.g., *CCL23*), cytokine signaling (e.g., *IL4R*), adhesion (e.g., *VCAM1*, *ITGA1*), and apoptosis (e.g., *TNFRSF8*), suggesting reduced cell-cell (paracrine) signaling and a shift towards tumor-microenvironment (TME) independence^2,15^ (Figure 2B for Cancer Hallmarks, Figure S2A for KEGG Pathways, and Table S6 for the enrichment score of all pathways). Conversely, MYC targets, unfolded protein response (UPR) and oxidative phosphorylation (OXPHOS) were among the few pathways enriched among overexpressed genes. Enrichment analysis for DNA-binding proteins (DBPs) in under-expressed DEGs pointed to (i) SUZ12, a component of the polycomb repressive complex 2 (PRC2), responsible for epigenetic transcription repression through histone methylation^29^ (e.g., H3K27me3); (ii) NANOG and SOX2, implied in epigenetic-mediated plasticity^30–33^; and (iii) RUNX1, GATA1/2, and SPI1, involved in cell fate decision during hematopoiesis^34–36^ (Figure 2C and Table S7). MAX, MYC and NANOG were the only DBPs enriched for the overexpressed genes in SMM, compared to MGUS samples. Interestingly, there were no statistically significant differences in expression of genes or pathways between NDMM and SMM samples after multiple testing correction (FDR = 1%), suggesting that the distinction between these two states may be primarily clinical in nature (e.g., tumor burden, symptoms), rather than driven by biological alterations in tumor cells.

In contrast, the comparison between LRMM and NDMM identified 324 under-expressed and 7,641 overexpressed genes (Figure 2D and Table S5). Noticeable among overexpressed DEGs, we found frequently mutated oncogenes (e.g. *NRAS*, *KRAS*, *BRAF*), as well as essential genes involved in transcription initiation (e.g. *CDK9*), formation of transcription activation domains^37,38^ (TADs, e.g. *CTCF*), and mediator complex (*MED1*) — the latter two being crucial for enhancer activity^39,40^. In addition, pathways associated with cell proliferation and energy metabolism^18,41–43^ were enriched in LRMM (Figure 2E for Cancer Hallmarks, Figure S2B for KEGG Pathways, and Table S6 for all pathways). The DBPs enriched for overexpressed DEGs consisted of transcription factors (TFs) involved in telomere formation (e.g., RFX5, CEBPB, MAX, SP1, MYC)^44^, TAD formation (e.g., YY1, CTCF)^37,38,40,45^, and cellular differentiation (e.g., GABPA, NRF1)^45–47^ (Figure 2F and Table S7). Collectively, this transcriptional analysis indicates that the transition from premalignant stage to NDMM is accompanied by reduced expression of genes involved in cytokine/inflammation signaling and cell adhesion pathways, leading to TME independence. This occurs alongside differential PRC2 activity and is primarily observed during the transition from MGUS to SMM. In contrast, there is a stepwise increase in overall overexpression of genes associated with pathways involved in transcription, cell cycle regulation, DNA repair, and metabolism as the disease progresses from NDMM to LRMM.

### Transcriptomics are prognostic in premalignant and active MM

In the previous section, we demonstrated the differential expression of biological pathways across premalignant, newly diagnosed, and therapy-refractory disease stages. Next, we examined whether the expression of these pathways holds prognostic value in MM and compared our findings with previous studies on transcriptome-based prognostic biomarkers in MM^48–52^.

**Figure 3.**
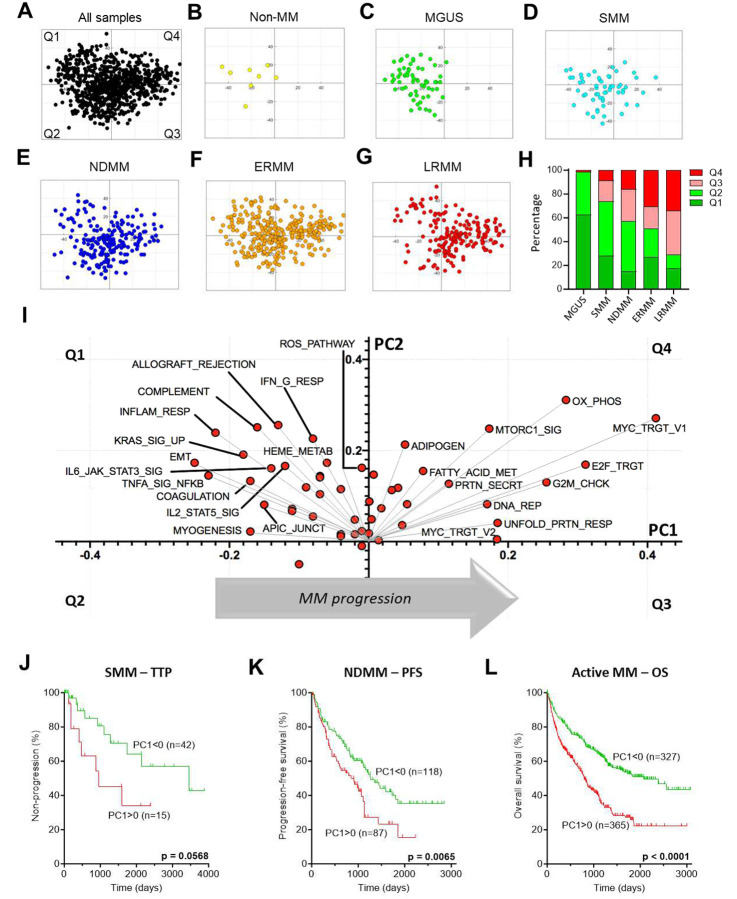
Principal component analysis identifies gene expression signature associated with outcome. **A**, PCA score of 821 samples with RNA-Seq data, with ssGSEA enrichment score of Cancer Hallmarks as variables. (**B–G**), Samples visualized according to their disease status. **H**, Distribution of samples across PCA quadrants. **I**, PCA loading plot showing relative contribution of each Cancer Hallmarks to sample distribution, with MM evolution along PC1. (**J–L**), Association of PC1 with (**J**) TTP in SMM, (**K**) PFS in NDMM (PFS data missing for 2 patients) and (**L**) OS in Active MM. *P*-values from Log-rank (Mantel-Cox) tests.

We conducted single-sample gene set enrichment analysis (ssGSEA^28^) for 821 RNA-Seq CD138^+^ bone marrow aspirate samples, ranging from MGUS to LRMM, including 8 controls (one healthy bone marrow and 7 solitary plasmacytomas with no malignant PC involvement). Principal component analysis (PCA) was conducted on normalized enrichment scores (NES) using the 50 Cancer Hallmarks as variables^53^ (Figure 3A). Non-MM PCs and MGUS samples primarily clustered in the left quadrants (quadrants 1 and 2; Q1, Q2; PC1<0) (Figures 3B and 3C). In contrast, SMM, NDMM and ERMM samples were progressively less concentrated in these quadrants (Figures 3D and 3F), while LRMM samples were enriched in the right quadrants (quadrants 3 and 4; Q3, Q4; PC1>0) (Figure 3G). This indicates a transcriptional diversity within clinically assigned disease stages, and that MM evolution occurs along PC1, with samples transitioning from PC1<0 to PC1>0 (Figure 3H).

To illustrate the contribution of each Cancer Hallmark in PC1/PC2 (and thus to transcriptomic variance), we generated a loading plot, representing each Hallmark as a vector originating from the center of the multidimensional space, with longer vectors indicating a greater contribution to the two principal components. This analysis revealed two groups of vectors: the first pointing towards Q1, representing pathways associated with TME dependence and inflammation; the second pointing towards Q4, encompassing pathways related to cell cycle, DNA repair, and metabolism (Figure 3I). These findings align with the differentially enriched pathways observed between premalignant stages (SMM vs. MGUS, Figure 2B) and in relapsed disease (LRMM vs. NDMM, Figure 2E).

Furthermore, we observed that positive PC1 values were associated with shorter time to progression (TTP) in SMM samples, shorter progression-free survival (PFS) in NDMM, and shorter overall survival (OS) in Active MM (NDMM+ERMM+LRMM) patients, as determined by the Log-rank (Mantel-Cox) test (Figure 3J to 3L). PC1>0 was also associated with shorter OS in NDMM and ERMM, but not in LRMM (Figures S3A to S3C), likely due to the high proportion (~70%) of PC1-positive samples in this disease state. PCA coordinates, clinical parameters (TTP, PFS, OS, demographics), cytogenetics, and driver mutation status for all samples with available transcriptomic information are provided in Table S8.

Next, we investigated the overlap of these findings with pre-existing transcriptional classifications, such as UAMS subgroups^54^. As expected, there was a strong correlation between three UAMS subgroups and specific cytogenetic abnormalities in our cohort: (i) the “MS” subgroup, characterized by FGFR3 overexpression, was associated with t(4;14); (ii) the “CD-1” and “CD-2” subgroups, marked by CCND1 overexpression, were linked to t(11;14); and (iii) the “MF” subgroup, defined by MAF overexpression, was associated with t(14;16) (p<0.001 for all comparisons, Fisher’s Exact test) (Figure 1D). In the PCA transcriptomic space, most samples classified as “MF” were in the PC1<0 region (p<0.0001; Fisher’s Exact Test), whereas the majority of samples from the “PR” (p<0.0001), “CD-1” (p<0.0001), and “LB”(p=0.017) subgroups were predominantly concentrated in Q3 and Q4 (Figures S4A to S4I). Furthermore, over 60% of MGUS samples were classified as “MF”, whereas the proportion of samples classified as “PR” steadily increased with disease progression (Figure S4J). Among NDMM patient samples, those classified as “MF” and “HY” presented the longest PFS and OS, while the “PR”, “LB” and “MS” subgroups had the poorest outcomes (Figures S4K and S4L). Finally, we demonstrated that the expression (ssGSEA NES) of the gene set defining the high-risk subtype “PR”, positively correlates with Cancer Hallmarks linked to PC1>0 (e.g., OXPHOS, G2M Checkpoint, MYC Targets, etc.), and inversely correlates with those associated with PC1<0 (e.g., EMT, Inflammatory Response, etc.) (Figure S4M). In summary, our unsupervised transcriptomic-based stratification approach aligns with previously described signatures, while possibly allowing risk stratification across disease states.

To test this hypothesis, we combined dichotomized PC1 values (positive or negative) with established MM risk factors — such as driver mutations, cytogenetic abnormalities, and demographic characteristics — using univariable Cox Proportional Hazard (Cox PH) models to evaluate their prognostic significance. Our analysis revealed that PC1>0 was associated with shorter TTP in SMM (p=0.065), shorter PFS in NDMM (p=0.0071), and shorter OS in NDMM (p=0.034), ERMM (p=0.00012), and Active MM (p=2.5e-10) (Table S9). The analysis also confirmed established clinical and molecular predictors of outcomes. For instance, *NRAS* mutation^16^, bone marrow PC count, and Mayo 2018 index^55^ (Figure S3D) were associated with shorter TTP in SMM samples. In NDMM samples, poor outcomes were associated with ISS/R-ISS indices^56^ (Figures S3E and S3F), Amp1q21, Double hit (more than 1 high-risk feature^57^), t(4;14), and mutations in *PIK3CA* (shorter PFS), and in *RPRD1B*, *CREBBP*, and *ZNF292* (shorter OS). Conversely, younger age at biopsy (<55 years) and *TRAF3* mutation^58^ were associated with longer PFS and OS. Interestingly, *TRAF3* mutation frequency was significantly higher in NDMM samples compared to ERMM or LRMM (Figure 1), suggesting an association between *TRAF3* mutation and MM therapy sensitivity^59,60^. In LRMM, Double hit, Del(13q), Del(17p), and mutations in *TP53* or *SAMHD1* were associated with shorter OS, whereas female gender and mutations in *PTPN11* and *PRDM1* were linked to longer OS.

Multivariable Cox PH models identified PC1>0 as an independent predictor of inferior OS in both ERMM (p=0.0041) and Active MM (p=0.0013), while also showing a trend towards shorter PFS and OS in NDMM. Table S9 lists the significance of all features tested in univariable and multivariable Cox PH models, and Figure S5 presents forest plots of the multivariable Cox PH models’ significant features by disease state. Collectively, these results indicate that the transcriptomic signatures corresponding to positive PC1 coordinates are consistently associated with poorer outcomes across different disease states.

These findings were tested in the MMRF-CoMMpass cohort^61^: PCA analysis was performed with ssGSEA NES for Cancer Hallmarks from 704 NDMM samples, and revealed a distribution of loadings along the PC1 axis comparable to our cohort (Figures S6A and S6B), despite CoMMpass’ enrichment for NDMM samples. Consistent with the analysis of the PMRC cohort, positive PC1 coordinates were associated with shorter PFS (p=0.012) and showed a borderline association with shorter OS (p=0.104) (Figures S6C and S6D).

### Sequential biopsies recapitulate population-level alterations

The PRMC cohort includes sequential biopsies from 116 patients, providing a unique opportunity to study transcriptional dynamics across multiple disease states through clinical, WES, and RNA-Seq data. Key examples include one MGUS patient with two biopsies (Figure S7A), two patients progressing from SMM to NDMM (Figures S7B and S7C), three patients with biopsies taken during various phases of active disease (Figures S7D, S7E and S7F), and one patient with six biopsies spanning ERMM and LRMM (Figure S7G). Across these cases, ssGSEA analysis showed a consistent trend of increased expression in Hallmarks linked to cell proliferation, DNA repair, energy metabolism, and environment-mediated multidrug resistance^2^ (EMDR) during disease progression and subsequent relapses. Notably, these transcriptomic changes could occur independently of observable cytogenetic abnormalities or mutations, suggesting that transcriptomic reprogramming can arise without alterations in these traits. Interestingly, as observed by Skerget *et al*.^61^, while UAMS-based transcriptomic classifications generally remain stable across sequential samples (e.g., Figures S7A to S7F), transitions to PR (i.e. high-risk, PC1>0) can occur in LRMM samples (e.g., Figure S7G), demonstrating that biological programing changes with therapy.

### Epigenetic modifications as driving forces of aberrant transcriptional patterns across MM evolution

**Figure 4.**
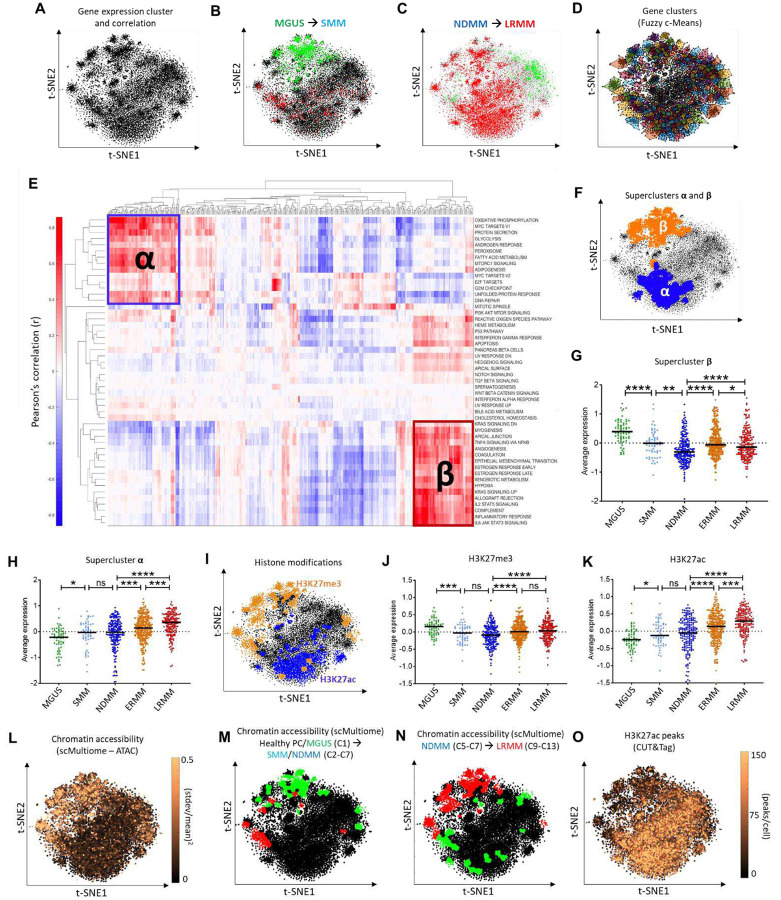
Topological map of transcriptional regulation and epigenetic controls. **A**, Topological map of MM transcription (t-SNE plot of 16,738 expressed genes based on z-normalized Log2(FPKM+10^−3) values). **B**, Projection of under-expressed (green) and overexpressed (red) genes in the MGUS-to-SMM transition. **C**, Projection of DEGs in the NDMM-to-LRMM comparison. **D**, Transcriptional map divided into 500 gene clusters via Fuzzy c-Means. **E**, Hierarchical clustering of Pearson’s correlations between ssGSEA NES of the 500 gene clusters and Cancer Hallmarks. Superclusters **α** and **β** are indicated. **F,** Locations of superclusters **α** and **β** on the topological map. **G–H**, Average expression of genes in superclusters **β** (**G**) and **α** (**H**) across MM states. **I**, Gene clusters enriched for H3K27me3 and H3K27ac (publicly available ChIP-Seq data). **J–K,** Average expression of (**J**) H3K27me3- and (**K**) H3K27ac-controlled genes across MM states. **L**, Chromatin accessibility variation for individual genes across 18 samples with scMultiome data. **M–N**, Topological map showing gene clusters with differential accessibility in scMultiome’s clusters (**M**) C2–C7 (SMM and NDMM) *versus* C1 (mostly healthy PCs and MGUS cells); and (**N**) C9–C13 (LRMM) *versus* C5–C7 (NDMM). red: increased accessibility, green: decreased accessibility. **O**, Average H3K27ac peaks per gene from CUT&Tag data. (****) P<0.0001, (***) P<0.001, (**) P<0.01, (*) P<0.05, Mann-Whitney test.

To uncover mechanisms driving transcriptomic changes in MM evolution, we applied a dimensionality reduction technique, t-distributed stochastic neighbor embedding (t-SNE^62^), to RNA-Seq data from the PMRC cohort^63^, grouping genes by co-expression across the disease spectrum (Figure 4A). Genes differentially expressed between MGUS and SMM predominantly clustered in the upper portion of the t-SNE plot (Figure 4B), whereas those between NDMM and LRMM were distributed more broadly (Figure 4C).

We used Fuzzy c-Means^64^ to segment the transcriptional map into 500 clusters of highly co-expressing genes (Figure 4D), hypothesizing that genes within each cluster share common regulatory mechanisms. This combined approach (t-SNE + Fuzzy c-Means) demonstrated equivalent or superior preservation of biological and functional attributes compared to other methods (Figure S8) while enabling effective data visualization^63^. Genes associated with Cancer Hallmarks and KEGG Pathways could either form cohesive clusters (e.g., Ribosome, Proteasome) or exhibit dispersed transcription patterns across the map (e.g., Focal Adhesion) (Figures S9A to S9G), highlighting the heterogeneous regulation of curated gene sets.

To elucidate the biology associated with these unsupervised gene clusters, we performed hierarchical clustering of Pearson correlations between ssGSEA scores of the 500 gene clusters and Cancer Hallmarks across all PMRC samples. This analysis revealed two superclusters, here termed ‘**α**’ and ‘**β**’, enriched for distinct Cancer Hallmarks: supercluster **α** comprised 14 Hallmarks related to proliferation, DNA repair, and energy metabolism, while supercluster **β** included 17 Hallmarks related to cell adhesion, inflammation, and cytokine signaling (Figure 4E). Mapping these superclusters onto the transcriptional map revealed their spatial distribution on the t-SNE plot (Figure 4F), aligning with regions of DEGs between disease states and suggesting that distinct regulatory mechanisms govern their expression. Additionally, supercluster **β** genes’ expression decreased from premalignant to active disease but increased from NDMM to LRMM (yet remaining lower than in the premalignant states) (Figure 4G). In contrast, supercluster **α**’s expression steadily increased throughout the MM therapeutic course, peaking in LRMM (Figure 4H). Univariable Cox PH models indicated that overexpression of supercluster **α** was associated with poor prognosis (shorter OS in ERMM, LRMM and Active MM), whereas high expression of genes in supercluster **β** was predictive of better outcomes (longer TTP in SMM, PFS in NDMM and OS in ERMM and Active MM) (Table S9).

The coalescence of DEGs across disease states into superclusters, along with their enrichment for upstream TFs associated with epigenetic modifiers (e.g., SUZ12; Figures 2C and S9H) suggested that epigenetic mechanism may drive transcriptional dysregulation in MM. Enrichment analysis of histone modifications in the t-SNE’s 500 gene clusters, using publicly available ChIP-Seq data via *Enrichr*^65^, identified clusters enriched for histone H3 lysine 27 trimethylation (H3K27me3) and acetylation (H3K27ac). These clusters were spatially distributed on the transcriptional map overlapping with the regions of superclusters **β** and **α**, respectively — demonstrating mutually exclusive enrichment patterns (Figure 4I). The mean expression of putatively H3K27me3-controlled genes decreased across premalignant states (Figure 4J), while genes enriched for H3K27ac showed increasing expression throughout all MM states (Figure 4K). H3K27me3 represses gene expression by reducing chromatin accessibility, whereas H3K27ac promotes transcription at both proximal and distal regions of transcription start sites^66–68^. Our results highlight that these two histone modifications represent opposing epigenetic regulatory mechanisms critical to early myelomagenesis (H3K27me3) and relapsed/refractory disease (H3K27ac).

### Single cell multiomic analysis demonstrates the association between epigenetic reprogramming and transcriptional changes across premalignant states

To test the hypothesis of epigenetic regulation of DEGs associated with MM progression, we conducted scMultiome (single-cell, paired RNA/ATAC-Seq) on 18 CD138^+^ BM aspirate samples from 2 healthy donors, 3 MGUS, 4 SMM, 3 NDMM (including a sequential sample from SMM), 1 ERMM, and 5 LRMM patients, ranging from 5,361 to 11,293 cells per sample (patients’ demographics and cytogenetics in Table S10). We investigated genes with the highest variation in chromatin accessibility (estimated by scATAC-Seq signal) across these samples to identify any overlap with previously identified genes putatively regulated by H3K27me3. We calculated *pseudobulk* ATAC-Seq^69^ values for each gene in each sample, then assessed the normalized variance of peak counts for each gene and projected these values onto the t-SNE topology map. This analysis revealed a remarkable overlap between regions of high differential accessibility (normalized SD≥0.5, Figure 4L), supercluster **β** (Figure 4F), and genes predicted to be regulated by H3K27me3 based on ChIP-Seq data meta-analysis (Figure 4I). Additionally, gene clusters enriched for differentially accessible genes between healthy PCs/MGUS and SMM/NDMM (Figure 4M), as well as between NDMM and LRMM (Figure 4N) samples agreed with differential expression (bulk RNA-Seq) in these two critical transitions (Figures 4B and 4C, respectively).

**Figure 5.**
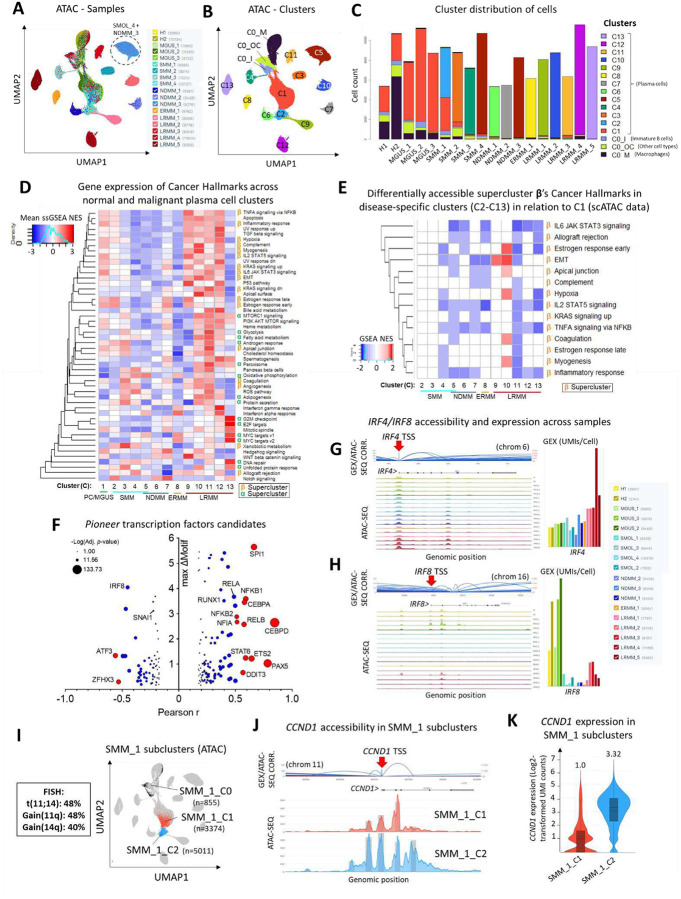
Single-cell chromatin accessibility and gene expression across the MM spectrum. scMultiome analysis was conducted on 2 healthy donors, 3 MGUS, 4 SMM, 3 NDMM, 1 ERMM and 5 LRMM samples to validate bulk data and in silico predictions. **A**, UMAP plot of chromatin accessibility (ATAC) colored by sample origin. **B**, UMAP plot showing distinct cell clusters. **C**, Distribution of cells across clusters. **D**, Mean ssGSEA enrichment scores (ES) for Cancer Hallmarks per cluster, from gene expression data. **E**, GSEA NES (ΔC2–13,C1) for supercluster **β** Cancer Hallmarks among differentially accessible genes in clusters C2–C13 in relation to C1. Only statistically significant Hallmarks in at least one comparison are shown. **F**, Pioneer TF candidates in MM. The x-axis shows the Pearson correlation between TF gene expression and target gene accessibility, while the y-axis shows the variation in TF motif accessibility. Red labels indicate TFs with Pearson’s correlation >0.5 (activators) or <−0.5 (repressors). Bubble size reflects adjusted p-values for correlation. **G-H**, Chromatin accessibility and expression of (**G**) *IRF4* and (**H**) *IRF8* across samples (cluster C0 excluded). TSS = transcription starting site. **I**, Localization of SMM_1 cells in clusters C0, C1 and C2. Cytogenetic abnormalities identified by FISH with percentage of cells harboring them are indicated in the box. **J**, *CCND1* promoter accessibility across SMM_1 subclusters. **K**, *CCND1* expression across SMM_1 subclusters. Median values (in Log2) are displayed.

We generated Uniform Manifold Approximation and Projection for Dimension Reduction (UMAP) plots to visualize sample clustering according to chromatin accessibility (scATAC-Seq compartment, Figure 5A) and gene expression (Figure S10A) — for consistency purposes, all image overlays in this single cell analysis depict the scATAC-Seq compartment UMAP geometry, even when projecting transcriptomic data. We identified 12 cell clusters (C2 to C13) that exclusively consist of cells derived from individual MM samples, alongside two larger clusters (C0 and C1) containing cells from all samples, highlighting shared and sample-specific features within the MM cellular landscape (Figures 5B, 5C, and S10B). Gene marker analysis revealed that cluster C0 mainly consisted of macrophages (*IL1B*_*HIGH*_), T cells precursors (*BCL11B*_*HIGH*_), NK and CD8+ cells (*NKG7*_*HIGH*_), erythroid cells (*SLC4A1*_*HIGH*_), and immature B cells (*AFF3*_*HIGH*_, *EBF1*_*HIGH*_), while clusters C1-C13 consisted of mature PCs (*IRF4*_HIGH_) (Figures S10C to S10J). PCs from all healthy donors and MGUS patients co-localized in cluster C1, the only difference between the two groups being the percentage of PCs post CD138-enrichment (~25–50% vs. 75–90%, respectively). SMM, NDMM, ERMM and LRMM samples exhibited PCs in sample-specific clusters (C2 to C13, Figures 5B and 5C), suggesting that the transition from MGUS to SMM is the crucial step in departure from normal PC biology. To further distinguish healthy PCs from premalignant blasts, we analyzed immunoglobulin gene expression across all cells from each sample to identify signs of clonality. Our analysis revealed that while PCs from healthy donors, MGUS_1, and MGUS_3 samples appeared to be composed of multiple clones, MGUS_2, all SMM, and active disease samples were dominated by a single clone (Figure S11).

Single-sample GSEA analysis of scRNA-Seq for both Cancer Hallmarks (Figure 5D) and TFs (Figure S10K) agreed with bulk RNA-Seq findings. This analysis confirmed that supercluster **β** genes were progressively under-expressed from premalignant stages (clusters C1-C5) to NDMM (C5–C7) and subsequently overexpressed — along with supercluster **α** genes — in LRMM (C9–C13), mimicking bulk data (Figures 4G and 4H). Furthermore, scATAC-Seq analysis revealed a consistent decrease in chromatin accessibility for supercluster **β** Hallmarks across all disease-specific clusters (except C9 and C10) compared to healthy PCs/MGUS (C1) (Figure 5E). In contrast, differential chromatin accessibility for supercluster **α** genes was limited to a few Hallmarks and clusters (Figure S10L), suggesting that chromatin accessibility is a primary regulator of supercluster **β** expression but plays a less significant role in regulating supercluster **α**. In conclusion, these findings underscore the role of epigenetic reprogramming, particularly decreased chromatin accessibility regulated by H3K27me3, in driving transcriptional changes during malignant transformation from MGUS to myeloma (SMM and NDMM).

Pioneer TFs are characterized by the ability to bind to heterochromatin, increase binding site accessibility to other DNA binding proteins, and ultimately enhance transcription^70^. To investigate putative pioneer TFs driving the observed epigenetic and transcriptomic changes between PCs and MM, we examined TFs whose expression and chromatin accessibility of target genes were significantly correlated. Among the identified pioneer TF candidates (Figure 5F and Table S11), SPI1, IRF8, NFE2L2, and RUNX1 were also enriched for target genes that are under-expressed in the MGUS-to-SMM transition in the main cohort of patients (Figure 2C). Both *SPI1* and *IRF8* were under-expressed during this disease state transition and have been previously characterized as drivers of PC differentiation^71^. Additionally, *IRF8* transcription can be epigenetically regulated by the PRC2 complex^72^, is epigenetically silenced in MM cell lines^73^, and is part of a negative feedback loop with *IRF4*^74^, a critical driver of MM^10^, as demonstrated in Figures 5G and 5H.

SUZ12 and other components of the PRC2 complex undergo post-translational modifications^75^ and play a role in the mutual regulation of cell cycle-related genes^76^. Many of these genes are linked to common cytogenetic abnormalities and mutations, including *CCND1* (associated with t(11;14)), *CKS1B* (Amp1q21), *TP53* (Del(17p)), *RB1* (Del(13q), as well as *RB1* and *TP53* mutations. To further explore these connections, we conducted pseudotime analysis in the subsequent section to investigate the cytogenetic and genetic events potentially driving transitions between clusters within the scMultiomic framework.

### Pseudotime analysis associates epigenetic and transcriptomic changes to MM genomic and cytogenetic driver events

We integrated scRNA-Seq and scATAC-Seq as proxies for gene expression and copy number variation, respectively, alongside standard-of-care FISH, to analyze samples from SMM to LRMM patients. For instance, the t(11;14)-positive SMM_1 sample exhibited cells distributed across both the healthy PC/MGUS cluster (C1) and a sample-specific cluster (C2) (Figure 5I). Further examination of gene expression (Figure 5J) and chromatin accessibility (Figure 5K) for CCND1, a hallmark of t(11;14), indicated that the translocation was restricted to cells within C2. This suggests that t(11;14) may drive premalignant progression in this sample. Furthermore, we compared single-cell copy number analysis across all samples using scATAC-Seq (epiAneufinder^77^, Figures S12 to S24), scRNA-Seq (inferCNV^78^, Figure S25), and FISH data (Table S10). Despite the inherent limitations of these technologies — such as scRNA-Seq being restricted to exons, 90% of scATAC-Seq peaks being intergenic, distal or intronic, and FISH probes targeting only a limited set of genes — their combined use proved effective in identifying genomic abnormalities within sample-specific clusters that were absent in the healthy PC/MGUS cluster (Table S12). In summary, our scMultiomic analysis indicates that while copy number and cytogenetic abnormalities are sample-specific, they may serve as initiating events. These events appear to drive common epigenetic and transcriptomic changes, facilitating the transition from MGUS to SMM/NDMM through the post-translational activation of the PRC2 complex, which disrupts the natural PC differentiation program.

### Refractory MM is characterized by increased transcriptional activity in H3K27ac-regulated transcriptional domains

Integrative analysis of bulk RNA-seq data revealed that a significant number of genes differentially expressed between NDMM and LRMM samples were enriched for H3K27ac signals (Figures 4C and 4I), as identified in publicly available ChIP-seq databases. These findings suggest that this epigenetic modification plays a critical role in driving relapsed/refractory MM. To validate these predictions, H3K27ac CUT&Tag was performed on samples from 4 NDMM and 4 LRMM patients (patient demographics are provided in Table S13). As shown in Figure 4O, genes with the highest H3K27ac CUT&Tag signal (mean values from 8 samples) were predominantly concentrated in supercluster **α** region (Figure 4F). The complete list of H3K27ac peaks is provided in Table S14.

**Figure 6.**
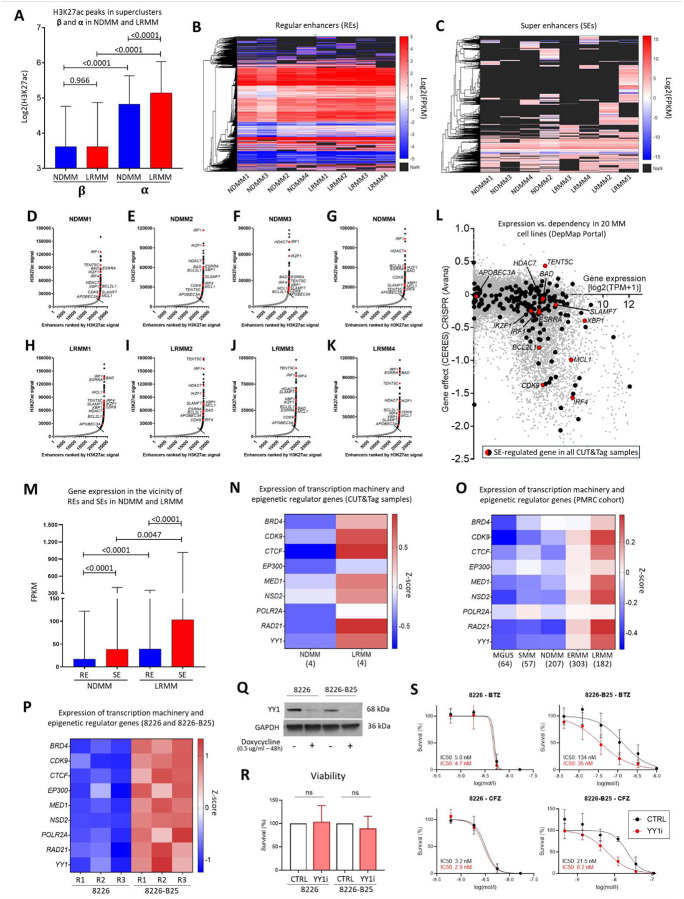
Relapsed MM shows increased H3K27ac and altered transcriptional regulation. **A,** Average H3K27ac peaks near genes from superclusters **β** and **α** in NDMM and LRMM, analyzed across 4 samples per group; p-values calculated using unpaired Student’s t-test. **B-C**, Gene expression near (**B**) regular enhancers (REs) or (**C**) super enhancers (SEs) within 0.5 Mb. **D–K**, SEs (black dots) identified by ROSE, separated from REs (gray dots) by a trace; 13 of 213 SE-regulated genes shared by CUT&Tag samples are highlighted in red. **L**, Gene expression vs. lethality in 20 MM cell lines from the DepMap Portal; highlighted in black and red (labeled) are the 213 SE-regulated genes from CUT&Tag samples. **M**, Gene expression near REs and SEs in NDMM and LRMM; p-values calculated using unpaired Student’s t-test. **N-P**, Z-normalized expression of transcription machinery and cofactors genes in (**N**) CUT&Tag samples, (**O**) the PMRC cohort, and (**P**) 8226 and 8226-B25 (PI-resistant) cell lines; sample numbers are shown in parentheses. **Q**, Western blot of YY1 in 8226 and 8226-B25 following *YY1* knockdown using shRNA. **R**, Viability of 8226 and 8226-B25 cells post-*YY1* knockdown normalized to shRNA control. **S**, Survival curves and IC50 estimates for 8226 and 8226-B25 cells with *YY1* knockdown or scramble shRNA.

We observed that H3K27ac signals were higher in genes from supercluster **α** in LRMM compared to NDMM samples, whereas genes from supercluster **β** exhibited lower overall H3K27ac signals with no significant difference between these disease states (Figure 6A). To further investigate such abnormal transcriptional activity, we used the Rank Ordering of Super-Enhancers (ROSE)^79^ algorithm to distinguish super enhancers (SEs) from regular enhancers (REs) based on H3K27ac signal intensity and length (Table S15). This analysis revealed that REs were largely conserved (Figure 6B), while the activation status of SEs varied significantly across samples (Figure 6C). There was no significant difference in the number of genes regulated by SEs (Figure S26) or in identifiable patterns associated with disease state, except for *CRB2* — a gene associated with drug resistance and poor prognosis in glioblastoma^80^ —, which was regulated by an active SE in all LRMM samples but in none of the NDMM samples.

We found that 213 of the 2,818 unique SE-regulated genes (Table S16) were conserved across the 8 samples, suggesting an essential role in PC and MM biology. These genes included TFs (*TENT5C*^81^, *XBP1*^82,83^, *IRF1*^68^, *IRF4*^83,84^*, IKZF1*^85–87^*, ESRRA*^88^), members of the BCL2 family (*MCL1*, *BAD*, *BCL2L1*)^89^, APOBEC3 family members (e.g., *APOBEC3A*)^90^, a histone deacetylase (*HDAC7*)^91^, cell cycle regulators (*CDK9*)^92^, and surface antigens (*SLAMF7*)^93,94^ (Figures 6D to 6K). Several of these SE-regulated genes were highly expressed and essential for cell survival in a panel of 20 MM cell lines from the DepMap Portal^95^ (Figure 6L and Table S17).

To assess MM-specificity, we identified genes essential in MM cell lines but not in other cancer cell lines, including *IRF4*^96^, *POU2AF1*^97^, *MYC*^98^, *CCND2*^99^, *EP300*^100^, and *ARID1A*^101^ (Figure S27A and Table S18). We then examined the overlap between these essential genes and the 213 SE-regulated MM genes, identifying 9 SE-regulated, MM-specific essential genes (Figure S27B). This list included well-established targets such as *IRF4*, *IKFZ1* (targeted by IMiDs and cereblon modulators), *MCL1* (under investigation in clinical trials), and *CFLAR* (C-FLIP, associated with EMDR in MM^102^). Other genes, such as *DUSP22* (an immediate neighbor of *IRF4* on chr6p25.3), *ATP6AP1*, *FPGS*, *DPM2*, and *TAFAZZIN*, warrant further investigation as potential therapeutic targets in MM.

To further explore the cause of transcriptional dysregulation in LRMM compared to NDMM, we assessed whether gene expression near SEs and REs (within a 0.5 Mb range) differed significantly between these disease states. We confirmed the anticipated higher expression of SE-regulated genes compared to RE-regulated genes; additionally, both categories of genes were expressed at higher levels in LRMM than in NDMM samples (Figure 6M). We further demonstrated increased expression of key coactivator genes in the LRMM samples compared to the NDMM samples examined by CUT&Tag (Figure 6N). This finding was corroborated by data from the PMRC cohort (Figure 6O). These key coactivators include components of the mediator complex (e.g., MED1), factors interacting with the positive transcription elongation factor P-TEFb and RNA polymerase II (e.g., CDK9, BRD4), histone-modifying enzymes such as acetyltransferase (EP300) and methyltransferase (NSD2), DNA repair proteins (e.g., RAD21), and factors involved in the demarcation of topologically associating domains (TADs)^79^, such as CTCF and YY1^45^. Collectively, our data indicate a combined overall increase in H3K27ac signal and transcriptional machinery activity in LRMM.

To investigate the role of transcription cofactors in relapsed/refractory MM, we used an isogenic proteasome inhibitor (PI)-resistant cell line, RPMI-8226-B25^18,103^ (8226-B25), and the PI-naïve RPMI-8226 (8226) parental cell line as a model of NDMM vs. LRMM. Bulk RNA-Seq confirmed higher expression of transcription machinery and cofactor genes in 8226-B25 compared to 8226 (Figure 6P and Table S19 for gene expression values). Furthermore, silencing of *YY1* (Figure 6Q), a key structural regulator of enhancer-promoter loops^104^, using a lentivirus-mediated, doxycycline-dependent inducible knockdown (shRNA) system, did not significantly decrease cell viability (Figure 6R), but notably sensitized 8226-B25 to both bortezomib and carfilzomib (Figure 6S). In contrast, sensitivity to PIs remained unchanged in the parental cells. These results indicated that YY1’s enhancer-promoting activity potentiates acquired PI resistance, aligning with our observations in patient samples (i.e., LRMM *versus* NDMM). Taken together, these data implicate aberrant SE formation and increased expression of transcription machinery genes and cofactors in driving LRMM. Our findings reveal that distinct mechanisms contribute to increased chromatin accessibility in these cells and highlight SE-regulated, MM-specific lethal genes for further investigation into both MM transformation and multi-therapy resistance.

## Discussion

In this study, we analyzed genomic, transcriptional, and epigenetic data from the PMRC cohort—a comprehensive dataset of approximately 1,000 MM patients with paired clinical and molecular data, spanning premalignant stages to multidrug-relapsed/refractory disease, including longitudinal biopsies from over 100 patients. The PMRC cohort offers key insights into myelomagenesis and therapy resistance while serving as a valuable new resource for the MM research community.

Genomic analysis confirmed well-established MM-associated abnormalities and uncovered novel gain- and loss-of-function driver mutations (e.g., *SUZ12*, *NFKBIA*). At the transcriptional level, pathways related to plasma cell identity, TME dependency, and inflammation were significantly dysregulated in SMM compared to MGUS. Notably, no significant transcriptomic differences were observed between SMM and NDMM, suggesting that their distinction is primarily clinical rather than biological. These findings are particularly relevant as definitions and management strategies for “high-risk” precursor MM continue to evolve (i.e., whether to treat or not to treat). scMultiome analysis revealed a marked decrease in chromatin accessibility at differentially expressed genes between MGUS and SMM, potentially due to an expanded H3K27me3 landscape. Additionally, pseudotime analysis suggested that patient-specific cytogenetic abnormalities serve as initiating events, triggering shared epigenetic and transcriptomic alterations during the MGUS-to-SMM transition—effectively hijacking the natural plasma cell differentiation program.

In contrast, the transition from NDMM to LRMM was marked by the upregulation of transcriptional programs related to proliferation, DNA repair, and metabolism. LRMM differentially expressed genes exhibited increased H3K27ac signal—an indicator of enhancer and super-enhancer activity—compared to NDMM. However, the overall number of super-enhancer-regulated genes remained largely unchanged between these disease states. Additionally, LRMM showed increased expression of transcriptional machinery components, including mediator complex subunits (*MED1*), transcriptional elongation factors (*CDK9*, *BRD4*), histone modifiers (*NSD2*, *EP300*), DNA repair regulators (*RAD21*), and key architectural proteins governing enhancer-promoter interactions^45^ (*CTCF*, *YY1*). Functionally, we validated YY1’s role in drug resistance, demonstrating that its loss re-sensitized bortezomib-resistant MM cell lines to proteasome inhibitors^104,105^.

**Figure 7.**
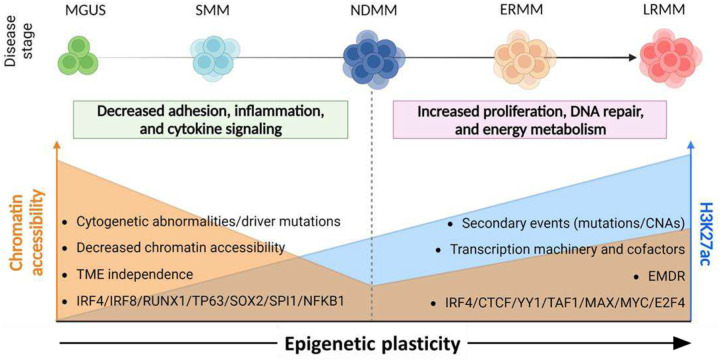
Epigenetic plasticity drives MM evolution. In premalignant stages, cytogenetic abnormalities and driver mutations lead to a significant reduction in chromatin accessibility, driven by H3K27me3 deposition and the activity of the polycomb repressive complex 2 (PRC2). This results in altered expression of key transcription factors, including *IRF4*, *IRF8*, *RUNX1*, *TP63*, and *SOX2*, ultimately reducing tumor cell dependence on the tumor microenvironment (TME). As paracrine signaling-independent clones expand, MM emerges. Subsequent therapeutic interventions and clonal evolution select for clones with increased chromatin accessibility and histone acetylation. These epigenetic changes enhance the binding of transcription factors such as CTCF, YY1, TAF1, and MYC, driving the upregulation of transcriptional machinery and cofactors. This, in turn, promotes transcriptional programs associated with proliferation, DNA repair, and metabolism, culminating in the development of multidrug resistance. (Created with BioRender.com).

Our study introduces a transformative conceptual framework for understanding MM onset and therapy resistance (Figure 7). We propose a paradigm shift in which the genomic events characteristic of MM do not arise as direct adaptations to the TME or therapeutic pressure. Instead, these events release epigenetic “locks” that normally suppress transcriptional networks involved in TME dependency, paracrine signaling, apoptosis, and plasma cell identity. One example is the nuclear receptor-binding SET domain protein 2 (NSD2, or MMSET), a histone methyltransferase and key oncogenic driver in the t(4;14) translocation^106^. NSD2 enhances H3K36me2 formation, promotes a more open chromatin structure, and reduces H3K27me3 levels^107^. Our data confirm that t(4;14) disrupts the balance between *NSD2* and *EZH2* expression (Figure S28), contributing to increased chromatin accessibility of genes overexpressed in refractory disease.

Intratumoral competition within the bone marrow niche, compounded by selective pressure from multi-therapy treatments, favors clones with heightened epigenetic plasticity. This plasticity enables the activation of latent biological programs—such as those governing DNA repair, proliferation, and metabolism—while repressing plasma cell-associated transcriptional networks typically targeted by standard MM therapies (e.g., corticosteroids, IMIDs, proteasome inhibitors) and immunotherapies (e.g., BCMA-directed therapies). These adaptations collectively enhance cellular fitness, facilitate dissemination, and drive repeated cycles of immune evasion and therapeutic resistance. We term this process “cellular anamnesis”, drawing from Plato’s concept of *anamnesis*, in which knowledge is not acquired but “recollected” from past lives. Through epigenetic plasticity, MM cells “reawaken” transcriptional programs silenced (“forgotten”) during plasma cell differentiation. This model provides a unifying framework for several key observations in MM, including: (i) the absence of gatekeeping genomic drivers explaining progression from premalignant states (e.g., *BCR::ABL1* in CML); (ii) pronounced interpatient genomic, transcriptomic and epigenetic heterogeneity, particularly in advanced, multidrug-resistant disease; (iii) the failure of in vitro-selected drug resistance models to fully recapitulate clinical resistance (i.e., the selection of specialized phenotypes in vitro vs. plasticity/multi-drug resistance in patients^108^); (iv) increasingly rapid and aggressive relapses following each treatment failure; (v) the lack of reliable genomic biomarkers for therapy response; and (vi) the convergence of distinct transcriptional programs underlying resistance to mechanistically diverse therapies^63^.

As epigenetic plasticity stands as the root cause behind both MM carcinogenesis and the emergence of therapy resistance, targeting this molecular process could have significant implications for both tumor prevention and treatment^109^. The FDA-approved EZH2 inhibitor tazemetostat, currently indicated for relapsed/refractory follicular lymphoma^110^, is being evaluated for its efficacy in LRMM as part of the CA057–003 phase 1/2 trial (NCT05372354) in combination with mezigdomide (a CELMoD agent) and dexamethasone^111^. The critical role of PRC2 in malignant transformation, as identified in our study, leads us to hypothesize that tazemetostat may be particularly effective in high-risk SMM patients by preventing progression to active MM. Furthermore, the dysregulated acetylation of histones (H3K27ac) could represent a therapeutic vulnerability, potentially targetable by histone/lysine acetyltransferase (HAT/KAT) inhibitors, particularly in refractory/relapsedMM^109,112–114^. The CREBBP/EP300 bromodomain inhibitor inobrodib, currently being tested in combination with pomalidomide and dexamethasone in a phase 1/2a clinical trial for MM (NCT04068597), has shown promising activity, with an overall response rate of 67% reported so far^115^. Notably, CBP/p300 inhibitors induce MM cell death by suppressing *IRF4* expression^116,117^, which we identified as the most differentially lethal gene for MM (Figure S27A).

In conclusion, our study provides a comprehensive framework for understanding the epigenetic and transcriptional reprogramming driving the progression of MM, from its premalignant stages to the development of multidrug resistance in advanced disease. Our findings highlight the critical need to integrate therapies targeting epigenetic plasticity into MM treatment regimens. By inhibiting the expression of transcriptional programs that promote survival and downregulate plasma cell markers, such modulators could not only extend the therapeutic window of potent, albeit toxic, therapies, but also re-sensitize MM cells to existing chemo- and immunotherapeutic treatments. Future studies should focus on identifying the biological and clinical contexts in which epigenetic plasticity modulators most effectively block cellular anamnesis, thereby preventing malignant transformation and the acquisition of therapy resistance.

## Methods

### Human subjects

Peripheral blood and bone marrow (BM) aspirates were collected from patients with diagnoses within the MM spectrum, treated at Moffitt Cancer Center from 2011 to 2019. All patients provided signed informed consent to participate in the *Total Cancer Care*^20^ protocol (TCC^™^, protocol MCC14690/Advarra IRB Pro00014441), which is part of the ORIEN/Avatar program. Samples and data were accessed from TCC for this research under protocol MCC18608. All work was conducted at the H. Lee Moffitt Cancer Center and Research Institute, as approved by the Institutional Review Board (IRB). Patient samples were used in accordance with the principles outlined in the Declaration of Helsinki, International Ethical Guidelines for Biomedical Research Involving Human Subjects (CIOMS), Belmont Report, and the U.S. Common Rule. Demographic details of the patients are provided in Table S1.

### CD138+ cells isolation and purity assessment

To maximize the yield of MM cells, 20 mL of BM aspirate were obtained using a fenestrated Jamshidi needle, with repositioning throughout the procedure. BM mononuclear cells (BMMCs) were isolated using a Ficoll gradient from fresh BM aspirates, washed, and resuspended in 30 mL of PBS 1X. Cells were counted using a Cellometer Spectrum 5 (Nexcelom Bioscience) with AO/PI staining, allowing for the calculation of the total live cell count. We targeted 100,000 cells to prepare a pre-cytospin slide to assess PC percentage. The slides were stained with Wright’s stain and examined under a microscope to determine the PC percentage. Next, BMMCs were enriched for CD138 expression using antibody-conjugated magnetic beads (Miltenyi Biotec Cat# 130-051-301, RRID:AB_3665843). Cytospin slides of the CD138+ cells were prepared to evaluate PC enrichment post-isolation. The post-isolation purity of the examined samples was greater than 95.3% (IC: 88.1% - 100%), confirming their high purity. Additionally, the purity of our samples was assessed by querying the RNA-Seq data from the DepMap portal (RRID:SCR_017655), identifying 20 genes selectively expressed in MM or in all non-hematological malignancies (i.e., other cancers, OC). These genes are henceforth referred to as MM genes and OC genes, respectively (Figure S1A). We observed consistently higher expression of MM genes and lower expression of OC genes across all patient samples, regardless of their disease state, indicating that the samples were highly enriched for PCs (Figure S1B). This was statistically confirmed by comparing the mean log_2_(FPKM + 10^−3^) values of MM and OC genes in non-MM samples to those in each disease state. MM genes consistently showed higher expression in all disease states compared to non-MM samples, whereas the expression of OC genes did not exhibit any statistically significant difference (Figure S1C). Further, we assessed sample purity of the 18 samples analyzed using scMultiome by determining the proportion of cells per sample across UMAP plot clusters based on the expression of specific gene markers (*IL1B* for macrophages, *BCL11B* for T cell precursors, *NKG7* for NK and CD8^+^ T cells, *SLC4A1* for mature erythroid cell, *AFF3* and *EBF1* for immature B cells and *IRF4* for PCs; Figure S10). Compared to bone marrow aspirates from healthy donors, which contain a significant proportion of non-PC cells (ranging from 52% to 75%), MGUS samples showed 25% or fewer of these cells, SMM samples 15% or fewer, and active disease samples only 4% or fewer (Figure 5C), confirming the high purity of the disease samples.

### Whole exome sequencing (WES)

Following CD138+ enrichment, vials containing 1.0 × 10^6 viably frozen CD138+ cells were shipped for WES as part of the ORIEN/Avatar program.

*Nucleic Acid Extraction:* For frozen tissue DNA extraction, Qiagen QIA Symphony DNA purification was used, resulting in an average insert size of 213 bp.

*DNA Sequencing:* Preparation of Whole Exome Sequencing (WES) libraries involved hybrid capture using an enhanced IDT WES and Nimblegen SeqCap EZ kits (38.7 Mb) with additional custom designed probes for double coverage of 440 cancer genes. Library hybridization was performed at either single or 8-plex and sequenced on an Illumina NovaSeq 6000 instrument, generating 100 bp paired reads. WES was performed on tumor/normal matched samples with the normal covered at 100X and the tumor covered at 300X (additional 440 cancer genes covered at 600X) depth. Both tumor/normal concordance and gender identity QC checks were performed. Minimum threshold for hybrid selection was >80% of bases with >20X fold coverage; ORIEN/Avatar WES libraries typically meet or exceed 90% of bases with >50X fold coverage for tumor and 90% of bases with >30X fold coverage for normal samples.

### Mutation calling

Individual VCF files were converted to tab separated format using the software vcf2tsv (https://github.com/sigven/vcf2tsv version=0.3.4). Only gene mutation records with column value “PASS” for field “FILTER” and “exonic status” as “exonic” and type “protein coding” were considered. All files were merged and formatted according to minimum requirements and processed using the R package *maftools* (RRID:SCR_024519, v.2.4.12). Mutational summaries for non-synonymous mutations were created using *maftools* functions *oncoplot* and *plotmafSummary*.

### dN/dS calculation

We calculated dN/dS ratios using the R library dndSCV (RRID:SCR_017093, v.0.0.1.0)^27^ using as genomic reference RefCDS_human_GRCh38.p12.rda and default parameters of function *dndscv*. Significant dN/dS values were filtered (sel_cv$qglobal_cv<0.1).

### RNA sequencing (RNA-Seq)

Following CD138+ enrichment, vials containing 1.0 × 10^6 viably frozen CD138+ cells were shipped for RNA-Seq analysis as part of the ORIEN/Avatar program.

*Nucleic Acid Extraction:* For frozen tissue RNA extraction, the Qiagen RNeasy Plus Mini Kit was employed, generating an average insert size of 216 bp.

*RNA Sequencing*: was performed using the Illumina TruSeq RNA Exome with single library hybridization, cDNA synthesis, library preparation, sequencing (at either 100 or 150 bp paired reads) to a coverage of 100M total reads / 50M paired reads. RNA-Seq Tumor Pipeline Analysis was processed according to the workflow outlined below using GRCh38/hg38 human genome reference sequencing and GENCODE (RRID:SCR_014966, v.32).

*Adapter trimming:* Adapter sequences were trimmed from the raw tumor sequencing FASTQ files. Adapter-trimming via *k*-mer matching was performed along with quality-trimming and filtering, contaminant-filtering, sequence masking, GC-filtering, length filtering and entropy-filtering. The trimmed FASTQ file was used as input to the read alignment process.

*Read Alignment:* The tumor adapter-trimmed FASTQ files were aligned to the human genome reference (GRCh38/hg38) on GENCODE (RRID:SCR_014966, v.32) using STAR (RRID:SCR_004463).

*RNA expression:* RNA expression values were calculated and reported using estimated mapped reads, Fragments Per Kilobase of transcript per Million mapped reads (FPKM), and Transcripts Per Million mapped reads (TPM) at both transcript level and gene level based on transcriptome alignment generated by STAR (RRID:SCR_004463).

*Gene expression data download and normalization*: Gene expression data were obtained from DNAnexus (RRID:SCR_011884) files containing FPKM and TPM values for 59,368 records. Of these, 19,933 were protein-coding genes and selected for further analysis, while the remaining records were excluded. For each gene/sample, log2(FPKM + 10−3) values were calculated. Genes with identical first and third quartile values (i.e., expressed in less than 25% of samples) were removed from further analysis. The resulting dataset of 16,738 genes was z-normalized across all samples using MATLAB’s *normalize* function.

### Differential gene expression and enrichment analysis across the MM spectrum

To identify genes differentially expressed across MM spectrum, samples were grouped according to disease state and Student’s t-tests were calculated for each gene/transition (e.g., *MYC* between SMM and NDMM samples) and multiple test correction was performed using False Discovery Rate method (two-stage step-up method of Benjamini, Krieger and Yekutieli, *q*=0.01) calculated using GraphPad Prism (RRID:SCR_002798, v.10). Enrichment analyses for transcription factors and histone modifications were conducted using the metadata search feature Enrichr (RRID:SCR_001575) using as input the list of genes differentially expressed across the disease states of interest. Under the tab “Transcription”, the enrichment results for the following databases were considered: “ENCODE and ChEA Consensus” for putative transcription factors and “ENCODE Histone Modifications 2015” for histone modifications. Enrichment analyses for differentially expressed KEGG Pathways and Cancer Hallmarks across disease states were conducted by Gene Set Enrichment Analysis (GSEA) software^28^ (RRID:SCR_003199, v. 4.3.2). Analyses were made using preranked gene lists based on the difference between mean z-scores for the disease states being compared and adopting the following parameters: Number of permutations = 1000; Collapse/remap to gene symbols = no collapse; Chip platform = Human_Ensembl_Gene_ID_MSigDB.v2023.2.Hs.chip; Enrichment statistic = classic; Maximum/minimum size of data sets = 500/15; Collpasing mode for probe sets ≥ 1 gene = Abs_max_of_probes; Normalization mode = meandiv.

### Principal Component Analysis (PCA) of biological pathways

To characterize the transcriptional state of the samples individually, we conducted single sample gene set enrichment analysis (ssGSEA^28^) of Cancer Hallmark pathways using the script *ssgsea-gui.r* from the SSGSEA 2.0 library (github.com/broadinstitute/ssGSEA2.0) for R (v.4.0.2). Normalized enrichment score (NES) of gene sets with *p*-value>0.01 were set to zero, indicating no enrichment. Principal component analysis based on Cancer Hallmarks gene sets’ ssGSEA scores was conducted using MATLAB’s *pca* function. Bidimensional plots of principal components for MM samples, as well as feature loadings, were generated using the *biplotG* function (iaisidro.wordpress.com). A similar analysis was conducted for the CoMMpass cohort (i.e., ssGSEA scores were calculated for each of the 50 Cancer Hallmarks gene sets using bulk RNA sequencing data from 704 NDMM patient samples).

### Cox Proportional Hazard (CoxPH) models and survival curves

CoxPH models were made in R using the *coxph* function from package *survival* (RRID:SCR_021137) to estimate hazard ratios of the transcriptome (PC1>0), as well as of the presence of driver mutations, cytogenetic abnormalities, and demographic features. These additional variables were incorporated into univariate Cox Proportional Hazard models when at least three patient samples exhibited a specific trait (n≥3). Multivariate CoxPH models were made with the significant (p<0.05) variables in the univariate analysis, after removal of composed variables (ISS, R-ISS, Double/Triple hit, Mayo Risk), as well as superclusters **α** and **β** (to avoid overlap with the transcriptional information conveyed by feature “PC1>0”). Forest plots and Kaplan-Meier curves were made using GraphPad Prism (RRID:SCR_002798, v.10). Differences between survival functions were tested using the Log-rank (Mantel-Cox) test.

### Transcriptional topology map of MM

The t-distributed Stochastic Neighbor Embedding (t-SNE, RRID:SCR_024305) dimensionality reduction analysis was performed with MATLAB’s (RRID:SCR_001622) function *tsne* and used Z-normalized expression of 16,738 genes across 844 MM samples with RNA-Seq data available as input. Gene cluster segmentation was performed using MATLAB’s function *fcm*, which implements the algorithm Fuzzy c-means^64^. Correlation analysis between gene clusters and cancer hallmarks was calculated based on Pearson correlation between ssGSEA of gene clusters and cancer hallmarks for all samples with RNA-Seq data; unsupervised clustering analysis was performed using MATLAB’s function *clustergram*. Chromosomal location of genes was obtained from GeneHancer (RRID:SCR_023953) database. KEGG Pathaway Database (RRID:SCR_018145 and Cancer Hallmark gene sets were obtained from Molecular Signatures Database (MsigDB, RRID:SCR_016863). Differential expression of superclusters **α** and **β** was calculated based on unpaired Student’s t-tests of ssGSEA of either gene set across MM spectrum. To identify the putative role of histone modifications and epigenetic regulation on determining the transcriptional topology of MM, we have conducted enrichment analysis of each of the 500 gene clusters from the t-SNE plot and “ENCODE Histone Modifications 2015” database using Enrichr (RRID:SCR_001575). Only entries with enrichment adjusted *p*-value lower than 0.01 were considered in this analysis. All genes pertaining to clusters enriched for either group of histone modifications were coalesced into two gene sets, named H3K27me3 or H3K27ac, and ssGSEA was calculated for all samples, which were in turn grouped by disease state. Student *t*-tests were performed using GraphPad Prism (RRID:SCR_002798, v.10) to assess difference of ssGSEA NES score across MM spectrum.

### Single-cell paired ATAC/RNA-sequencing (scMultiome)

We developed an optimized protocol for the high-quality isolation of viably frozen bone marrow (BM)-derived CD138+-selected cells. The sample processing and sequencing steps were performed as follows:

*Thawing and Resuspension*: Cells were thawed and resuspended dropwise in warm culture media (RPMI-1640 + 10% fetal bovine serum). Sequential additions of media were made to double the volume already present in the cell suspension, up to a total volume of 32 mL. Each addition was spaced 1 minute apart, following the manufacturer’s instructions (10X Genomics, Protocol CG000365).

*Centrifugation and Viability Assessment*: The cells were centrifuged at 500 *g* for 5 minutes at room temperature. After centrifugation, the cell pellet was resuspended in 1 mL of warm media and transferred to a 1.5 mL Eppendorf vial. Viability was assessed using trypan blue staining (Invitrogen Countess II Automated Cell Counter, RRID:SCR_025370).

*Additional Centrifugation and Lysis*: The cells were centrifuged again at 500 g for 5 minutes at 4°C using a mini centrifuge. The supernatant was discarded, and 200 μL of cold 0.1X lysis buffer (containing digitonin (5% Digitonin, Invitrogen, Cat# BN2006, Lot# 2487054) at a concentration of 0.15X) was added to the cell pellet. Real-time viability monitoring was performed every 3 minutes using the Countess system. Lysis was allowed to proceed until viability dropped below 5% (between 6–10 minutes of lysis).

*Washing Steps*: Once viability reached the specified threshold, 1 mL of cold wash buffer was added to the cells. The cells were then centrifuged at 500 *g* for 5 minutes at 4°C, and the supernatant was removed. This washing step was repeated three times in total.

*Nuclei Resuspension and Preparation*: The isolated nuclei were resuspended in 125 μL of diluted nuclei buffer (prepared according to the manufacturer’s instructions, 10X Genomics, Protocol CG000365), and passed through a 40 μm Flowmi Cell Strainer (Scienceware Flowmi Cell Strainers, SP Bel-Art, Cat# H13680–0040). Viability was assessed by trypan blue staining, and the nuclei were promptly processed for transposition, following the manufacturer’s instructions (10X Genomics, Protocol CG000338).

*Nuclei encapsulation*: Following a transposition reaction, the transposed nuclei suspension was immediately loaded onto the 10X Genomics Chromium Controller Genetic Analyzer (RRID:SCR_019326) using a 10X Chromium Next GEM Chip J Single Cell Kit. Briefly, the transposed nuclei, reagents, and 10X Genomics gel beads were encapsulated into individual nanoliter-sized Gelbeads in Emulsion (GEMs) and then linear amplification and barcoding were performed inside each droplet. The reaction was then cleaned up and DNA libraries were completed in a single bulk reaction using the 10X Genomics Chromium NextGEM Single Cell ATAC Reagent Kit v1.1.

*Sequencing*: 25,000 and 50,000 pairs of sequencing reads (ATAC and RNA, respectively) per cell were generated on the Illumina NextSeq 500 instrument according to the manufacturer’s protocol (Illumina).

*Analysis:* Demultiplexing, barcode processing, alignment, and peak counting were performed using 10X Genomics Cell Ranger (RRID:SCR_017344, v3.1.0). Visualization of cell clustering, gene accessibility, and gene expression was performed using Loupe Browser (RRID:SCR_018555, v.8).

*Enrichment analysis from the scRNA-Seq compartment:* ssGSEA for the enrichment of transcription factor targets and Cancer Hallmarks was conducted across the individual cells using the script *ssgsea-gui.r* from the SSGSEA 2.0 library (github.com/broadinstitute/ssGSEA2.0) for R (v.4.0.2). ssGSEA NES were averaged across cells within each UMAP cluster. Row-scaled single-linkage hierarchical clustering (correlation-based distance) was performed in R using the *heatmap.2* function from the *gplots* library.

*Enrichment analysis from the scATAC-Seq compartment:* Loupe Browser (RRID:SCR_018555) was utilized to identify differentially accessible genes (based on log2 fold change) across clusters being compared (clusters C2 to C13, relative to cluster C1) by employing the “locally distinguishable” query for “promoterSum”. GSEA analyses for Cancer Hallmarks were performed on pre-ranked gene lists. Hallmarks enriched in at least one comparison were subjected to a single-linkage hierarchical clustering analysis using a Euclidean distance function customized to ignore “NaN” (i.e., situations in which a particular Hallmark was not enriched). This analysis was conducted in R using the *heatmap.2* function from the *gplots* library.

*Inferring copy number alterations:* We have calculated gene copy number at single level from scRNA-Seq using inferCNV (RRID:SCR_021140, v.1.20.0) and from scATAC-Seq using epiAneufinder, (RRID:SCR_026269, v.0.1.0) with default parameters.

*Determining putative pioneer transcription factors in MM:* We have used ArchR (RRID:SCR_020982, v.1.0.2) to identify TFs whose expression (from scRNA-Seq) correlated (Pearson r) with chromatin accessibility (from scATAC-Seq) of their binding motifs using function *correlateMatrices*. These TFs were labeled as “putative” pioneer transcription factors, as functional validation would be required to definitively establish their roles as pioneer TFs.

### CUT&Tag assay

Frozen cell pellets (human myeloma, purified primary cells, CD138+) of 4 NDMM and 4 LRMM samples were analyzed. Briefly, cells were incubated overnight with Concanavalin A beads and 1.3 μl of the primary anti-H3K27ac antibody per reaction (Active Motif Cat# 39133, RRID:AB_2561016). After incubation with the secondary anti-rabbit antibody (1:100), cells were washed and tagmentation was performed at 37 ℃ using protein-A-Tn5. Tagmentation was halted by the addition of EDTA, SDS and proteinase K at 55℃, after which DNA extraction and ethanol purification was performed, followed by PCR amplification and barcoding. Following SPRI bead cleanup (Beckman Coulter), the resulting DNA libraries were quantified and sequenced on Illumina’s NextSeq 550 (8 million reads, 38 paired end). Reads were aligned using the BWA algorithm^118^ (mem mode; default settings). Duplicate reads were removed, and only reads that mapped uniquely (mapping quality >=1) and as matched pairs were used for further analysis. Alignments were extended in silico at their 3’-ends to a length of 200 bp and assigned to 32-nt bins along the genome. The resulting histograms (genomic “signal maps”) were stored in bigWig files. Peaks were identified using the MACS 2.1.0 algorithm at a cutoff of *p*-value 1e-7, without control file, and with the –nomodel option. Peaks that were on the ENCODE (RRID:SCR_006793) blacklist of known false ChIP-Seq peaks were removed. Signal maps and peak locations were used as input data to Active Motif’s proprietary analysis program, which creates Excel tables containing detailed information on sample comparison, peak metrics, peak locations, and gene annotations. For differential analysis, reads were counted in all merged peak regions using Subread, and the replicates for each condition were compared using DESeq2^119^. Other key software used: bcl2fastq (RRID:SCR_015058, v2.20) (processing of Illumina base-call data and demultiplexing); SAMTOOLS (RRID:SCR_002105, v0.1.19) (processing of BAM files); BEDTools (RRID:SCR_006646, v2.25.0) (processing of BED files); wigToBigWig (v4) (generation of bigWIG files); Subread (RRID:SCR_009803, v1.5.2) (counting of reads in BAM files for DESeq2).

*Super enhancer calling:* We used ROSE^120,121^ (RRID:SCR_017390) to identify active super enhancers (SEs) in eight MM samples with H3K27ac CUT&Tag data, applying default parameters except for the stitching window, which was adjusted from 12.5 kb to 15 kb. Genes annotated in TxDb.Hsapiens.UCSC.hg38.knownGene were considered under the control of these SEs if an interval spanning 50 kb upstream and 5 kb downstream of their transcription start sites (TSS) overlapped with the identified SEs.

*DepMap Portal data*: Gene expression data (log2 TPM) and cell dependency data (CRISPR knockout) were downloaded from the Cancer Dependency Map Portal (RRID:SCR_017655) for all cell lines.

### Generation of the PI-resistant 8226-B25 cell line

RPMI 8226-B25 (“8226-B25”, RRID:CVCL_E5J8) cells were developed in-house from the RPMI 8226 (“8226”, ATTC Cat# CCL-155, RRID:CVCL_0014) cell line by chronic exposure to bortezomib with incremental dose increases up to 25 nM over several months in monoculture^18,103^.

### Gene expression analysis in 8226 and 8226-B25 cells

All samples were prepared in biological triplicates, with each sample containing 1 × 10⁶ cells. Total RNA was isolated using the RNeasy Plus Mini Kit (QIAGEN, Cat# 74134), and libraries were prepared with the TruSeq Stranded mRNA Library Prep Kit (Illumina, Cat# RS-122–2101/2) following the manufacturer’s instructions. RNA sequencing was conducted on a HiSeq 2500v4 high-output platform, generating 50-bp single-end reads. Gene expression was quantified using Kallisto^122^ with RefSeq^123^ annotation. Transcripts per million (TPM) values were calculated, and genes were classified as expressed if TPM>1.

### Gene knockdown

Sense and antisense primers corresponding to the *YY1* 3-prime untranslated region (5’-ggcagggatattcaccattatcgtttcaga-3’) were obtained from Millipore and cloned to the pLKO-Tet-On lentiviral vector (RRID:Addgene_21916) as previously described^124^. Lentiviral packaging of the plasmid was performed in HEK293T cells (ATTC Cat# CRL-3216, RRID:CVCL_0063) using lipofection reagent (FuGENE HD Transfection Reagent, Promega, Cat# E2311) and Lentiviral Packaging Mix (MISSION Lentiviral Packaging Mix, Sigma-Aldrich, Cat# SHP001). Viral supernatant was collected and filtered (Millex-HA, Millipore, Cat# SLHAM33SS). 8226 (ATTC Cat# CCL-155, RRID:CVCL_0014) and 8226-B25 cell lines were spin-infected with the viral supernatant in the presence of 8 μg/mL polybrene (Polybrene Transfection Reagent, Millipore, Cat# TR-1003-G) and selected with 400 μg/mL G418 (Geneticin, InvivoGen, Cat# ant-gn-5).

### Western blot of YY1

Cell extract, preparation and immunoblotting were performed as previously described^125^, using the following antibodies: YY1 (Santa Cruz Biotechnology Cat# sc-7341, RRID:AB_2257497), alpha tubulin (Santa Cruz Biotechnology Cat# sc-32293, RRID:AB_628412), GAPDH (Abcam Cat# ab181602, RRID:AB_2630358), and horseradish peroxidase-conjugated secondary antibodies (Jackson ImmunoResearch Cat# 115-035-174, RRID:AB_2338512; and Cat# 211-032-171, RRID:AB_2339149).

### Viability assay

RPMI 8226 (ATCC Cat# CCL-155, RRID:CVCL_0014) and 8226-B25 cells (RRID:CVCL_E5J8) were seeded into 384-well plates (384-well Plate TC Surface, Thermo Scientific, Cat# 164688) at a density of 3 × 103 cells per well in 90 μL of medium. After a 48-hour induction with 0.5 μg/mL doxycycline (Doxycycline Hyclate, Sigma-Aldrich, Cat# D9891), cells were treated with the drug for 72 hours. Cell viability was assessed by measuring metabolic activity using the Cell Counting Kit-8 (CCK-8, Dojindo Laboratories, Cat# CK04) following the manufacturer’s protocol.

## Supplementary Material

1

## Data Availability

The molecular data used in this research was generated through private funding by Aster Insights (www.asterinsights.com) in collaboration with the Oncology Research Information Exchange Network (ORIEN, www.oriencancer.org). Requests for access to the raw data (FASTQ/BAM) used in this study can be submitted here at https://researchdatarequest.orienavatar.com/. A curated MAF file, containing information on disease state, UAMS classification, survival, and cytogenetics data, along with a gene expression FPKM file, is available in dbGaP under accession number **phs003892**. Raw and processed files have been deposited in dbGaP and are accessible under accession number **phs003892**.
