# Supplementary material for: Epigenetic Plasticity Drives Carcinogenesis and Multi-Therapy Resistance in Multiple Myeloma
Source: Res Sq. 2025 Apr 15:rs.3.rs-6306816. Preprint. [Version 1] doi: 10.21203/rs.3.rs-6306816/v1 (PMC12048002; doi:10.21203/rs.3.rs-6306816/v1)
Supplement: 1 [file NIHPPRS6306816V1-supplement-1.pdf]

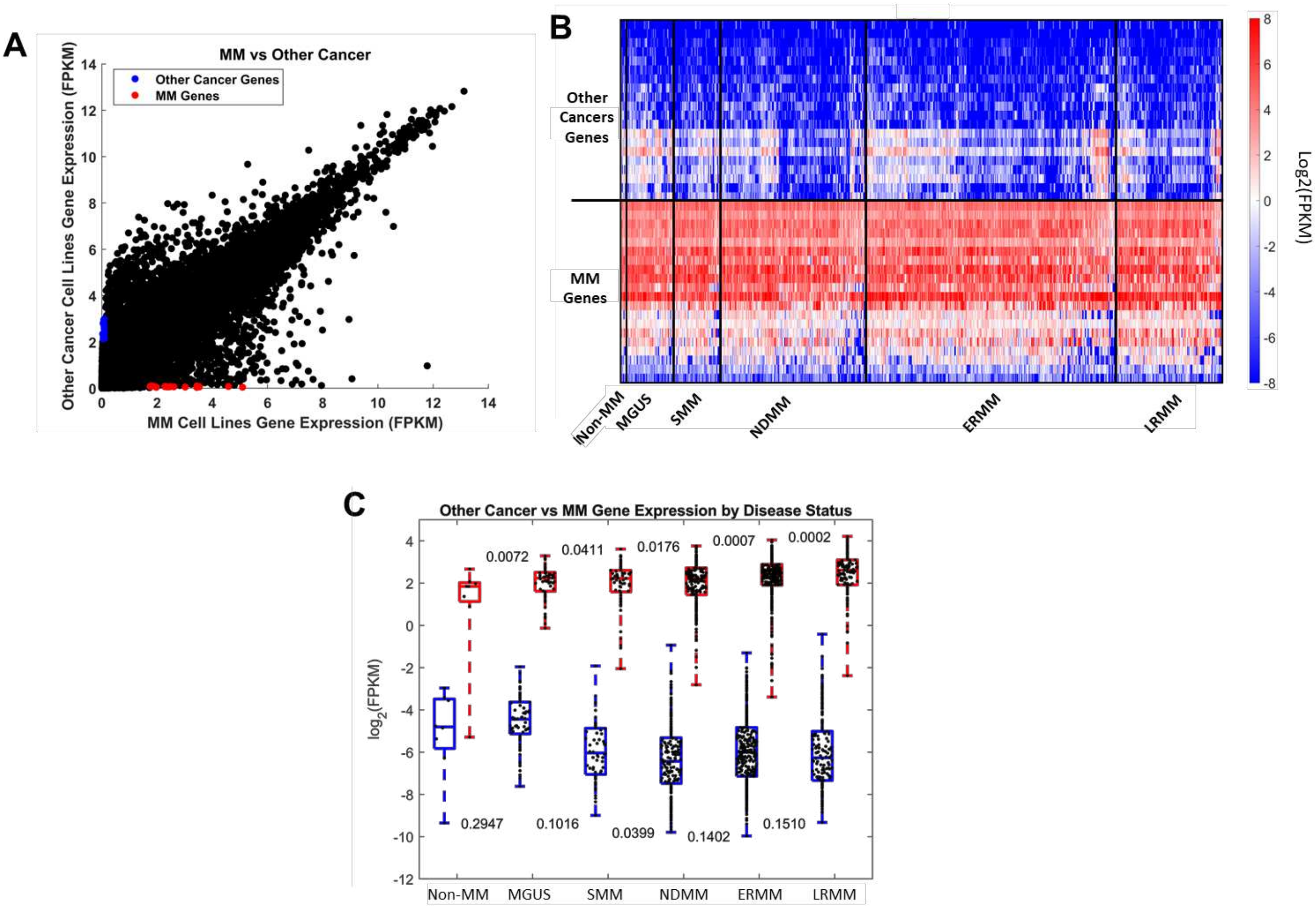

Figure S1. Enrichment for CD138+ cells in the PMRC cohort. A, The mean expression of genes across MM cell lines and non-hematological malignancies cell lines (from DepMap Portal Database) was used to identify the 20 most selectively expressed genes in either MM (red) or Other Cancers (blue). **B**, The expression (log2(FPKM+10^{-3})) pattern of the genes identified in (**A**) across our cohort's samples indicate their high purity. **C**, Mean log2(FPKM +10^{-3}) of the 20 MM (red) or Other Cancers (blue) genes identified in (**B**) across the disease states; numbers in the top refer to P-values from unpaired t-tests between the expression of the genes in non-MM samples and each of the other disease states, indicating increased expression of MM genes (but not other cancers genes) in samples from MM disease stages.

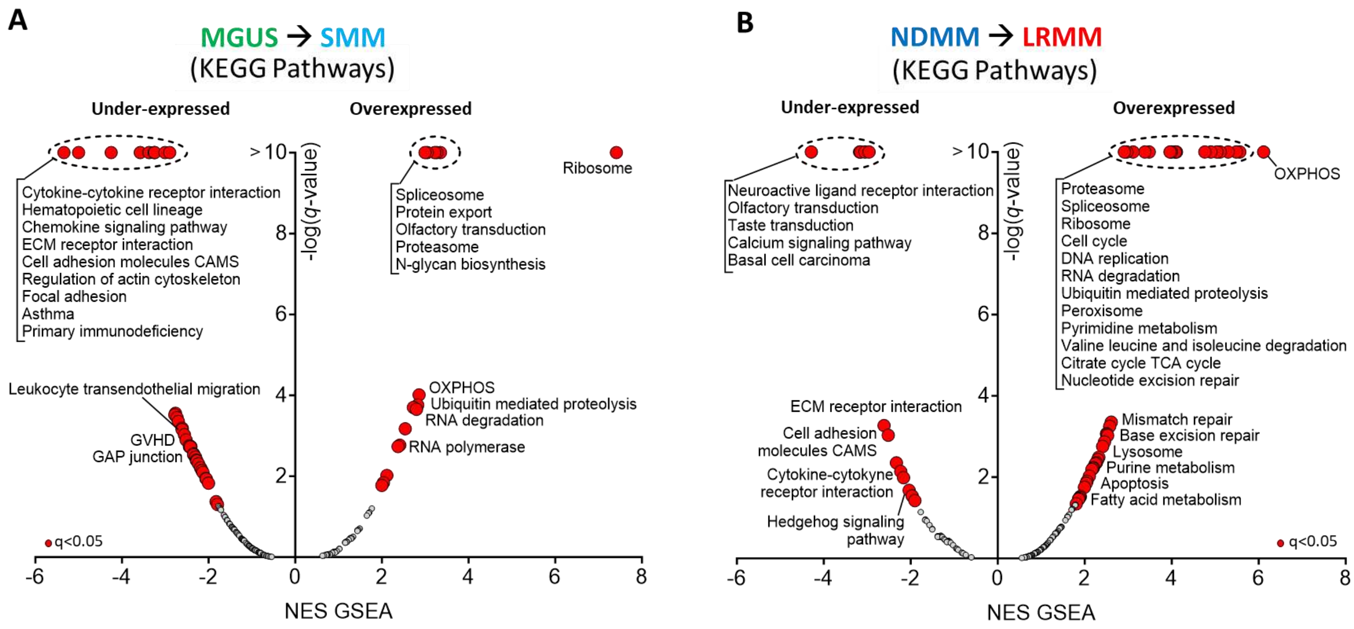

Figure S2. KEGG Pathways' differential expression across disease progression. A, Group-wise comparison of GSEA normalized enrichment scores (NES) revealed KEGG Pathways differentially expressed in the MGUS-to-SMM transition and in the (**B**) NDMM-to-LRMM comparison. Pathways of interest are labeled and highlighted in red.

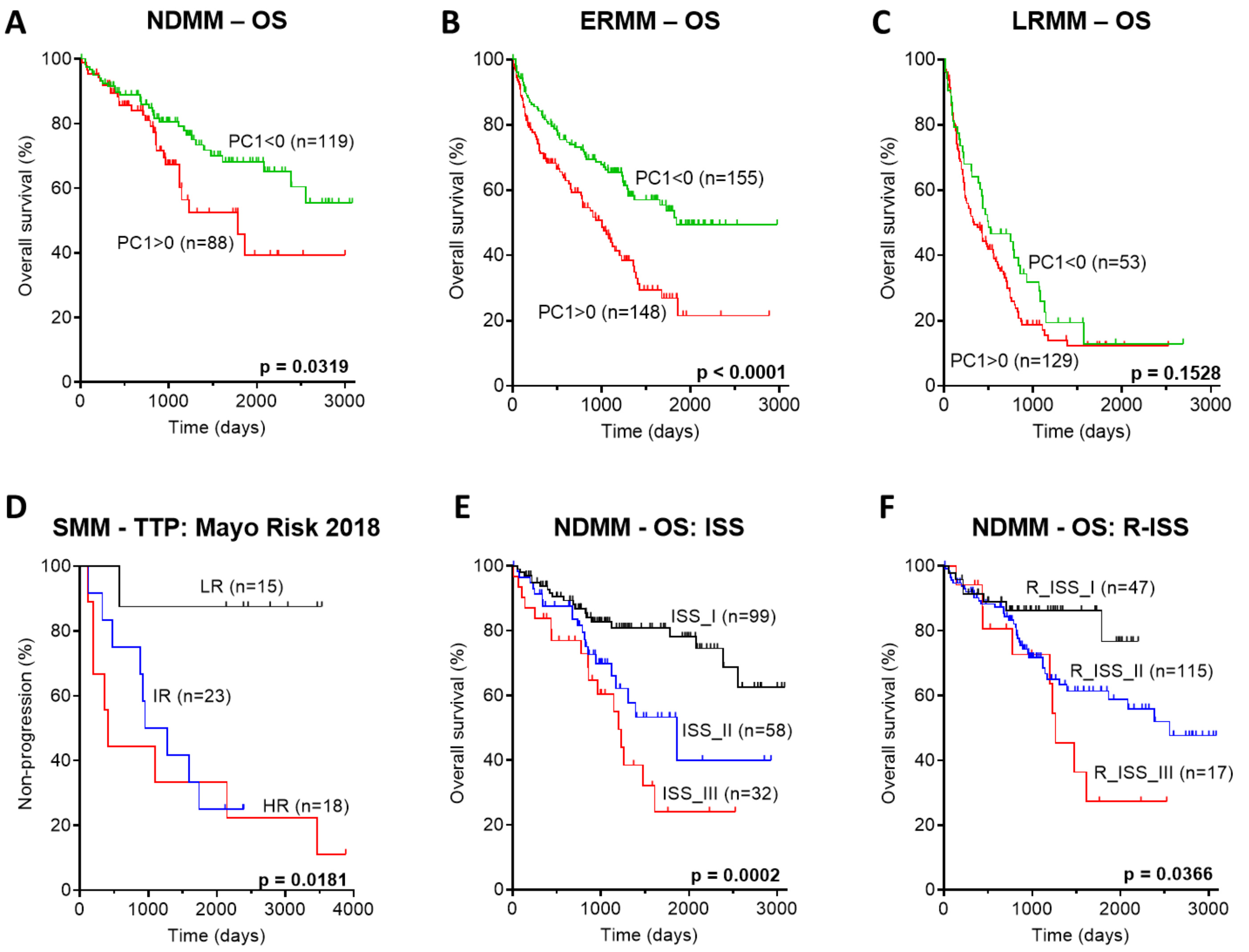

Figure S3. PC1 association with outcome in Active MM, and survival curves for SMM (Mayo Risk 2018) and NDMM (ISS/R-ISS indices). (**A–C**) Survival curves show that PC1 is associated with prognosis in (**A**) NDMM, (**B**) ERMM, nut not (**C**) LRMM. **D**, Time-to-progression for SMM patients according to Mayo's Risk factor 2018. **E**, OS of NDMM according to ISS and (**F**) R-ISS. *P*-values from Log-rank (Mantel-Cox) test.

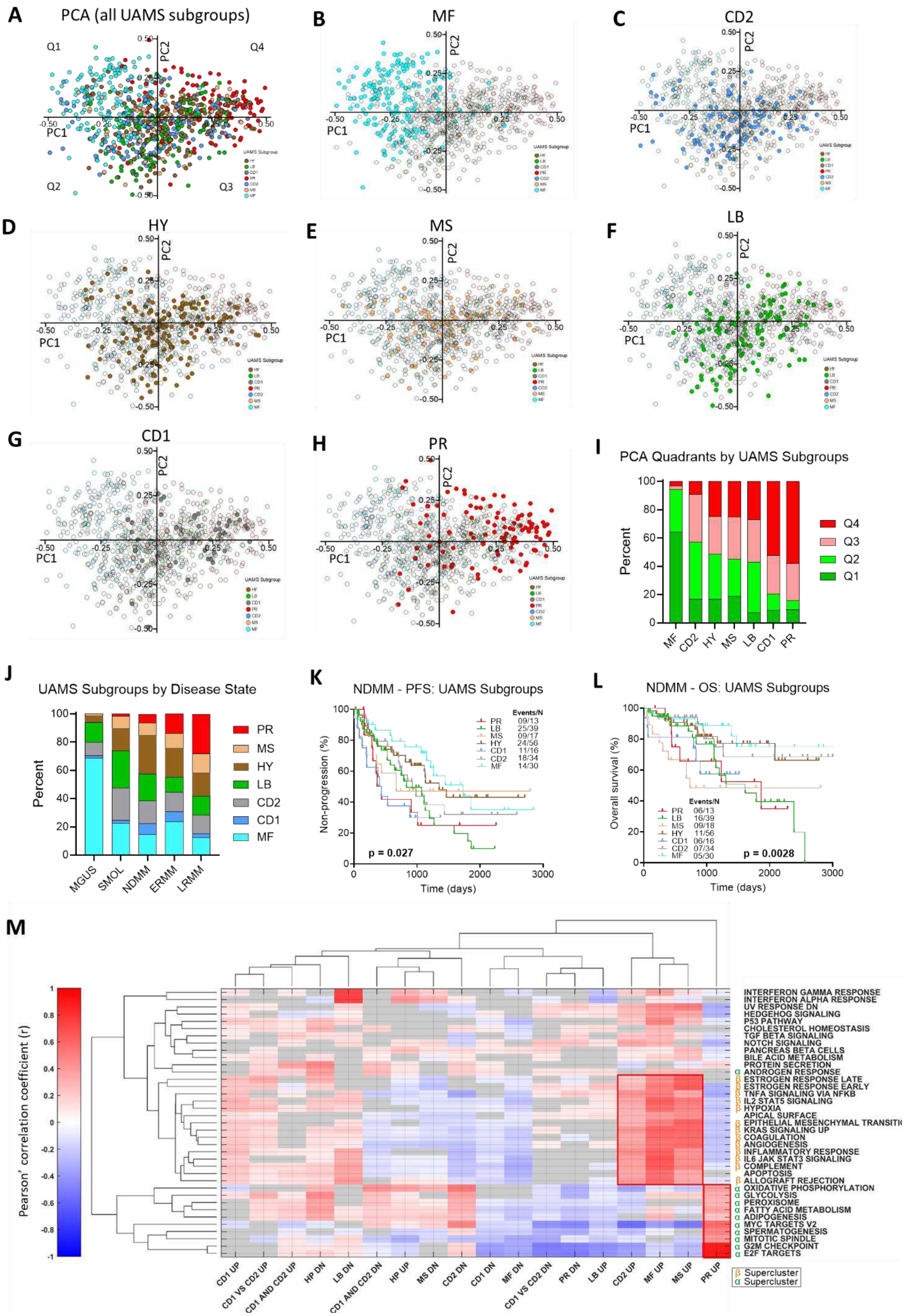
A
PCA (all UAMS subgroups)
Q1
Q4
Q2
Q3
PC1
PC2
B
MF
C
CD2
D
HY
E
MS
F
LB
G
CD1
H
PR
UAMS Subgroup
HY
LB
CD1
PR
CD2
MS
MF
I
PCA Quadrants by UAMS Subgroups
Percent
Q4
Q3
Q2
Q1
MF
CD2
HY
MS
LB
CD1
PR
J
UAMS Subgroups by Disease State
Percent
PR
MS
HY
LB
CD2
CD1
MF
MGUS
SMOL
NDMM
ERMM
LRMM
K
NDMM - PFS: UAMS Subgroups
Non-progression (%)
Time (days)
Events/N
PR 09/13
LB 25/39
MS 09/17
HY 24/56
CD1 11/16
CD2 18/34
MF 14/30
p = 0.027
L
NDMM - OS: UAMS Subgroups
Overall survival (%)
Time (days)
Events/N
PR 06/13
LB 16/39
MS 09/18
HY 11/56
CD1 06/16
CD2 07/34
MF 05/30
p = 0.0028
M
Pearson' correlation coefficient (r)
INTERFERON GAMMA RESPONSE
INTERFERON ALPHA RESPONSE
UV RESPONSE DN
HEDGEHOG SIGNALING
P53 PATHWAY
CHOLESTEROL HOMEOSTASIS
TGF BETA SIGNALING
NOTCH SIGNALING
PANCREAS BETA CELLS
BILE ACID METABOLISM
PROTEIN SECRETION
ANDROGEN RESPONSE
ESTROGEN RESPONSE LATE
ESTROGEN RESPONSE EARLY
TNFA SIGNALING VIA NFKB
IL2 STAT5 SIGNALING
HYPOXIA
APICAL SURFACE
EPITHELIAL MESENCHYMAL TRANSITIC
KRAS SIGNALING UP
COAGULATION
ANGIOGENESIS
INFLAMMATORY RESPONSE
IL6 JAK STAT3 SIGNALING
COMPLEMENT
APOPTOSIS
ALLOGRAFT REJECTION
OXIDATIVE PHOSPHORYLATION
GLYCOLYSIS
PEROXISOME
FATTY ACID METABOLISM
ADIPOGENESIS
MYC TARGETS V2
SPERMATOGENESIS
MITOTIC SPINDLE
G2M CHECKPOINT
E2F TARGETS
CD1 UP
CD1 VS CD2 UP
CD1 AND CD2 UP
HP DN
LB DN
CD1 AND CD2 DN
HP UP
MS DN
CD2 DN
CD1 DN
MF DN
CD1 VS CD2 DN
PR DN
LB UP
CD2 UP
MF UP
MS UP
PR UP
β Supercluster
α Supercluster

Figure S4. Association between UAMS subgroups and PCA quadrants. A, PCA score plot of 821 samples with RNA-Seq data, colored by UAMS groups. **B-H**, Samples visualized according to their UAMS status. **I**, Distribution of UAMS subgroups across PCA quadrants. **J**, Distribution of UAMS subgroups across disease state. **K-L**, (**K**) Progression-free and (**L**) overall survival of NDMM samples classified by UAMS subgroup. *P*-values for Log-rank (Mantel-Cox) test. **M**, Hierarchical clustering of ssGSEA NES correlations between Cancer Hallmarks and UAMS gene sets across all patients with RNA-seq data. Hallmarks that belong to superclusters **α** and **β** are indicated. This analysis identified "CD-2", "MF", and "MS" subgroups enriched for supercluster **β** genes, while "PR" overexpresses supercluster **α** genes.

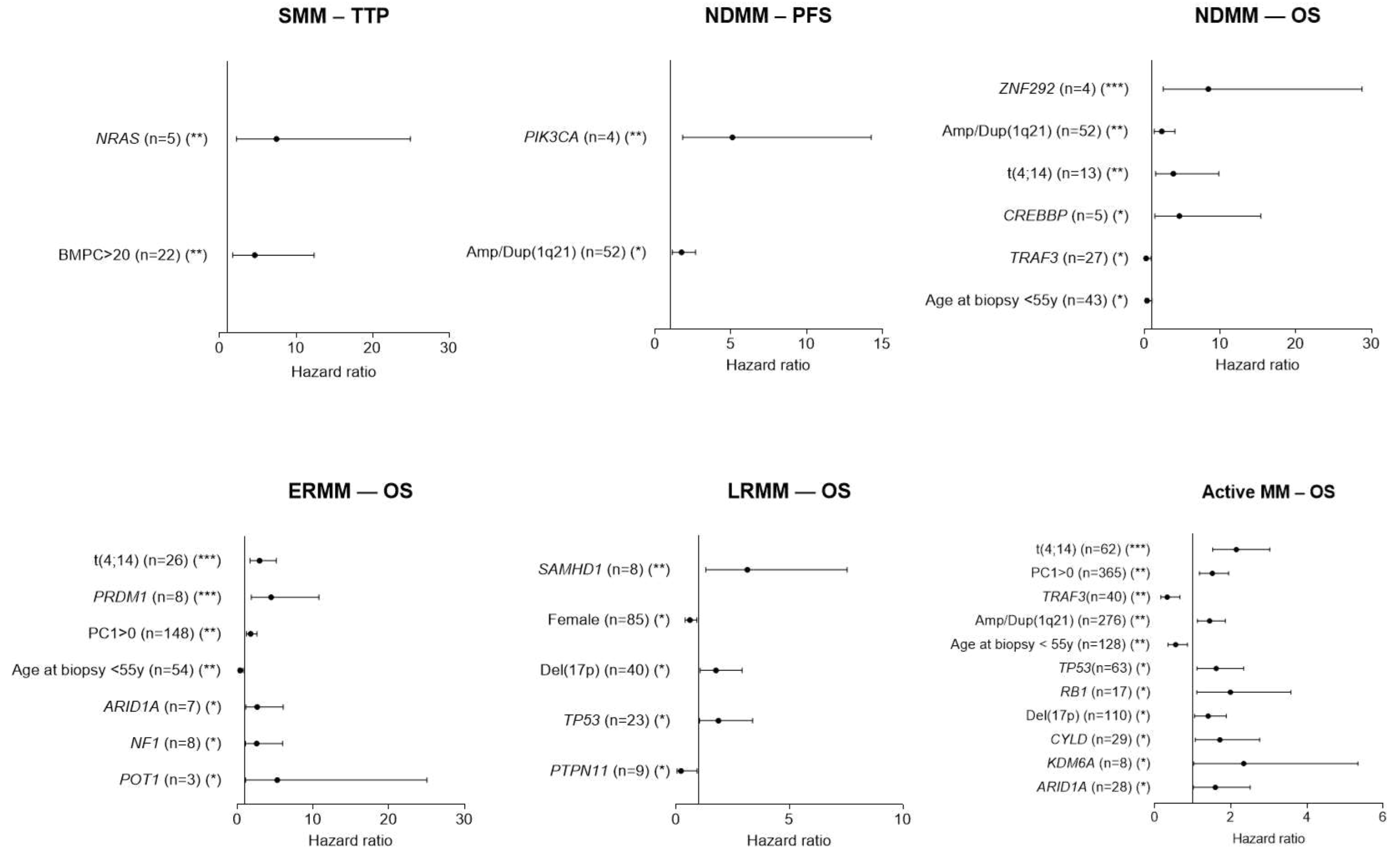

Figure S5. Forest plots from multivariable Cox proportional hazard models showing significant features. These plots display independent prognostic features associated with (**A**) TTP in SMM, (**B**) PFS in NDMM, and OS in (**C**) NDMM, (**D**) ERMM, (**E**) LRMM, and (**F**) Active MM. PC1>0 appears as an independent prognostic feature linked to shorter OS in ERMM and Active MM. (***) $P<0.001$; (**) $P<0.01$; (*) $P<0.05$.

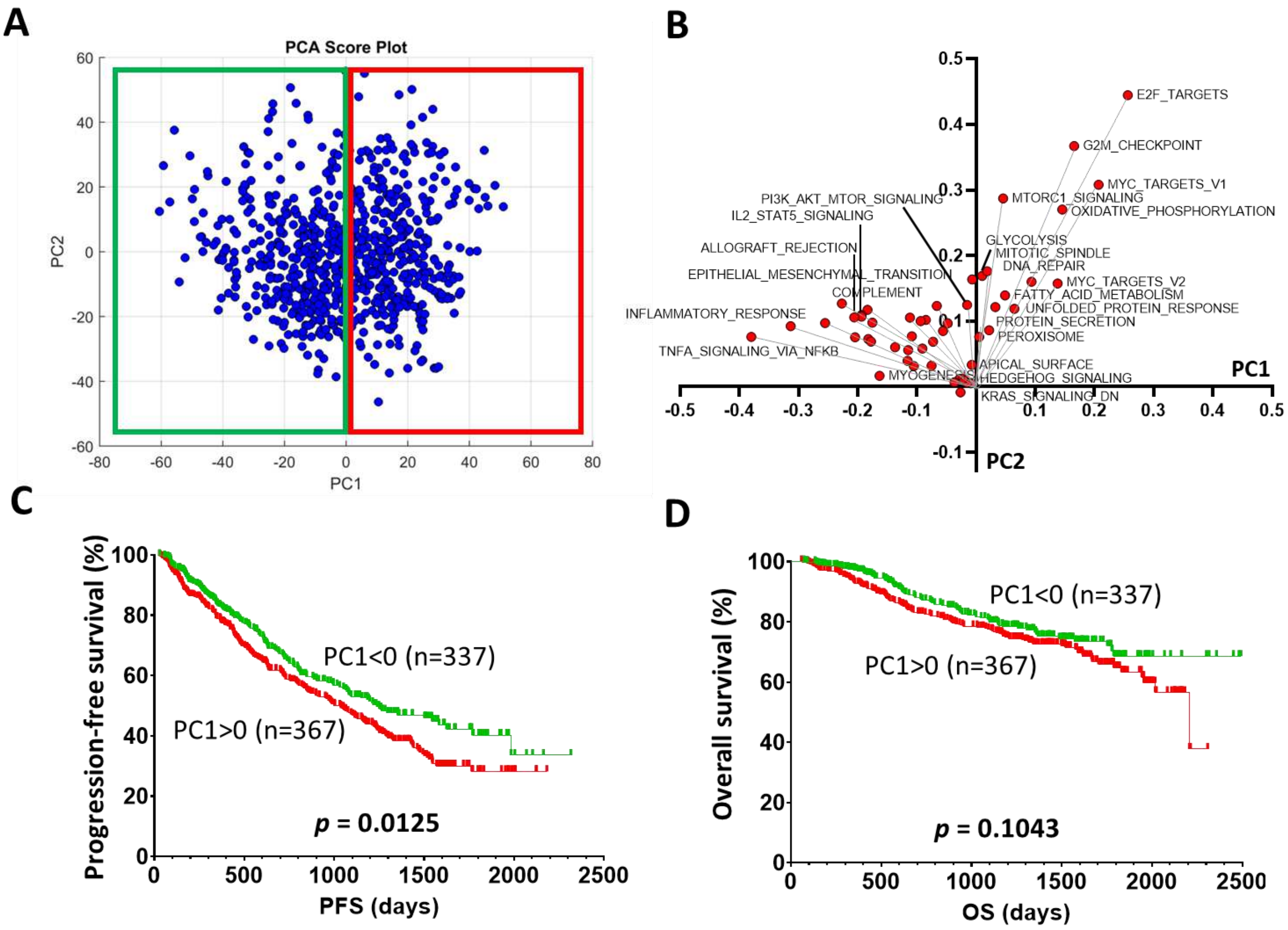

Figure S6. Transcriptomic profile bears prognostic value in an independent cohort of MM patients. A, PCA's score plot of 704 NDMM samples from the MMRF-CoMMpass dataset. The green and red rectangles delimit samples with negative and positive PC1 values, respectively. Single sample-GSEA (ssGSEA) NES for the 50 Cancer Hallmarks were used as variables in the analysis. **B**, Loading plot showing the relative contribution of each Cancer Hallmark in sample separation. **C**, Association between PC1 values and PFS. **D**, Borderline association between PC1 and OS. (Median follow-up time of the cohort: 42 months). *P*-values from Log-rank (Mantel-Cox) test.

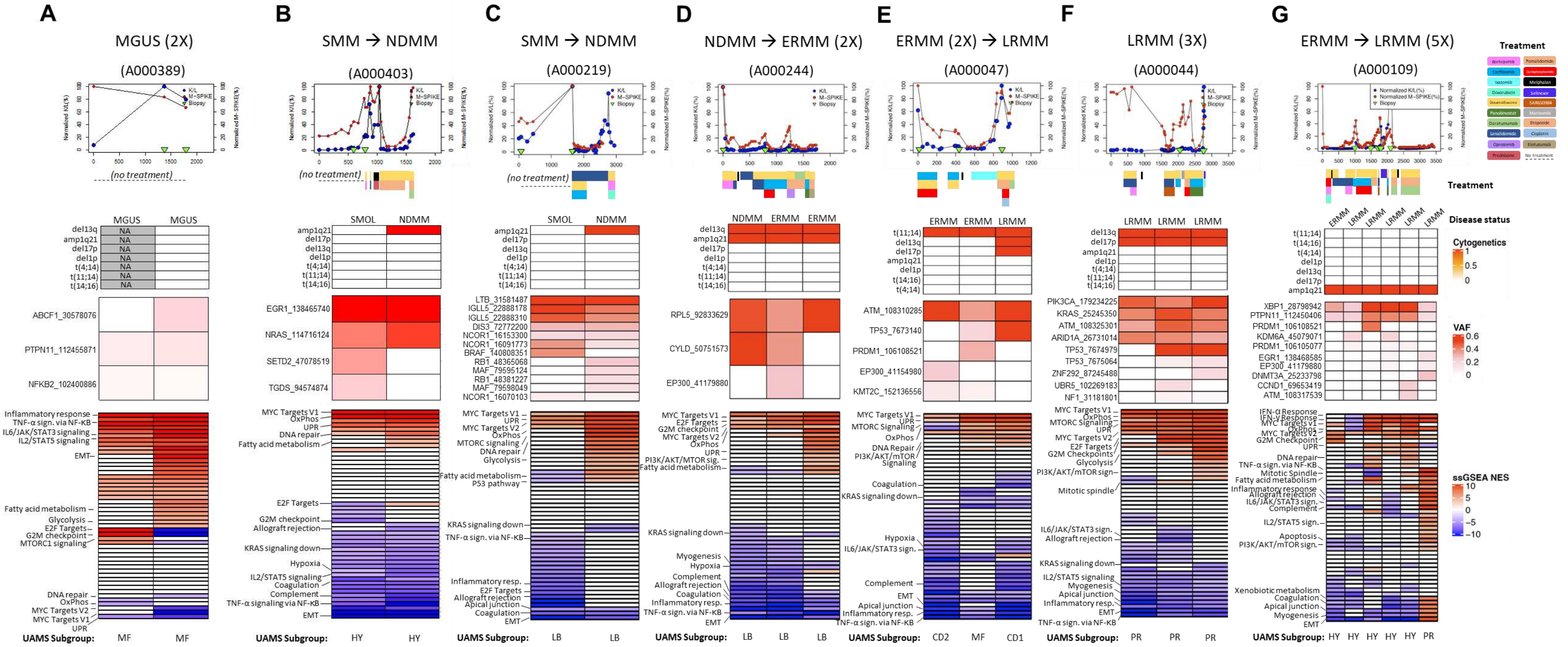

Figure S7. Transcriptional changes in longitudinal samples. Clinical, cytogenetic, and molecular characterization of sequential biopsies in (**A**) premalignant disease, (**B-C**) progression from SMM to NDMM, and (**D-G**) Active disease. Each column brings the molecular information of the corresponding biopsy, indicated by a green triangle in the K/L ratio/M-spike chart. Molecular variations across sequential biopsies highlight subclonal drift driven by increased proliferation and/or therapy selection.

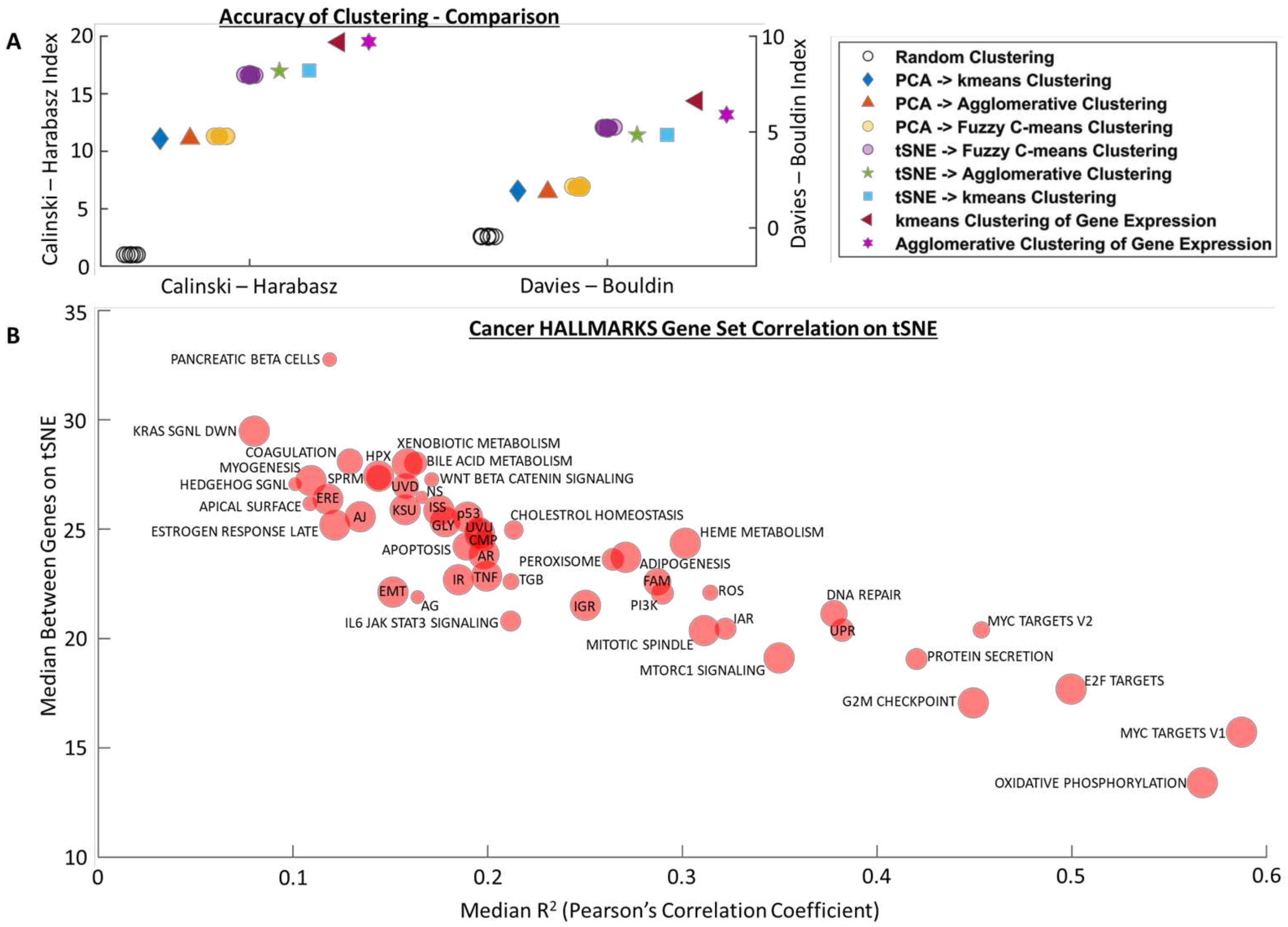

Figure S8. t-SNE and Fuzzy C-means are effective clustering approaches to represent gene co-expression. A, Calinski-Harabasz and Davies-Bouldin clustering indices comparing accuracy of clustering 16,738 genes into 500 clusters randomly, PCA followed by clustering, t-SNE followed by clustering, and direct clustering of gene expression. For the Calinski-Harabasz index a higher value indicates better clustering, while for the Davies-Bouldin index a lower value implies better clustering. **B**, Median distance between pairwise genes on t-SNE for each of the gene sets from Cancer Hallmarks compared with their median pairwise Pearson's correlation coefficient (R^2). The plot shows that highly correlated gene sets across all patients correspond to small distances on the t-SNE plot. The median distances on t-SNE and median R^2 values are negatively correlated with a Pearson correlation coefficient of 0.88. Abbreviations: KRAS SGNL DWN: KRAS_SIGNALING_DN; HEDGEHOG SGNL: HEDGEHOG_SIGNALING; HPX: HYPOXIA; SPRM: SPERMATOGENESIS; UVD: UV_RESPONSE_DN; NS: NOTCH_SIGNALING; KSU: KRAS_SIGNALING_UP; ERE: ESTROGEN_RESPONSE_EARLY; AJ: APICAL_JUNCTION; ISS: IL2_STAT5_SIGNALING; GLY: GLYCOLYSIS; p53: P53_PATHWAY; UVU: UV_RESPONSE_UP; CMP: COMPLEMENT; AR: ANDROGEN_RESPONSE; EMT: EPITHELIAL_MESENCHYMAL_TRANSITION; IR: INFLAMMATROY RESPONSE; TNF: TNFA_SIGNALING_VIA_NFKB; AG: ANGIOGENESIS; TGB: TGF_BETA_SIGNALING; IGR: INTERFERON_GAMMA_RESPONSE; FAM: FATTY_ACID_METABOLISM; PI3K: PI3K_AKT_MTOR_SIGNALING; ROS: REACTIVE_OXYGEN_SPECIES_PATHWAY; IAR: INTERFERON_ALPHA_RESPONSE; UPR: UNFOLDED_PROTEIN_RESPONSE.

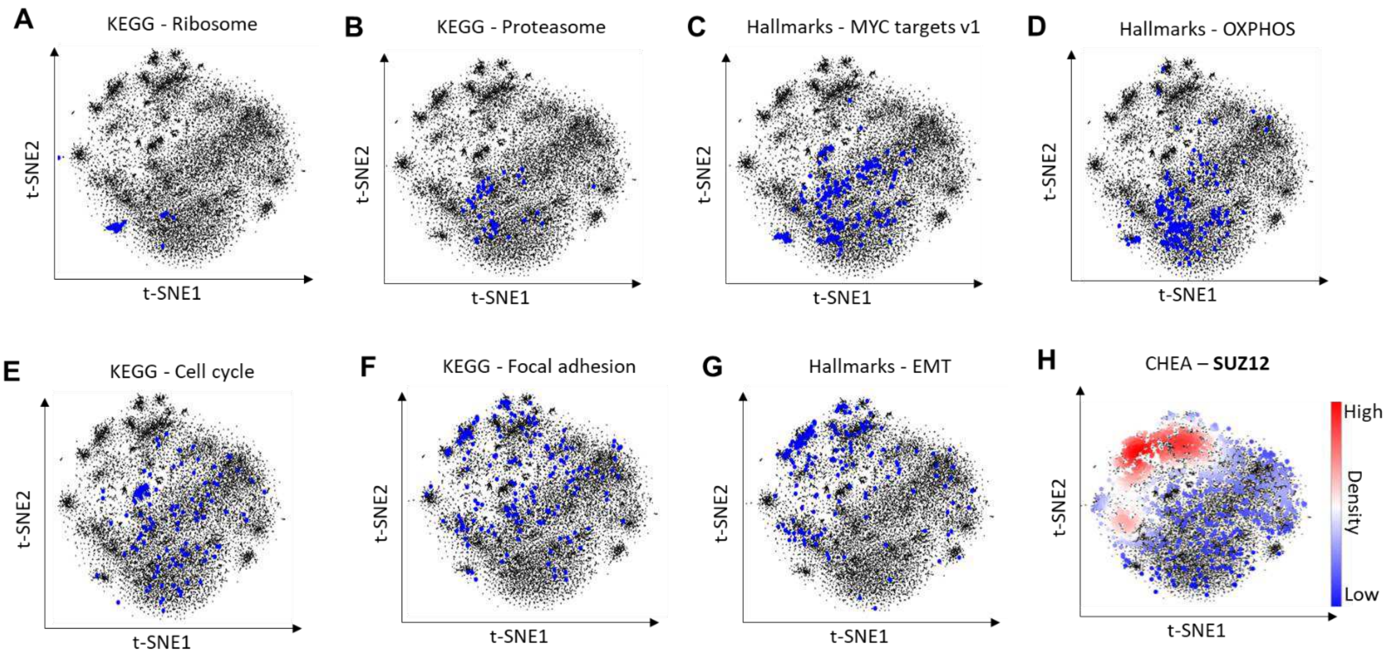

Figure S9. Topological signatures of biological pathways in MM transcriptional map. Projection of genes belonging to KEGG Pathways and Cancer Hallmarks on the MM transcriptional map, illustrating examples of (**A-B**) cohesively localized gene sets, indicating unified transcriptional control, and (**C-G**) scattered gene sets, suggesting diverse transcriptional regulatory mechanisms. **H**, Topological heat map of SUZ12 target genes.

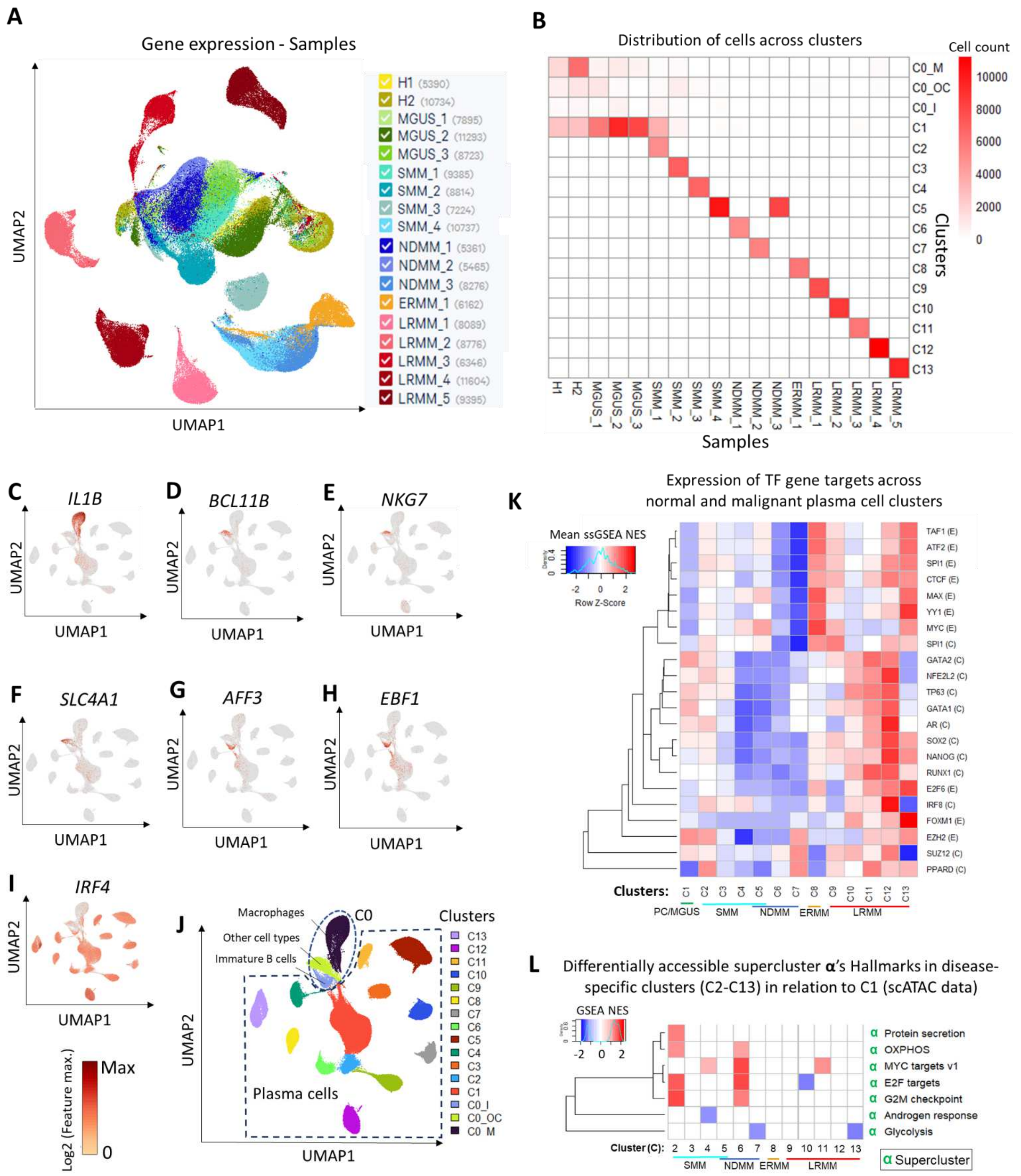

Figure S10. Paired single-cell ATAC/RNA-Seq data illustrates inter- and intrasample heterogeneity. A, UMAP plot for gene expression data (RNA-Seq compartment), colored according to sample origin. **B**, The distribution of cells per sample across the UMAP plot clusters (SMM_4 and NDMM_3 are sequential biopsies from the same patient). **(C-J)** Expression of (**C**) *IL1B* (associated with macrophages), (**D**) *BCL11B* (T cell precursors), (**E**) *NKG7* (NK and CD8+ T cells), (**F**) *SLC4A1* (mature erythroid cells), (**G**) *AFF3* and (**H**) *EBF1* (immature B cells), and (**I**) *IRF4* (PCs), visualized in the UMAP plot for the ATAC compartment indicate that (**J**) cluster C0 is mainly

composed of macrophages, other cell types and immature B cells, while clusters C1 to C13 are composed of PCs. **K**, Mean ssGSEA normalized enrichment score, on gene expression data, for the transcription factors enriched in the MGUS-to-SMM and NDMM-to-LRMM transitions (bulk analysis; please refer to **Figures 2C** and **2F**). **L**, GSEA NES (ΔC2-13,C1) for supercluster **α** Cancer Hallmarks among differentially accessible genes in clusters C2-C13 in relation to C1. Only statistically significant Hallmarks in at least one comparison are shown.

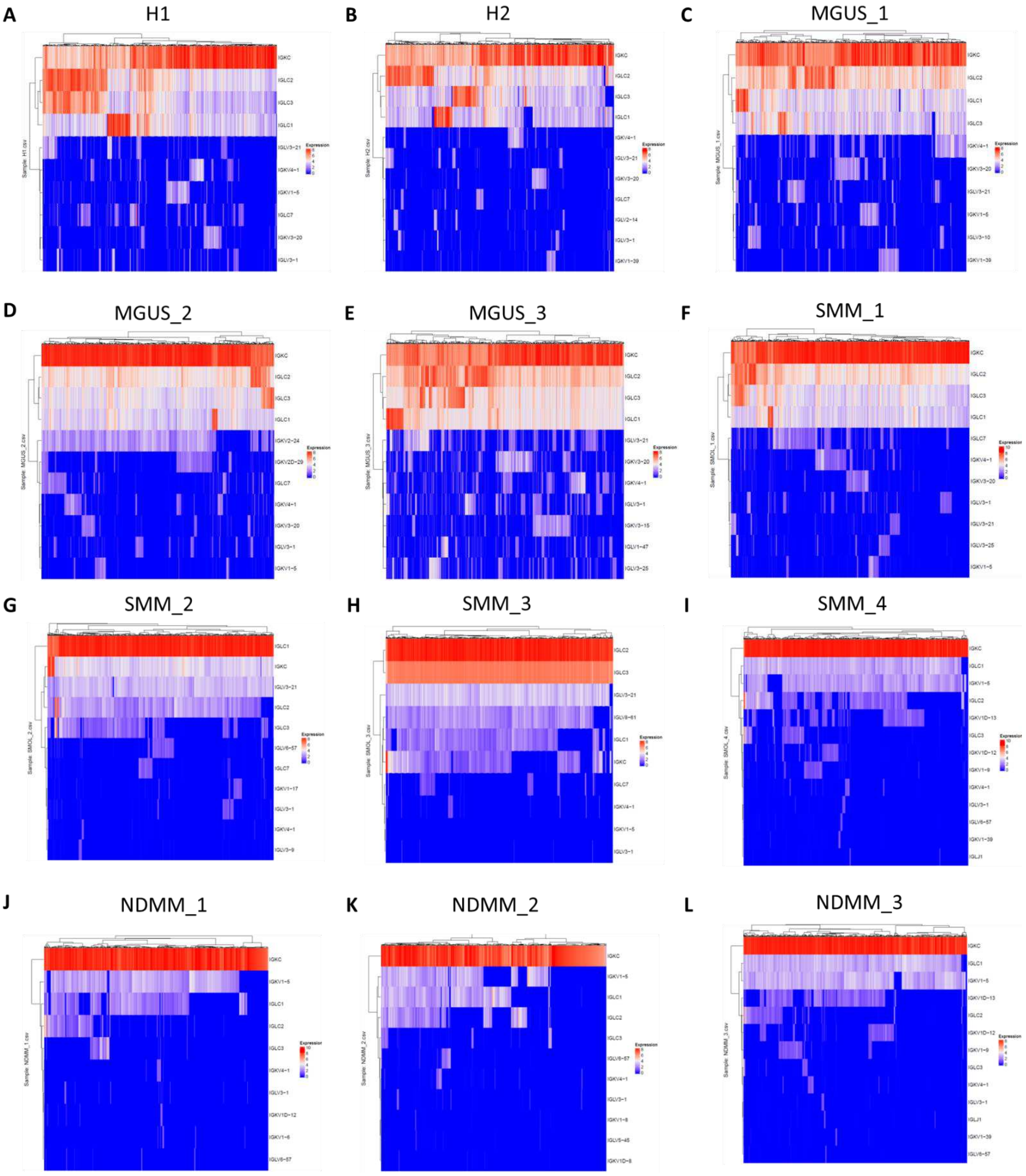
A
H1
B
H2
C
MGUS_1
D
MGUS_2
E
MGUS_3
F
SMM_1
G
SMM_2
H
SMM_3
I
SMM_4
J
NDMM_1
K
NDMM_2
L
NDMM_3

(cont.)

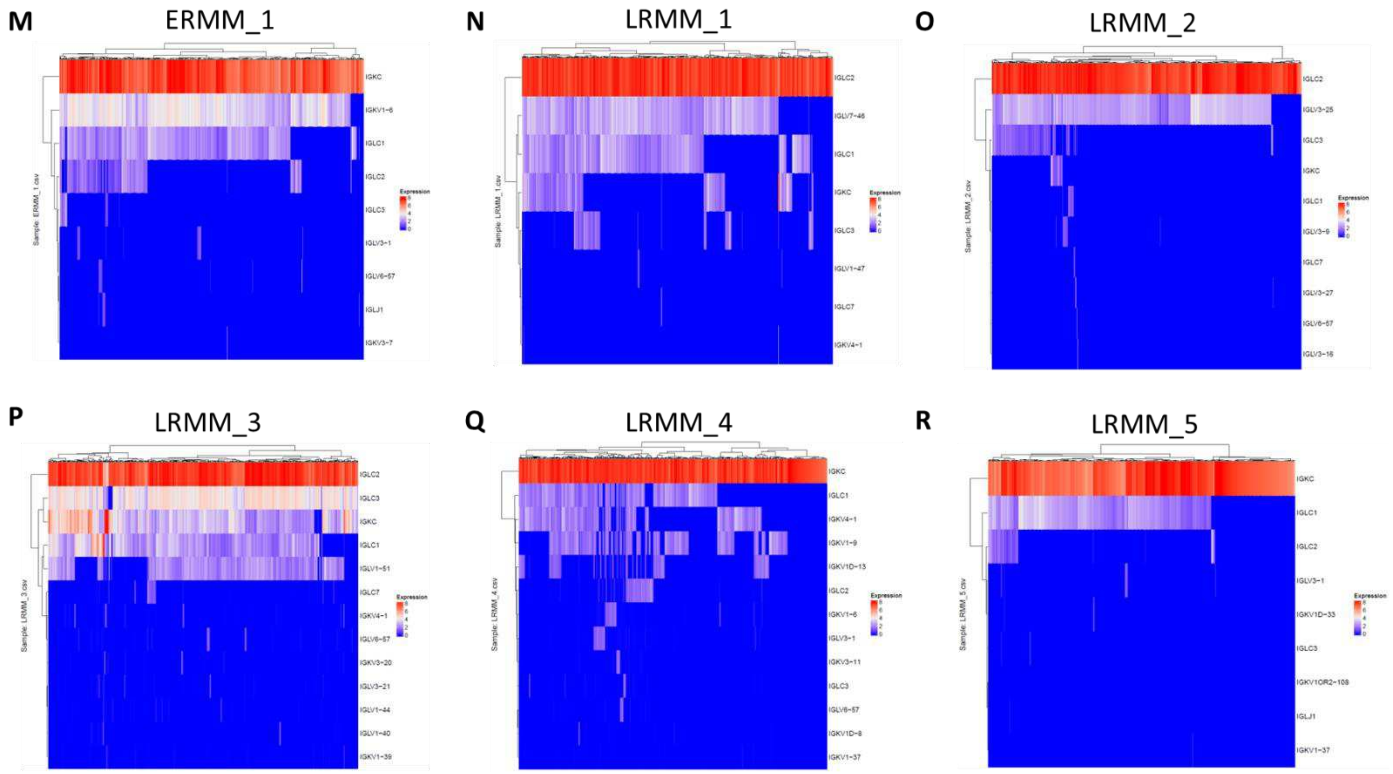

Figure S11. PC clonality across samples with scMultiome data. Single-cell gene expression analysis of immunoglobulin genes reveals the presence of PC subpopulations in samples from (**A**) healthy donor 1, (**B**) healthy donor 2, (**C)** MGUS_1, and (**E**) MGUS_3. In contrast, (**D**) MGUS_2, as well as all (**F–I**) SMM, (**J–L**) NDMM, (**M**) ERMM, and (**N–R**) LRMM samples appear clonal, indicating a single PC population.

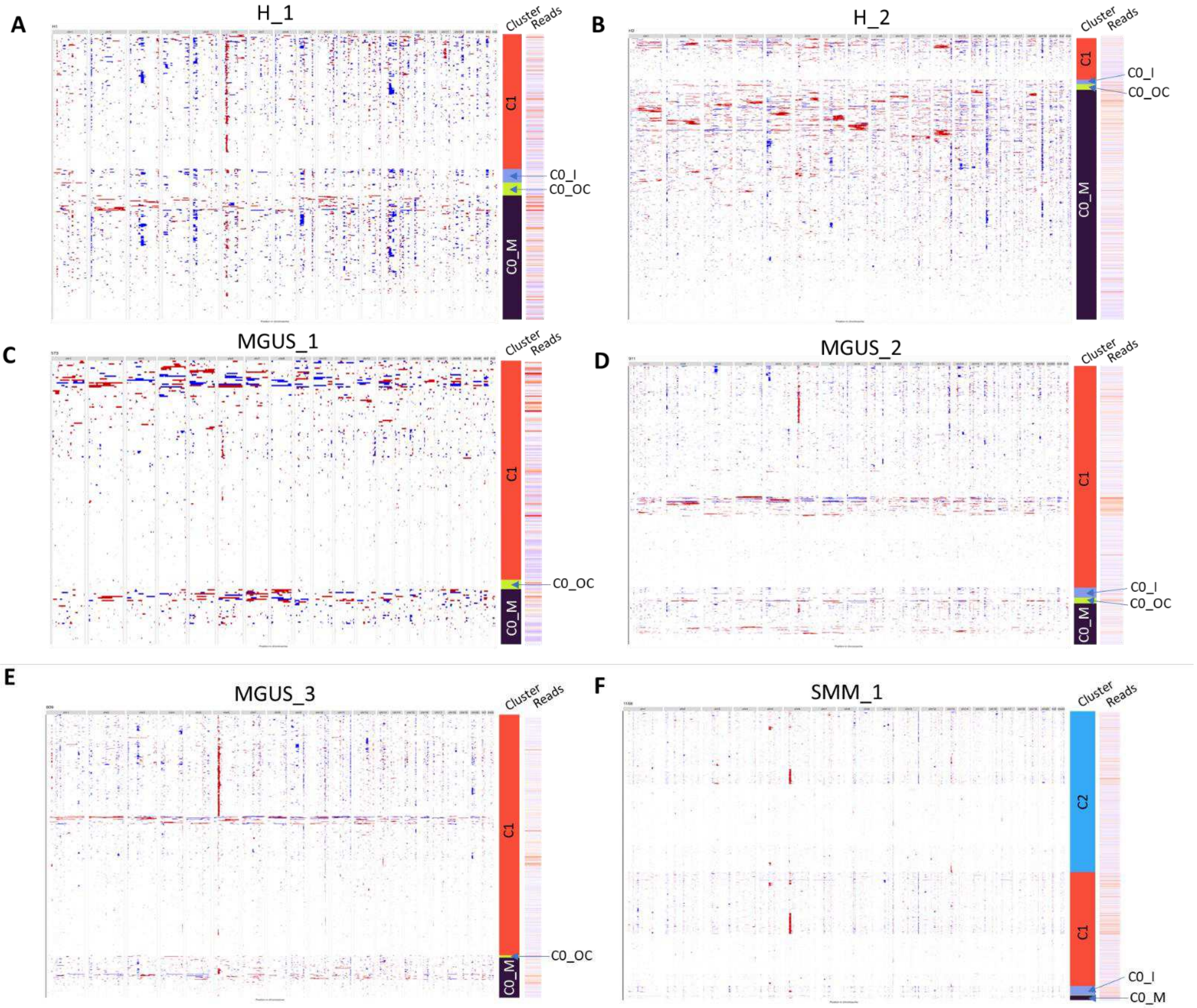

Figure S12. Karyograms of healthy donors, MGUS and SMM samples. Plots illustrating copy number alterations (red=gain; blue=loss) from chromatin accessibility data at the single-cell level using epiAneufinder in (**A**) healthy donor 1, (**B**) healthy donor 2, (**C**) MGUS_1, (**D**) MGUS_2, (**E**) MGUS_3, and (**F**) SMM_1. "Cluster" = cluster number in the UMAP plot. "Reads" = normalized number of reads per cell.

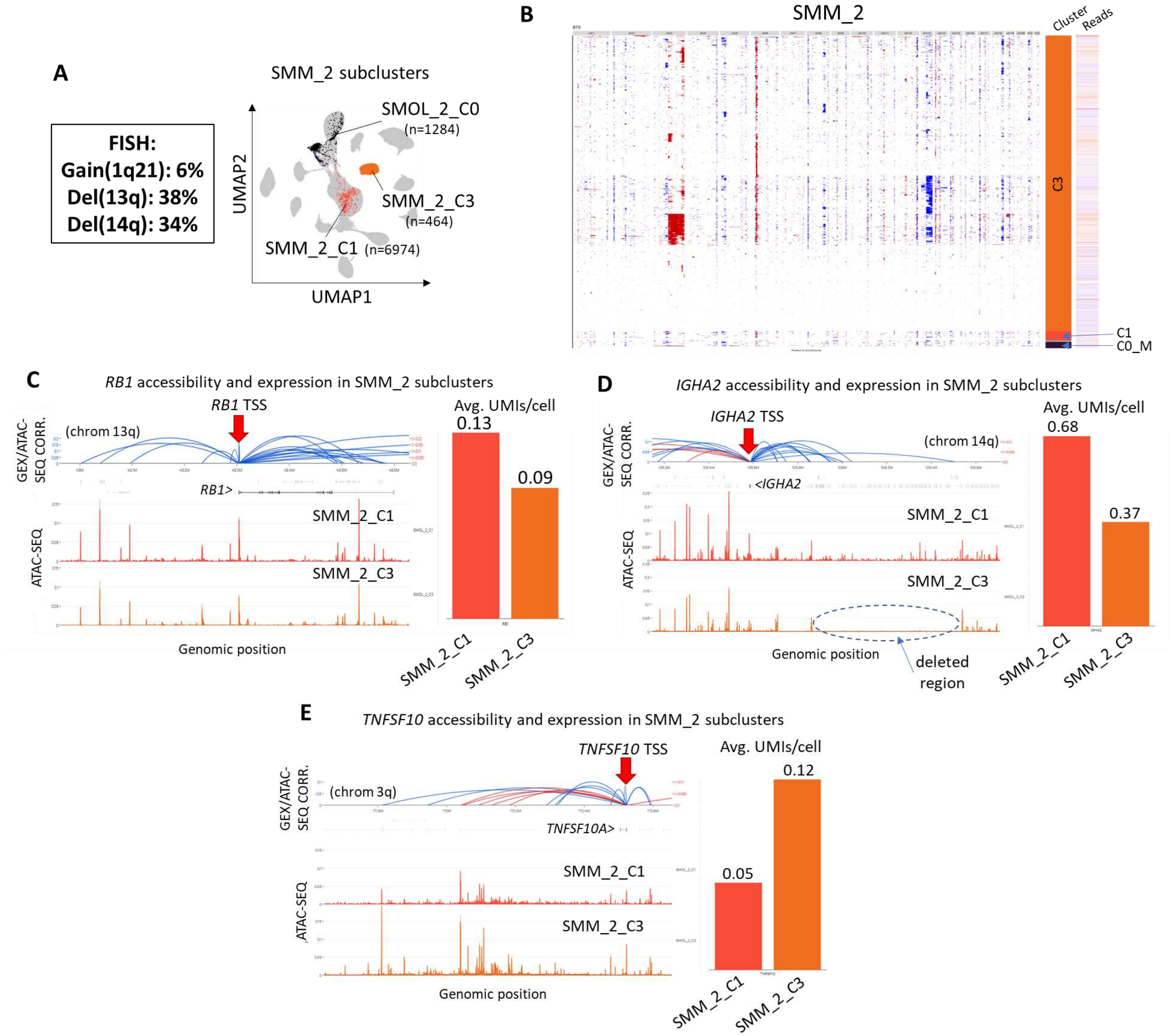

Figure S13. Differential gene accessibility and expression across SMM_2 subclusters. This sample was identified to have Gain(1q21), Del(13q), and Del(14q) by FISH analysis; percentages refer to the proportion of cells harboring the abnormality. **A,** SMM_2 subclusters. **B**, Karyogram illustrating copy number alterations (red=gain; blue=loss) from chromatin accessibility data at the single-cell level using epiAneufinder. "Cluster" = cluster number in the UMAP plot. "Reads" = normalized number of reads per cell. **C**, Left, Proportion of cells per subcluster with peaks in ATAC around *RB1*, a gene in chromosome 13q region; right, expression of *RB1* across SMM_2 subclusters. TSS = transcription starting site. **D**, Idem **C**, for the *IGHA2* gene (the probed used in FISH to detect abnormalities in chromosome 14q); the absence of signal in the vicinity of the *IGHA2* gene coincides with FISH data. **E**, An anecdotal example of gene (*TNFSF10*) located on chromosome 3q. The ATAC data suggests amplification of the region – confirming EpiAneufinder results –, not identified via FISH due to the absence of a probe specific to that chromosomal region.

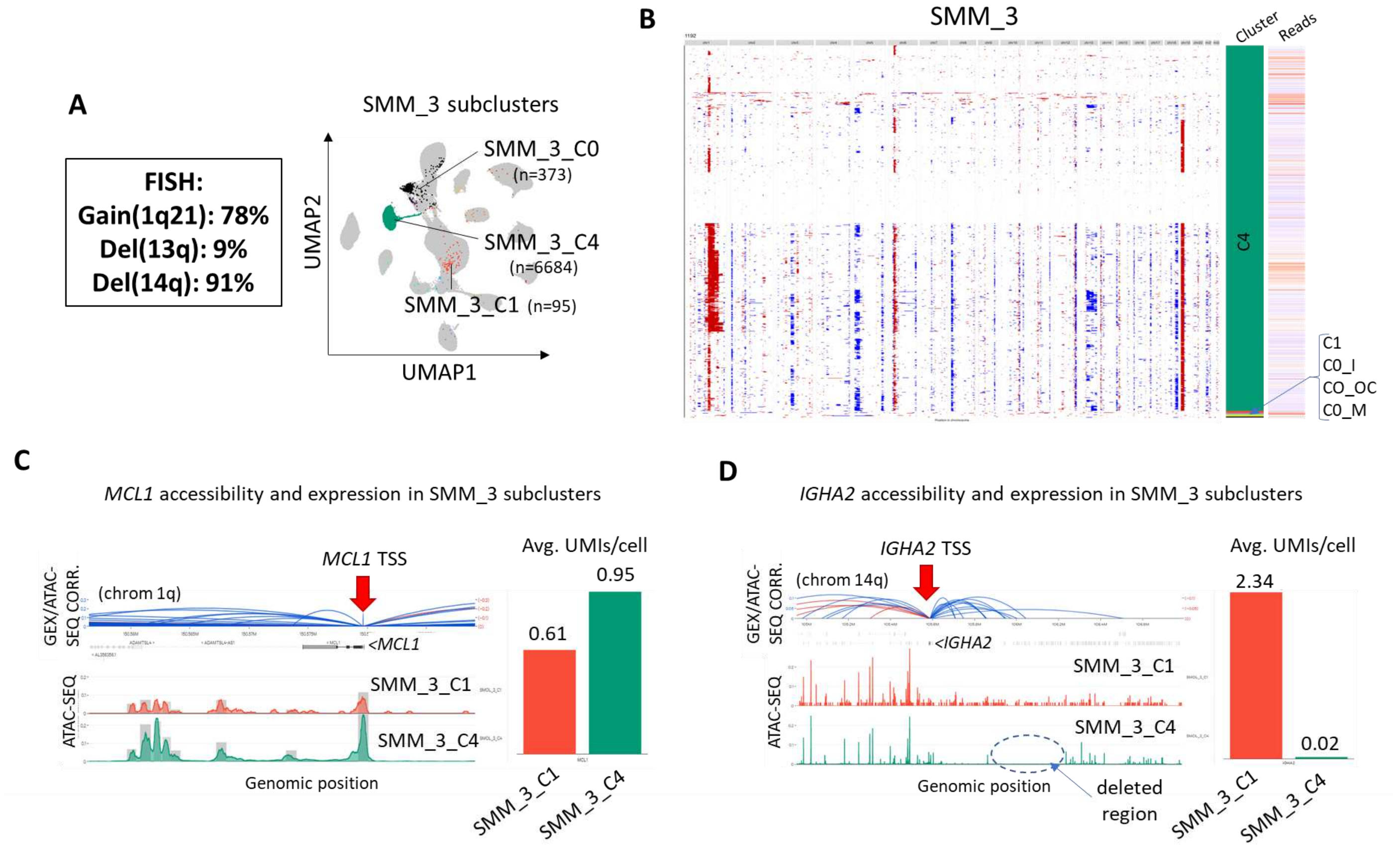

Figure S14. Differential gene accessibility and expression across SMM_3 subclusters. This sample was identified to have Gain(1q21), Del(13q), and Del(14q) by FISH analysis; percentages refer to the proportion of cells harboring the abnormality. **A,** SMM_3 subclusters. **B**, Karyogram illustrating copy number alterations (red=gain; blue=loss) from chromatin accessibility data at the single-cell level using epiAneufinder. “Cluster” = cluster number in the UMAP plot. “Reads” = normalized number of reads per cell. **C**, Left, proportion of cells per subcluster with peaks in ATAC around *MCL1*, a gene localized in the chromosome 1q21 region; right, expression of *MCL1* across SMM_3 subclusters. TSS = transcription starting site. **D**, Idem **C**, for the *IGHA2* gene (the probed used in FISH to detect abnormalities in chromosome 14q); the absence of signal in the vicinity of the *IGHA2* gene coincides with FISH data.

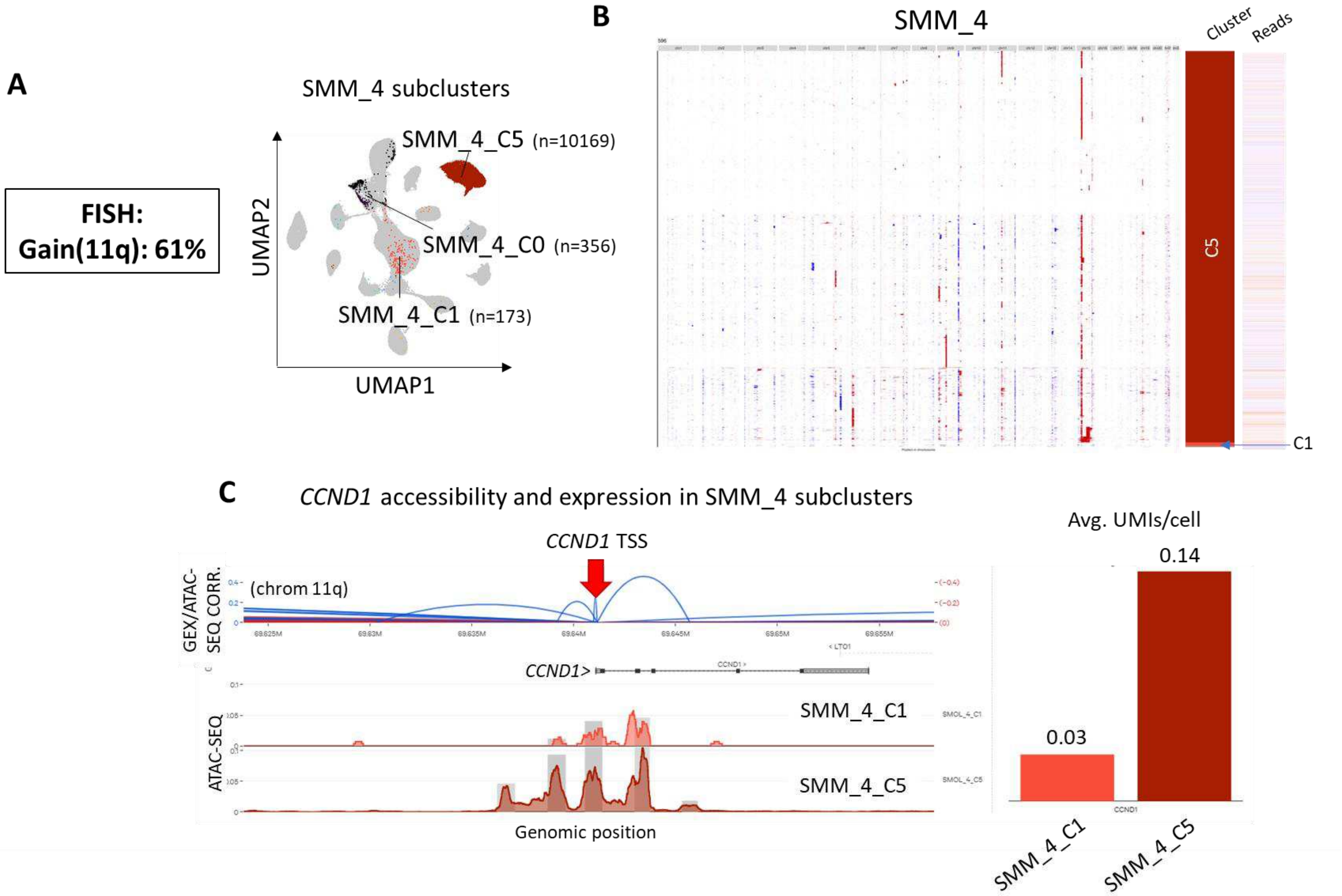

Figure S15. Differential gene accessibility and expression across SMM_4 subclusters. This sample was identified to have Gain(11q) by FISH analysis; percentages refer to the proportion of cells harboring the abnormality. **A,** SMM_4 subclusters. **B**, Karyogram illustrating copy number alterations (red=gain; blue=loss) from chromatin accessibility data at the single-cell level using epiAneufinder. "Cluster" = cluster number in the UMAP plot. "Reads" = normalized number of reads per cell. **C**, Left, proportion of cells per subcluster with peaks in ATAC around *CCND1*, a gene localized in the chromosome 11q region; right, expression of *CCND1* across SMM_4 subclusters. TSS = transcription starting site.

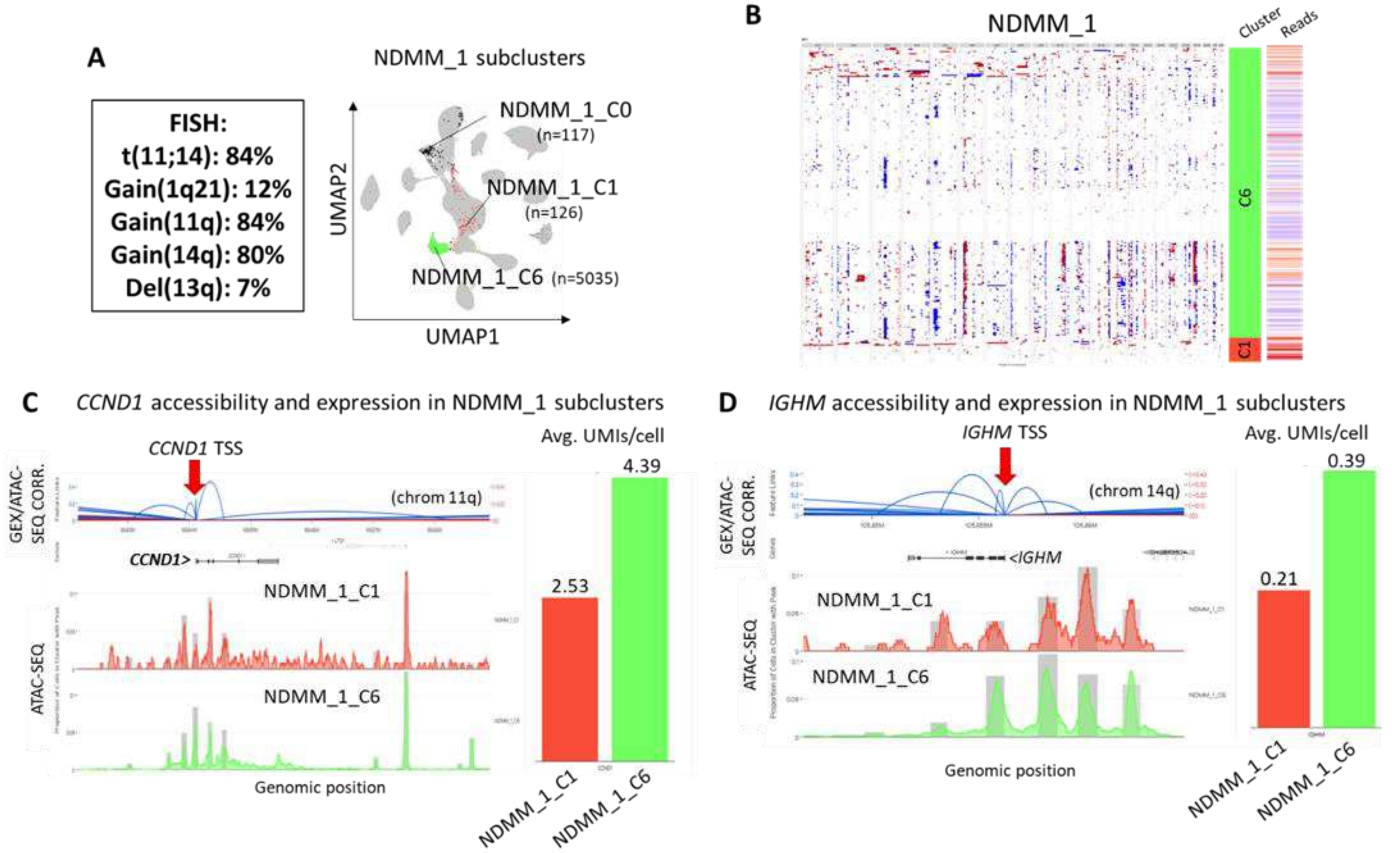

Figure S16. Differential gene accessibility and expression across NDMM_1 subclusters. This sample was identified to have t(11;14), Gain(1q21), Gain(11q), Gain(14q) and Del(13q) by FISH analysis; percentages refer to the proportion of cells harboring the abnormality. **A,** NDMM_1 subclusters. **B**, Karyogram illustrating copy number alterations (red=gain; blue=loss) from chromatin accessibility data at the single-cell level using epiAneufinder. “Cluster” = cluster number in the UMAP plot. “Reads” = normalized number of reads per cell. **C**, Left, proportion of cells per subcluster with peaks in ATAC around *CCND1*, a gene overexpressed in t(11;14); right, expression of *CCND1* across NDMM_1 subclusters. **D**, Idem **C**, for *IGHM*, a gene localized in chromosome 14q region. TSS = transcription starting site.

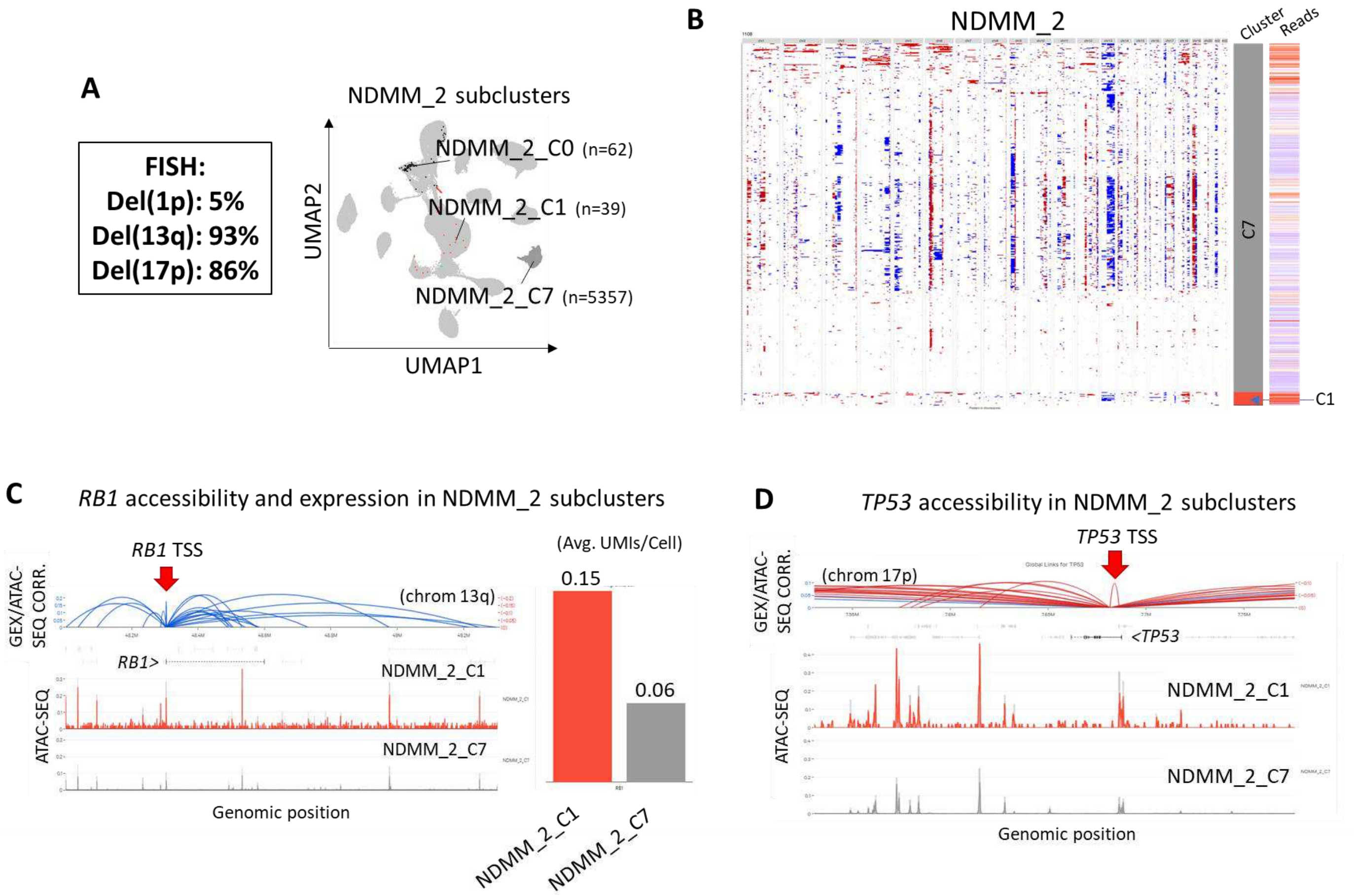

Figure S17. Differential gene accessibility and expression across NDMM_2 subclusters. This sample was identified to have Del(1p), Del(13q) and Del(17p) by FISH analysis; percentages refer to the proportion of cells harboring the abnormality. **A,** NDMM_2 subclusters. **B**, Karyogram illustrating copy number alterations (red=gain; blue=loss) from chromatin accessibility data at the single-cell level using epiAneufinder. “Cluster” = cluster number in the UMAP plot. “Reads” = normalized number of reads per cell. **C**, Left, proportion of cells per subcluster with ATAC peaks around *RB1*, a gene localized in the chromosome 13q region; right, expression of *RB1* across NDMM_2 subclusters. **D**, Proportion of cells per subcluster with peaks in ATAC around *TP53*, a gene localized in the chromosome 17p region; no expression of *TP53* was observed across NDMM_2 subclusters – the limited number of cells in cluster C1 may represent a caveat when comparing this gene. TSS = transcription starting site.

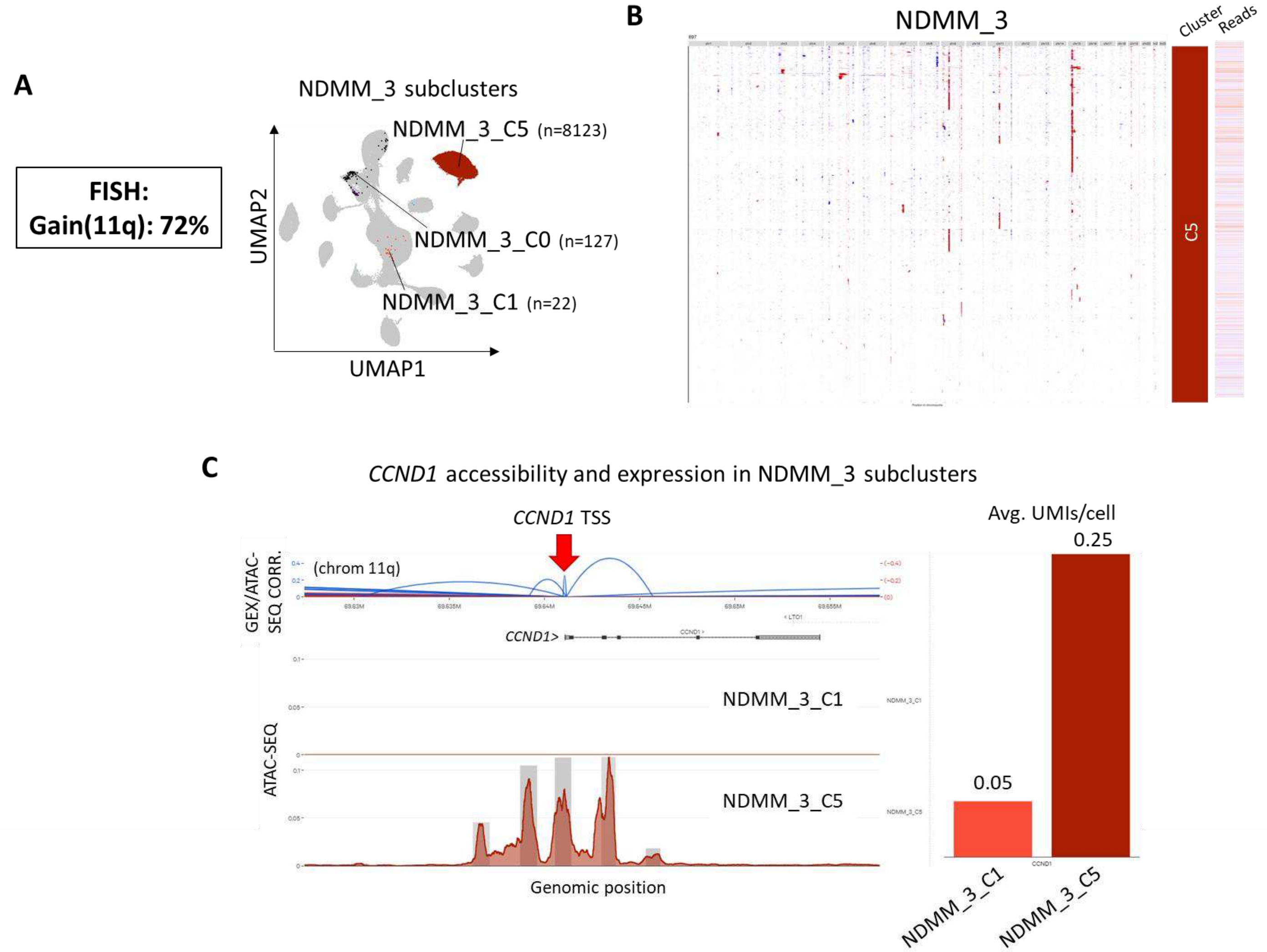

Figure S18. Differential gene accessibility and expression across NDMM_3 subclusters. This sample was identified to have Gain(11q) by FISH analysis; percentages refer to the proportion of cells harboring the abnormality. **A,** NDMM_3 subclusters. **B**, Karyogram illustrating copy number alterations (red=gain; blue=loss) from chromatin accessibility data at the single-cell level using epiAneufinder. “Cluster” = cluster number in the UMAP plot. “Reads” = normalized number of reads per cell. **C**, Left, proportion of cells per subcluster with peaks in ATAC around *CCND1*, a gene localized in the chromosome 11q region; right, expression of *CCND1* across NDMM_1 subclusters. TSS = transcription starting site.

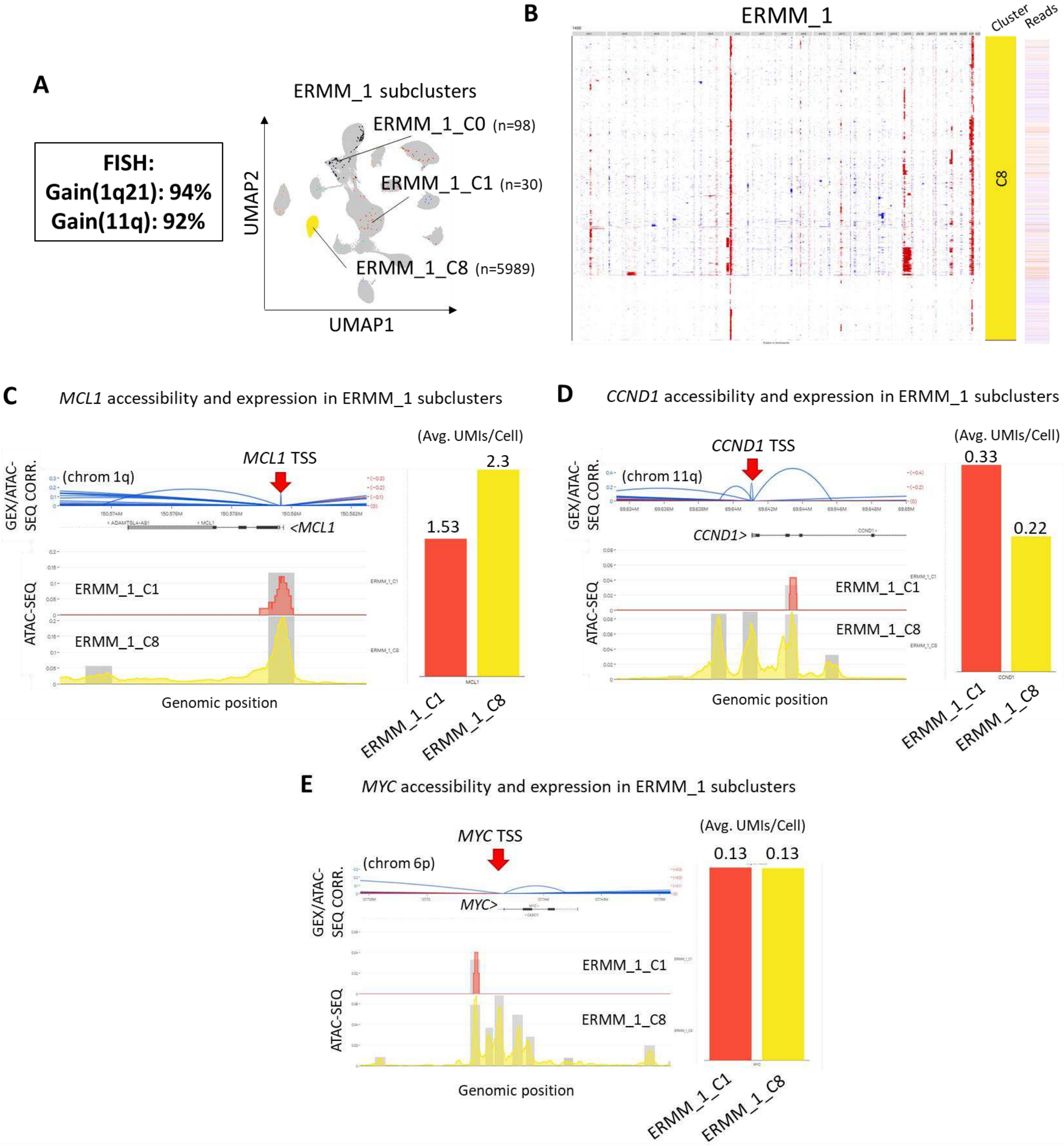

Figure S19. Differential gene accessibility and expression across ERMM_1 subclusters. This sample was identified to have Gain(1q21) and Gain(11q) by FISH analysis; percentages refer to the proportion of cells harboring the abnormality. **A,** ERMM_1 subclusters – the small number of cells in cluster C1 makes the comparison less precise in subsequent analyses (a caveat for comparison). **B,** Karyogram illustrating copy number alterations (red=gain; blue=loss) from chromatin accessibility data at the single-cell level using epiAneufinder. "Cluster" = cluster number in the UMAP plot. "Reads" = normalized number of reads per cell. **C**, Left, proportion of cells per subcluster with ATAC peaks around *MCL1*, a gene localized in the chromosome 1q21 region; right, expression of *MCL1* across NDMM_2 subclusters. **D-E**, Idem **C**, for (**D**) *CCND1*, a gene localized in chromosome 11q region, and (**E**) *MYC*, a gene localized in the chromosome 6p region. TSS = transcription starting site.

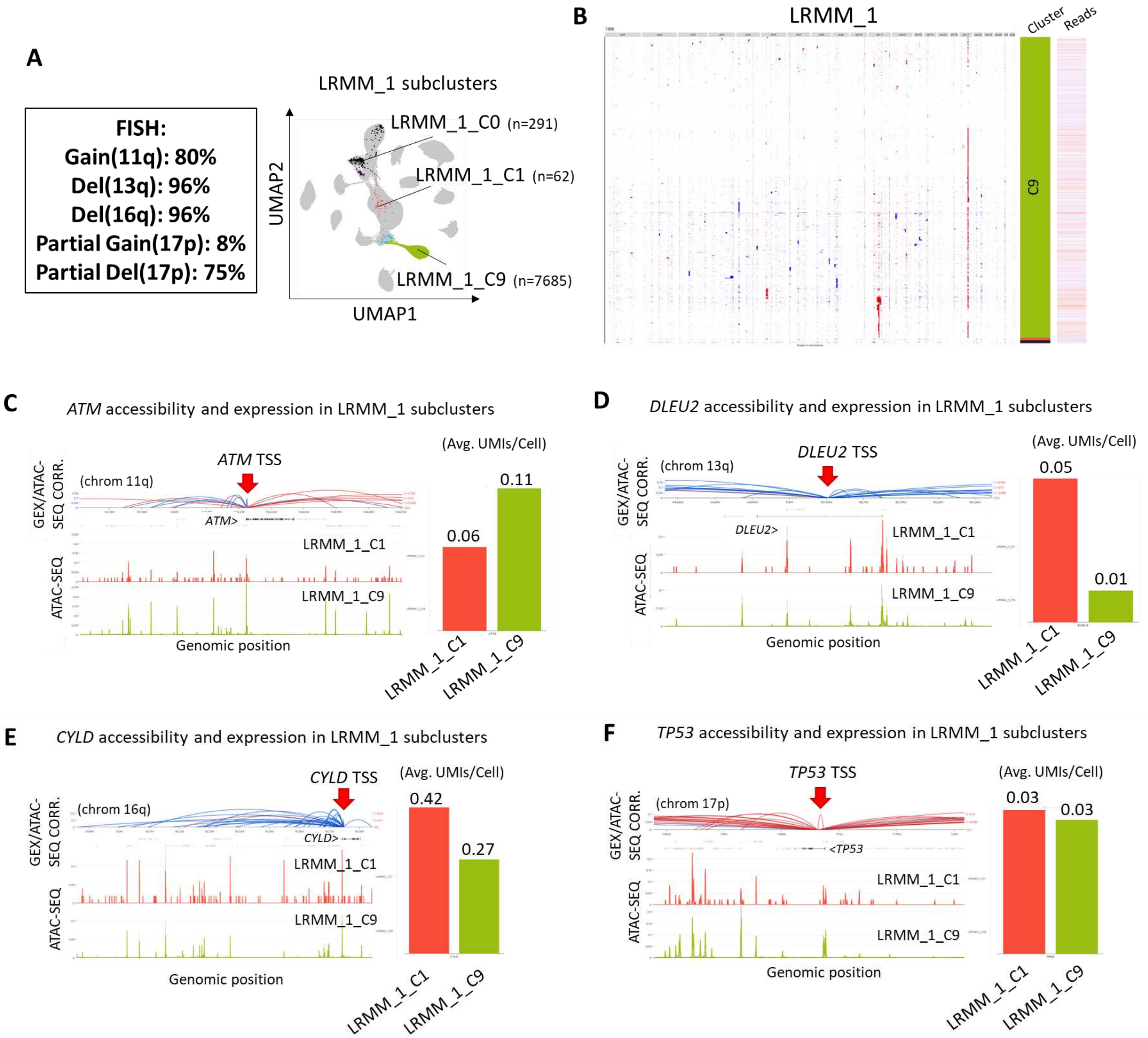

Figure S20. Differential gene accessibility and expression across LRMM_1 subclusters. This sample was identified to have Gain(11q), Del(13q), Del(16q), partial Gain(17p), and partial Del(17p) by FISH analysis; percentages refer to the proportion of cells harboring the abnormality. **A,** LRMM_1 subclusters. **B,** Karyogram illustrating copy number alterations (red=gain; blue=loss) from chromatin accessibility data at the single-cell level using epiAneufinder. "Cluster" = cluster number in the UMAP plot. "Reads" = normalized number of reads per cell. **C**, Left, proportion of cells per subcluster with ATAC peaks around *ATM*, a gene localized in the chromosome 11q region; right, expression of *ATM* across LRMM_1 subclusters. **D-F**, Idem **C**, for (**D**) *DLEU2*, a gene localized in the chromosome 13q region; (**E**) *CYLD*, a gene localized in the chromosome 16q region, and (**F**) *TP53*, a gene localized in the chromosome 17p region – the limited number of cells in cluster C1 may represent a caveat when comparing this gene. TSS = transcription starting site.

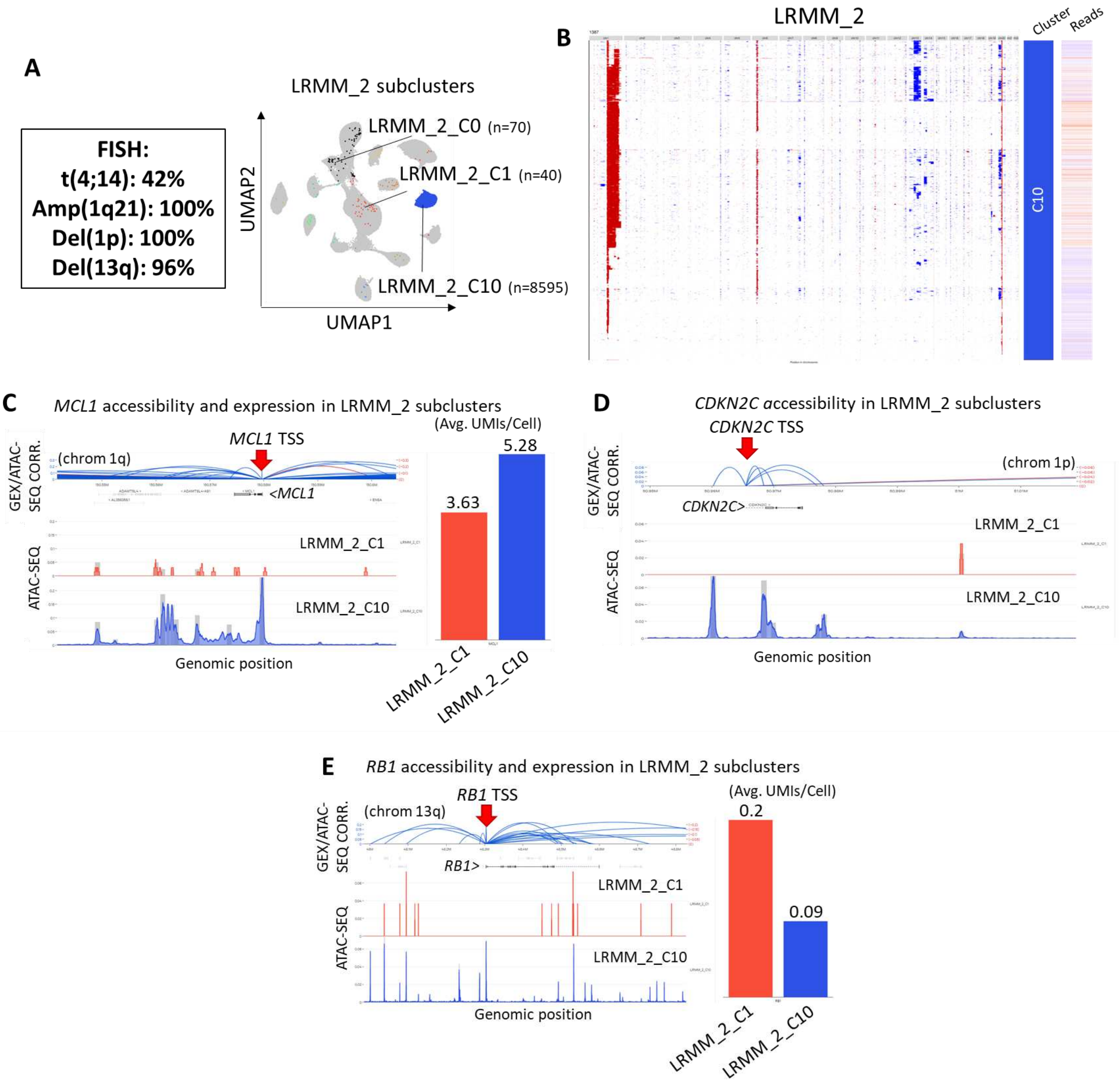

Figure S21. Differential gene accessibility and expression across LRMM_2 subclusters. This sample was identified to have t(4;14), Amp(1q21), Del(1p), and Del(13q) by FISH analysis; percentages refer to the proportion of cells harboring the abnormality. **A,** LRMM_2 subclusters. **B,** Karyogram illustrating copy number alterations (red=gain; blue=loss) from chromatin accessibility data at the single-cell level using epiAneufinder. "Cluster" = cluster number in the UMAP plot. "Reads" = normalized number of reads per cell. **C**, Left, proportion of cells per subcluster with ATAC peaks around *MCL1*, a gene localized in the chromosome 1q21 region; right, expression of *MCL1* across LRMM_2 subclusters. **D-E**, Idem **C**, for (**D**) *CDKN2C*, a gene localized in the chromosome 1p region (no expression of *CDKN2C* was observed across LRMM_2 subclusters), and (**E**) *RB1*, a gene localized in the chromosome 13q region. TSS = transcription starting site.

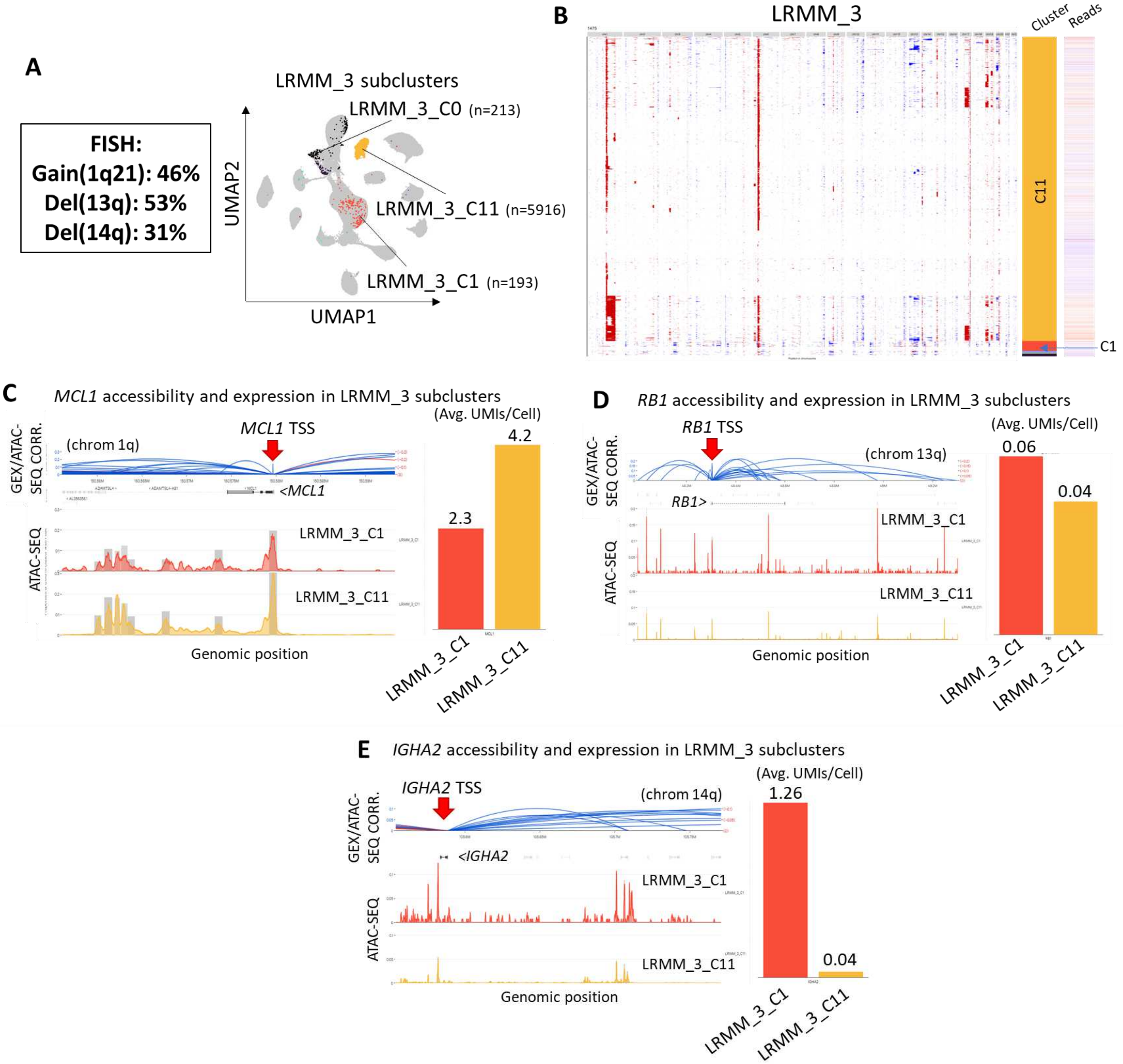

Figure S22. Differential gene accessibility and expression across LRMM_3 subclusters. This sample was identified to have Gain(1q21), Del(13q), and Del(14q) by FISH analysis; percentages refer to the proportion of cells harboring the abnormality. **A,** LRMM_3 subclusters. **B,** Karyogram illustrating copy number alterations (red=gain; blue=loss) from chromatin accessibility data at the single-cell level using epiAneufinder. "Cluster" = cluster number in the UMAP plot. "Reads" = normalized number of reads per cell. **C**, Left, proportion of cells per subcluster with ATAC peaks around *MCL1*, a gene localized in the chromosome 1q21 region; right, expression of *MCL1* across LRMM_3 subclusters. **D-E**, Idem **C**, for (**D**) *RB1*, a gene localized in the chromosome 13q region, and (**E**) *IGHA2*, a gene localized in the chromosome 14q region. TSS = transcription starting site.

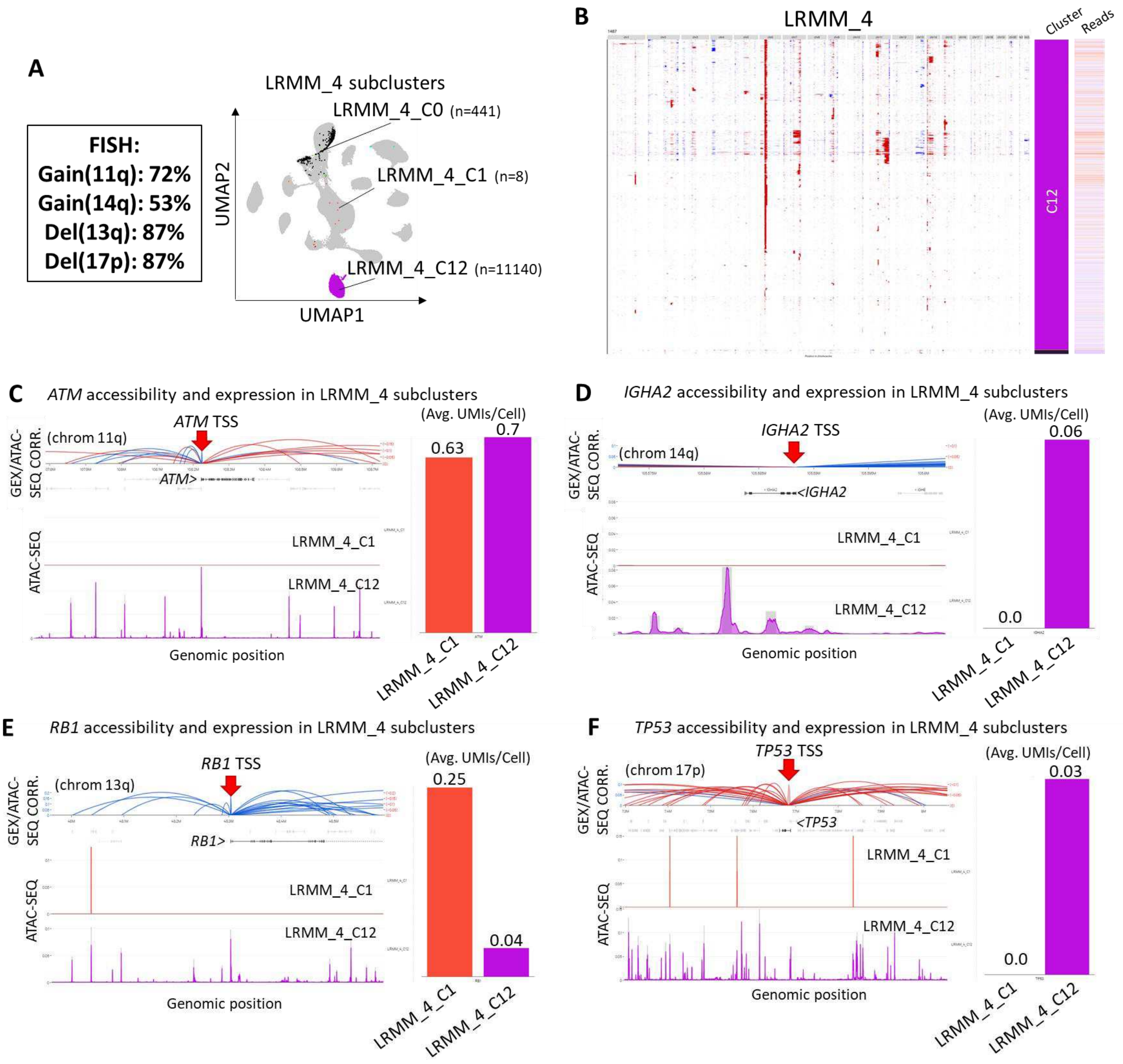

Figure S23. Differential gene accessibility and expression across LRMM_4 subclusters. This sample was identified to have Gain(11q), Gain(14q), Del(13q), and Del(17p) by FISH analysis; percentages refer to the proportion of cells harboring the abnormality. **A,** LRMM_4 subclusters – the small number of cells in cluster C1 makes the comparison less precise in subsequent analyses (a caveat for comparison). **B,** Karyogram illustrating copy number alterations (red=gain; blue=loss) from chromatin accessibility data at the single-cell level using epiAneufinder. "Cluster" = cluster number in the UMAP plot. "Reads" = normalized number of reads per cell. **C**, Left, proportion of cells per subcluster with ATAC peaks around *ATM*, a gene localized in the chromosome 11q region; right, expression of *ATM* across LRMM_4 subclusters. **D-F**, Idem **C**, for (**D**) *IGHA2*, a gene localized in chromosome 14q region; (**E**) *RB1*, a gene localized in the chromosome 13q region; and (**F**) *TP53*, a gene localized in the chromosome 17p region. TSS = transcription starting site.

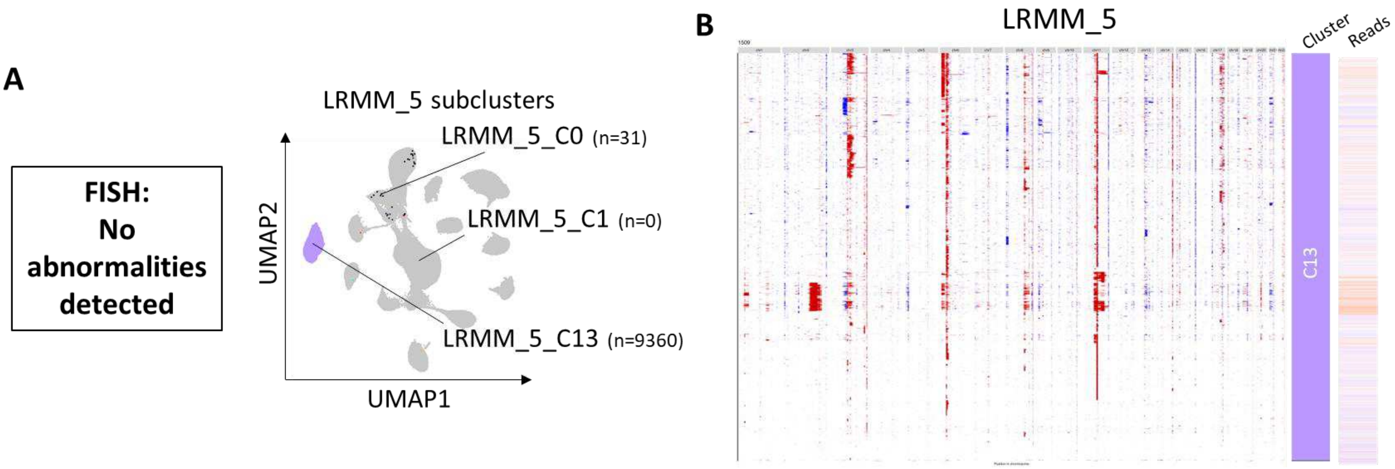

Figure S24. Distribution of LRMM_5 cells across UMAP clusters and estimation of copy number variation from chromatin accessibility data. No cytogenetic abnormalities were detected by FISH analysis. **A,** LRMM_5 subclusters. **B,** Karyogram illustrating copy number alterations (red=gain; blue=loss) from chromatin accessibility data at the single-cell level using epiAneufinder. "Cluster" = cluster number in the UMAP plot. "Reads" = normalized number of reads per cell. The lack of LRMM_5 cells in cluster C1 precluded a comparative analysis of gene accessibility/expression with cluster C13.

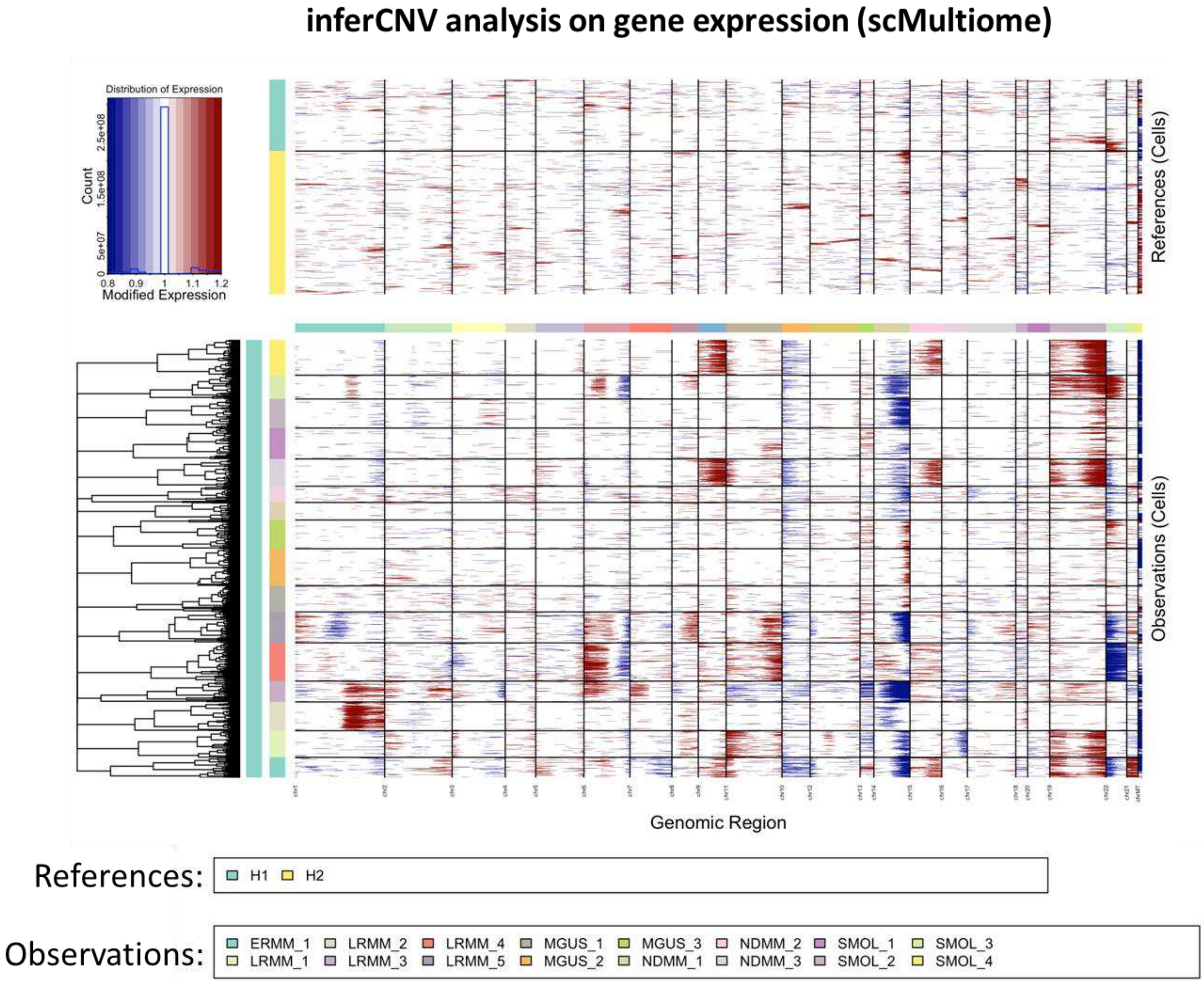

Figure S25. Copy number variation inferred by inferCNV on single cell gene expression from scMultiome data. The algorithm utilized the 2 healthy donor samples as a reference (top) to identify cytogenetic abnormalities in the samples from abnormal gene expression patterns at the single-cell level. Red = gains; blue = losses.

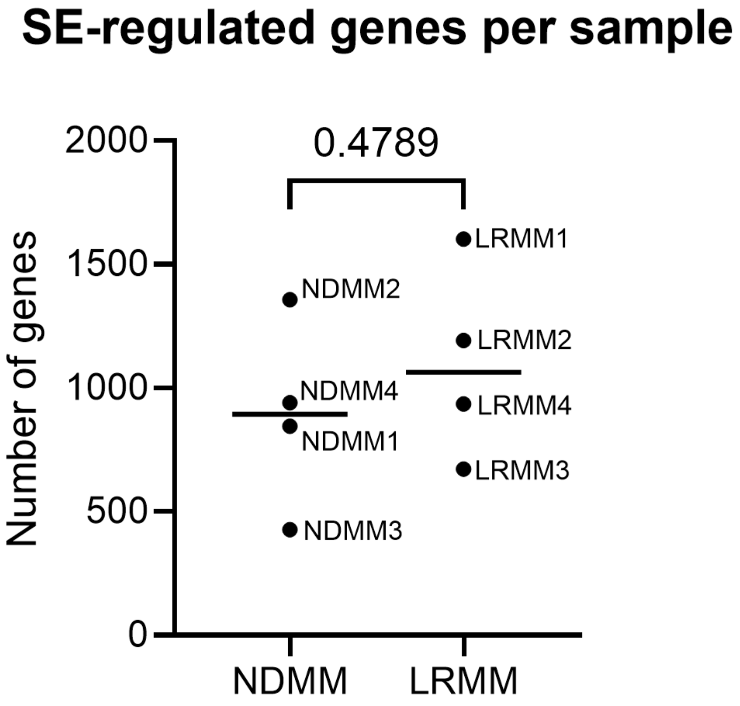

Figure S26. SE-regulated genes per sample. The total number of genes in the vicinity (within 0.5 Mb) of active super-enhancers (SEs) was determined by the ROSE algorithm across the 8 samples with CUT&Tag data (H3K27ac peaks) available. *P*-value for unpaired Student's *t*-test; the median is indicated by a horizontal line.

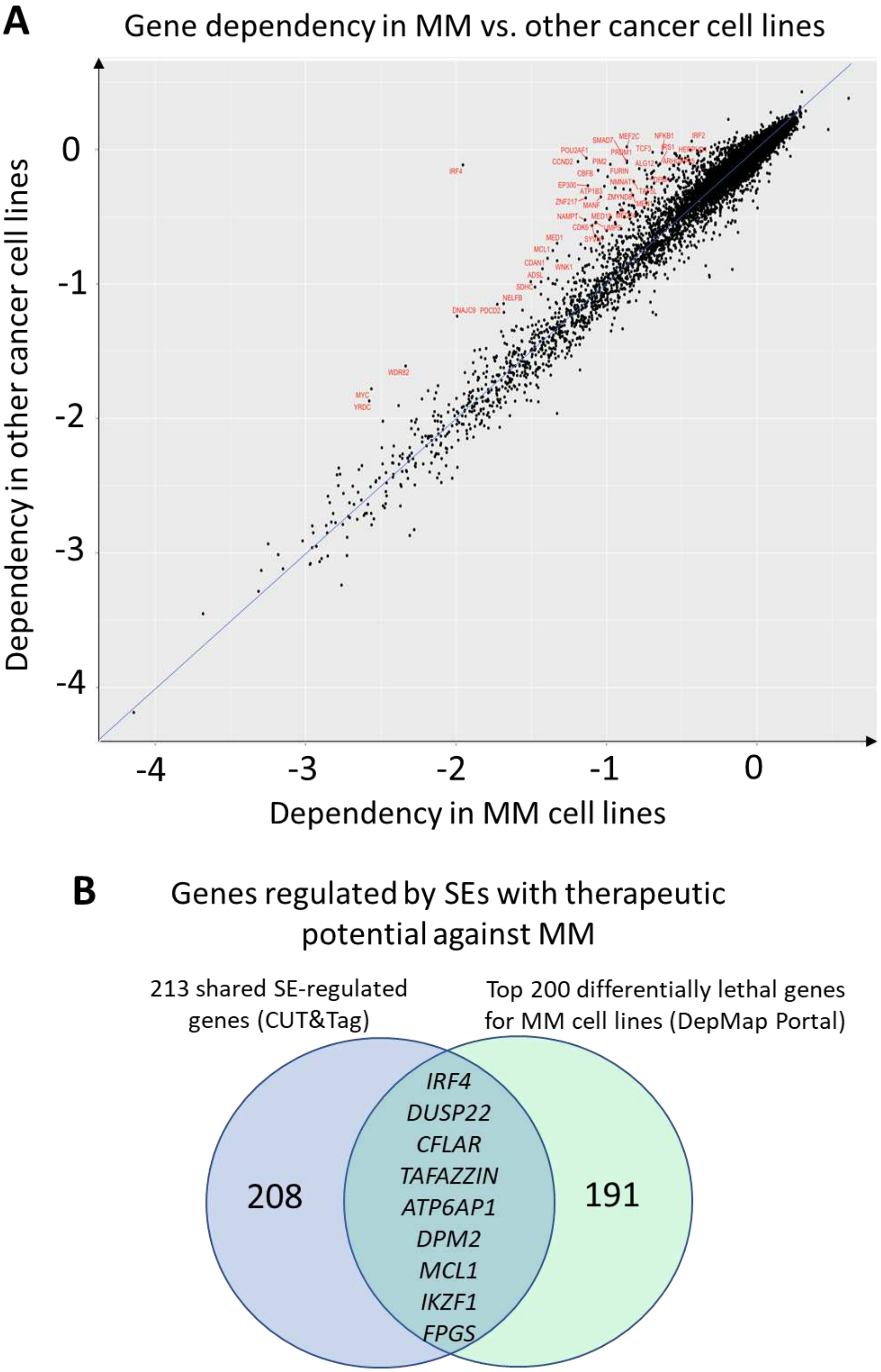

Figure S27. Differential lethality and therapeutic target candidates in MM. A, Average impact of gene knockout (KO) on the survival of MM cell lines (n=20) compared to other cancer cell lines (n=1,075; data from the DepMap portal). The 50 topmost differentially lethal genes in MM are labelled. **B**, Venn Diagram showing 9 genes near active SEs in all samples with CUT&Tag data and ranking among the top 200 most differentially lethal genes for MM cell lines compared to cancer cell lines from other tissues.

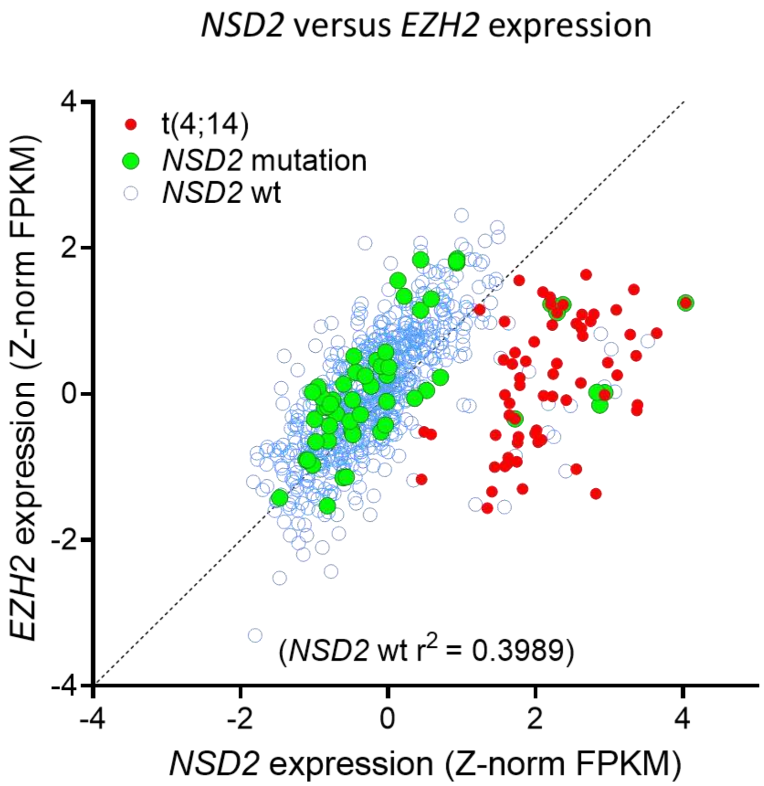

Figure S28. Transcriptional imbalance between *NSD2* and *EZH2* expression induced by t(4;14). Expression levels of *NSD2* and *EZH2* in samples with t(4;14), *NSD2* mutation, or wild type.

Supplementary Files

This is a list of supplementary files associated with this preprint. Click to download.

- TableS2MutationComparisonOtherCohorts.xlsx
- TableS1PatientDemographics.xlsx
- TableS3CytogeneticsandMutations.xlsx
- TableS6CancerHallmarksandKEGGGSEANES.xlsx
- TableS7EnrichedDBPs.xlsx
- TableS10DemographicsscMultiome.xlsx
- TableS11PioneerDBPs.xlsx
- TableS13DemographicsCUTXTag.xlsx
- TableS8QuadrantsFile.xlsx
- TableS4dNdSUnbalance.xlsx
- TableS17GeneExpxDependencyinMMlinesDepMap.xlsx
- TableS18EssentialGenesinMMcelllines.xlsx
- TableS19GeneExpressionin8226andB25.xlsx
- TableS16213SharedSEs.xlsx
- TableS5DEGs.xlsx
- TableS9CoxPHModels.xlsx
- TableS14CUTXTagListofPeaks.xlsx
- TableS15SEsROSE.xlsx
- TableS12epiAneufinderinferCNVandFISH.xlsx
